# High-Flow Nasal Oxygen Therapy After Cardiac Surgery: A Randomized Clinical Trial

**DOI:** 10.1001/jamanetworkopen.2026.5447

**Published:** 2026-04-08

**Authors:** Edward Litton, Rachael L. Parke, Shay P. McGuinness, Sarah N. Dawson, Sofia S. Villar, Siddesh S. Shetty, Julia A. Fox-Rushby, Julieann Coombes, Richard Norman, Gavin J. Murphy, Jacquita S. Affandi, Aamer B. Ahmed, Fiona E. Bottrill, Yi-Da Chiu, Luke J. Churchill, Sananta K. Dash, Anthony P. Delaney, Melissa J. P. Duckworth, Melissa J. Earwaker, Caroline R. Evans, Andrew J. Hoppington, Gudrun Kunst, Andrew J. Maiorana, Guillermo Martinez, Andrew McDonald, Ayman A. Mohammed, Neil R. Orford, David V. Pilcher, Mahesh Ramanan, Christopher M. Reid, Sivagnanavel Senthuran, Michael W. Shaw, Benjamin G. Shelley, Harjot Singh, C. Jo M. Steele, Ellen B. Temple, Marijcke W. M. Veltman-Grisenthwaite, Sumit Yadav, Nicoletta Zimbler, Vasileios Zochios, Andrew A. Klein

**Affiliations:** 1 Intensive Care Unit, Fiona Stanley Hospital, Perth, Australia; 2 Curtin School of Population Health, Curtin University, Perth, Australia; 3 Cardiothoracic and Vascular Intensive Care Unit, Te Toka Tumai Auckland, Auckland, New Zealand; 4 School of Nursing, The University of Auckland, Auckland, New Zealand; 5 Papworth Trials Unit Collaboration, Royal Papworth Hospital NHS (National Health Service) Foundation Trust, Cambridge, United Kingdom; 6 MRC (Medical Research Council) Biostatistics Unit, University of Cambridge, Cambridge, United Kingdom; 7 Department of Population Health Sciences, King’s College London, London, United Kingdom; 8 The George Institute for Global Health, Sydney, Australia; 9 College of Life Sciences, University of Leicester, Glenfield Hospital Leicester, Leicester, United Kingdom; 10 Department of Anaesthesia & Critical Care, Glenfield Hospital, University Hospitals of Leicester NHS Trust, Leicester, United Kingdom; 11 Intensive Care Unit and Critical Care Research Group, The Prince Charles Hospital, Metro North Hospital and Health Services, Brisbane, Australia; 12 Intensive Care Unit, Townsville University Hospital, Townsville, Australia; 13 Malcolm Fisher Department of Intensive Care Medicine, Royal North Shore Hospital, Sydney, Australia; 14 Faculty of Medicine and Health, The University of Sydney, Sydney, Australia; 15 Department of Anaesthetics, University Hospital of Wales, Cardiff, United Kingdom; 16 Patient representative, United Kingdom; 17 Department of Anaesthetics and Pain Therapy, King’s College Hospital NHS FT, London, United Kingdom; 18 School of Cardiovascular and Metabolic Medicine & Sciences, King’s College London, London, United Kingdom; 19 Curtin School of Allied Health, Curtin University, Perth, Australia; 20 Department of Anaesthesia and Intensive Care, Royal Papworth Hospital, Cambridge, United Kingdom; 21 Cardiothoracic Unit, James Cook Hospital, Middlesbrough, United Kingdom; 22 Intensive Care Unit, University Hospital Geelong, Geelong, Australia; 23 Deakin University School of Medicine, Geelong, Australia; 24 Department of Intensive Care, The Alfred Hospital, Melbourne, Australia; 25 The Australian and New Zealand Intensive Care Research Centre, Monash University, Melbourne, Australia; 26 School of Public Health and Preventive Medicine, Monash University, Melbourne, Australia; 27 Department of Anaesthetics, Guy’s and St Thomas’ Hospitals, London, United Kingdom; 28 West of Scotland Heart and Lung Centre, Golden Jubilee National Hospital, Glasgow, United Kingdom; 29 Academic Unit of Anaesthesia, Pain and Critical Care, University of Glasgow, Glasgow, United Kingdom; 30 Department of Anaesthesia, Queen Elizabeth Hospital, Birmingham, United Kingdom; 31 Cambridge Cancer Research Hospital, Cambridge Institute, University of Cambridge, Cambridge, United Kingdom; 32 Department of Cardiothoracic Surgery, Mater Private Hospital Townsville, Townsville, Australia; 33 Department of Anaesthesia, Royal Brompton Hospital, London, United Kingdom; 34 Department of Cardiovascular Sciences, University of Leicester, Leicester, United Kingdom

## Abstract

**Importance:**

High-flow nasal oxygen therapy (HFNOT) is used for noninvasive respiratory support following cardiac surgery despite uncertainty about its clinical effectiveness or associated costs.

**Objective:**

To determine whether prophylactic HFNOT in patients at increased risk of respiratory complications following cardiac surgery has clinical benefits compared with standard oxygen therapy (SOT).

**Design, Setting, and Participants:**

This adaptive, parallel group, randomized clinical trial collected and analyzed data from 17 cardiac surgery centers in 3 countries between October 7, 2020, and June 19, 2024. Eligible participants included adults undergoing nonemergent cardiac surgery with any of the following risk factors for pulmonary complications: chronic obstructive pulmonary disease, asthma, lower respiratory tract infection in the last 4 weeks, a body mass index of 35 or greater, or currently or recently smoking for longer than 10 pack-years. Outcome assessors were blinded. A preplanned sample size re-estimation was conducted after 300 participants completed the 90-day follow-up.

**Intervention:**

Participants were randomized at a 1:1 ratio with concealed allocation to HFNOT or SOT administered for at least 16 hours immediately after postoperative extubation.

**Main Outcomes and Measures:**

The primary effectiveness outcome was days alive and at home (DAH) without increased support compared with baseline in the first 90 days (DAH90). Any day of increased support, including at home, would provide a value of 0 for that day. Secondary outcomes included DAH90 without considering the additional support component.

**Results:**

A total of 1280 patients were recruited (mean [SD] age, 62.9 [10.5] years; 892 [69.7%] men; 640 in each group), of whom 1224 (95.6%) had complete DAH90 data. The primary outcome of median DAH90 was 0 (IQR, 0-79) for the HFNOT group and 0 (IQR, 0-87) for the SOT group (median difference, 0 [95% CI, 0-0]; *P* = .75). Secondary clinical outcomes, including DAH90 without considering whether additional support was required, were similar between groups.

**Conclusions and Relevance:**

In this randomized clinical trial of HFNOT in patients at increased risk of postoperative pulmonary complications after nonemergent cardiac surgery, HFNOT did not improve DAH90 without increased support. These findings do not support the implementation of routine prophylactic HFNOT after cardiac surgery.

**Trial Registration:**

isrctn.org Identifier: ISRCTN14092678

## Key Points

**Question:**

In patients at increased risk of pulmonary complications undergoing cardiac surgery, does prophylactic high-flow nasal oxygen therapy (HFNOT) initiated at the time of extubation have important clinical benefits vs the use of standard oxygen therapy (SOT)?

**Findings:**

In this randomized clinical trial that included 1280 adults, HFNOT did not improve clinical outcomes compared with SOT.

**Meaning:**

These findings do not support the routine implementation of prophylactic HFNOT for noninvasive respiratory support following cardiac surgery.

## Teaser

This randomized clinical trial investigates the use of high-flow nasal oxygen therapy in patients at high risk of pulmonary complications following nonemergent cardiac surgery and assesses the clinical benefits of this technique compared with standard oxygen therapy.

## Introduction

Pulmonary complications after cardiac surgery are common and increase morbidity, mortality, and use of health care resources.^1^ Atelectasis is a major driver of pulmonary complications after cardiac surgery, occurring in as many as 90% of patients. Untreated, atelectasis can cause hypoxia and respiratory failure and increase susceptibility to further complications, including pneumonia.^2^ Smoking, preexisting lung diseases such as asthma and chronic obstructive pulmonary disease, and obesity increase these risks.^3^ These patients are more likely to require escalation of respiratory support and be readmitted to critical care or the hospital after discharge.^4^

High-flow nasal oxygen therapy (HFNOT) delivers warm, humidified oxygen while generating flow-dependent positive end-expiratory pressure, reducing dead space, and improving respiratory secretion clearance.^5,6^ Compared with standard oxygen therapy (SOT), HFNOT increases patient comfort, reduces the work of breathing, and improves oxygenation.^7^ HFNOT involves the use of specialist equipment and is increasingly used for noninvasive respiratory support following tracheal extubation after cardiac surgery, despite uncertainty about whether its physiologic effects translate into clinical benefits or reductions in health care resource use.^8^ In a pilot randomized clinical trial, Zochios et al^9^ showed that routine postoperative HFNOT administration following cardiac surgery might reduce hospital stay and intensive care unit (ICU) readmission in high-risk groups, 2 important areas for cost reduction.^10^ Herein we report the results of a subsequent effectiveness trial that tested the hypotheses that HFNOT commenced at the time of extubation in the ICU would have important clinical benefits in adult patients at high risk of pulmonary complications following cardiac surgery.

## Methods

The Nasal High-Flow Oxygen Therapy After Cardiac Surgery (NOTACS) trial was an investigator-initiated, adaptive, international, multicenter, open-label, parallel-group, randomized clinical trial with blinded outcome assessment. Recruitment and follow-up were conducted in 17 centers in the United Kingdon (UK), Australia, and New Zealand between October 7, 2020, and June 19, 2024. The reporting follows the Consolidated Standards of Reporting Trials (CONSORT) reporting guideline. Research and ethics board in each country approved the study, and written informed consent was obtained preoperatively from all participants. Conduct was in accordance with the Good Clinical Practice guidelines of the International Council for Harmonisation. The trial protocol^11^ was published in advance of the interim analysis. The statistical analysis plan was registered in May 2020^12^ and updated in November 2024 before database lock and examination of any data.^13^ This protocol is available in Supplement 1.

### Participants

Eligible patients were 18 years or older, were scheduled to undergo elective or urgent cardiac surgery performed with cardiopulmonary bypass, and had 1 or more of the following risk factors for postoperative pulmonary complications: chronic obstructive pulmonary disease, asthma, lower respiratory tract infection in the last 4 weeks, a body mass index (calculated as weight in kilograms divided by height in meters squared) of 35 or greater, or current or recent heavy smoking (>10 pack-years).

### Randomization and Blinding

Randomization in a 1:1 ratio using an online tool (Sealed Envelope; Sealed Envelope Ltd) took place after commencement of cardiac surgery and prior to tracheal extubation. Randomization was stratified by center with random permuted block sizes of 4 or 6. Treating clinicians and participants were aware of the study allocation. However, outcome adjudicators were blinded.

### Trial Intervention

Participants received either HFNOT or SOT immediately following postoperative tracheal extubation in the ICU. As per the protocol (Supplement 1), HFNOT was started at 40% inspired oxygen and flow of 30 L/min then increased to 50 L/min for a duration of 5 to 10 minutes. Patients were monitored for oxygen saturation, respiratory rate, and arterial blood gas levels after 15 minutes and then per local policy afterward. If oxygen saturation was less than 93%, inspired oxygen was increased as per the respiratory escalation protocol. Standard oxygen therapy was 30% to 40% inspired oxygen and flow 2 to 6 L/min via nasal prongs or nonrebreathing mask (not humidified and not heated). If oxygen saturation was less than 93%, inspired oxygen dose was increased as per respiratory escalation protocol.

Trial adherence required treatment with the allocated intervention for a minimum of 16 hours following tracheal extubation irrespective of whether the patient remained in the ICU or was discharged to a hospital ward. Patients were allowed no more than 1 hour in total without the allocated intervention for physiotherapy and/or mobilization or transfer around the hospital. Adherence was assessed through bed medical record review of the hourly record and encouraged through site training and bedside documentation. After the initial 16-hour period, ongoing adherence with the allocated oxygen therapy was encouraged. A trial-specific extubation protocol guided oxygen administration and an escalation protocol guided treatment if the patient deteriorated (eMethods 1 in Supplement 2). Escalation of respiratory therapy was indicated if oxygen saturations were less than 93%, respiratory rate was greater than 20 breaths/min, and/or arterial Paco_2_ was greater than 7 kPa. The escalation protocol, depending on clinical need, ranged from increasing inspired oxygen concentration to transfer or readmission to high-dependency or critical care, for continuous or bilevel positive airway pressure, noninvasive ventilation (NIV), or reintubation of the trachea and mechanical invasive ventilation. All other postoperative management and care were determined by the treating clinician. Postoperative pulmonary complications were defined as any pulmonary abnormality occurring in the postoperative period that produces an identifiable disease or dysfunction that is clinically significant and adversely affects the clinical course after surgery.

### Data Collection and Follow-Up

Collected data included patient location and support, patient characteristics, oxygen use, respiratory support, adverse events, quality of life (using the 5-level version of the EuroQol 5-Dimension instrument [EQ-5D-5L]),^14^ and activities of daily living (Barthel index).^15^ Patient-level resource use of hospital, community, and social care was collected using bespoke case report forms with a mix of record extraction and patient-completed questionnaires and interviews. General practitioners were contacted for missing data on patient location where necessary.^16^

### Outcomes

The final primary clinical effectiveness outcome, determined prior to database lock, was days alive and at home in the first 90 days (DAH90) without increased support compared with baseline. This outcome allocated a score of 1 for each day a participant was in their preoperative usual place of abode and were not receiving a level of support increased compared with what they were receiving prior to the surgery, or 0 otherwise. Support categories and further detail about the calculation of DAH90 are provided in eMethods 2 and eFigure 1 in Supplement 2. Patients used a diary to record changes in place of abode and any concurrent change in support required for 90 days after surgery. A value of 0 or 1 for each day of follow-up was assumed to continue for all days to day 90 or a subsequent diary-recorded change in location, whichever occurred first. Support was asked as a binary yes or no for any support needed with activities of daily living at home.

Patients who died in the period between randomization and day 90 were assigned a DAH90 of 0, which is the most common way of calculating this outcome in previously published studies.^17^ The definition and calculation of the primary outcome were detailed in a revised statistical analysis plan published in November 2024,^13^ before database lock in December 2024. The initial primary clinical outcome of DAH90 described in the published protocol was used for the interim and sensitivity analyses. This outcome did not include the consideration of additional support, which was added due to the statistical necessity of accounting for baseline abode and to provide a more accurate and granular DAH metric and which was supported by patient feedback suggesting that this element was important to them. Secondary outcomes included clinical outcomes (ie, incidence of acute kidney injury, sepsis, stroke, or myocardial infarction; oxygenation measured by respiratory rate and oxygenation index^18^), escalation of respiratory support, DAH90 and days out of hospital to day 90, health service use, and participant-reported outcomes (EQ-5D-5L^14^ and the Barthel index) related to the surgical episode recorded at baseline, hospital discharge and 30 and 90 days after surgery.

### Statistical Analysis

NOTACS was designed as an adaptive trial with an initial sample size of 850 participants based on pilot data.^9^ It included a preplanned sample size re-estimation after 300 participants completed 90-day follow-up and conducted using the initial primary clinical outcome. This re-estimation at the interim analysis, which specifically avoided incorporating preliminary treatment effect estimates, was used to re-estimate nuisance parameters (SD of DAH90 in the SOT arm, SD of DAH90 in the HFNOT arm, treatment switch rate from SOT to HFNOT, treatment switch rate from HFNOT to SOT, overall dropout rate, and overall death rate) and used these parameters to recompute the sample size needed to achieve 90% power to detect a 2-day difference in the median DAH90 score with a type I error rate of 5%. The re-estimation was undertaken by an independent statistician in December 2022, and following consultation with the data monitoring and ethics committee and trial steering committee, the sample size was increased to 1280 participants to maintain 90% power.

The statistical analysis plans were published a priori of their respective analyses.^13,16^ In brief, the statistical analysis both at interim and final analysis were conducted using R, version 4.3.3 (R Project for Statistical Computing). For the clinical primary outcome, a Mann-Whitney Wilcoxon test was used to evaluate the difference in the DAH90 between the 2 treatment groups at a 5% significance level in the intention-to-treat population. Sensitivity analyses of the primary outcome described in the statistical analysis plan included per protocol, time on treatment, alternative definitions of the primary outcome including removal of support requirement and/or handling of death, complete cases, and examination of missing data.^13^

## Results

### Patients and Interventions

**Figure 1.**
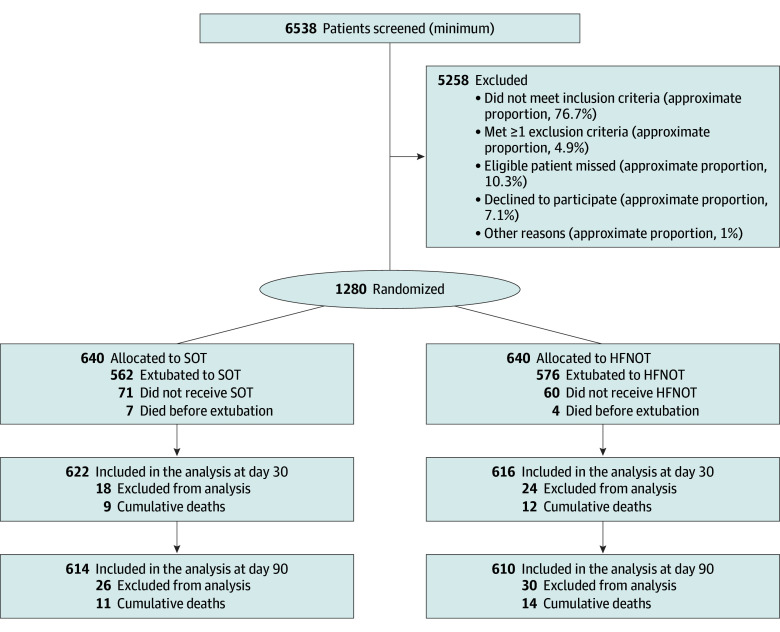
Study Flow Diagram HFNOT indicates high-flow nasal oxygen therapy; SOT, standard oxygen therapy.

Between October 7, 2020, and June 19, 2024, 1280 patients were recruited for the study, and 640 each were randomized to HFNOT and SOT (Figure 1). The mean (SD) age was 62.9 (10.5) years; 892 patients (69.7%) were men and 388 (30.3%) were women. A total of 1224 patients (95.6%) completed 90-day follow-up (missing data summarized in eTable 1 in Supplement 2). Baseline characteristics were well matched between groups (Table 1 and eTables 2-5 in Supplement 2). Compliance with randomized treatment occurred in 522 patients (81.6%) in the HFNOT group and 498 (77.8%) in the SOT group (eFigure 2 in Supplement 2). Escalation of respiratory support occurred in 338 of 625 patients (54.1%) in the HFNOT group and 354 of 628 (56.4%) in the SOT group, with the most common reason for escalation being hypoxia in both groups (Table 2).

**Table 1.** Baseline Characteristics^a^

Characteristic	HFNOT group (n = 640)	SOT group (n = 640)
Age, mean (SD), y	62.7 (10.5)	63.2 (10.5)
Sex		
Female	187 (29.2)	201 (31.4)
Male	453 (70.8)	439 (68.6)
Height, mean (SD), cm	171 (9.6)	170 (9.4)
Weight, mean (SD), kg	93.5 (21.7)	94.2 (22.4)
BMI, mean (SD)	32.1 (7.1)	32.6 (7.2)
EuroSCORE-2, median (IQR)^b^	1.6 (1.70)	1.7 (1.96)
ARISCAT score, mean (SD)^c^	51.8 (6.2)	51.3 (5.6)
COPD	124 (19.4)	102 (15.9)
Asthma	172 (26.9)	172 (26.9)
BMI >35	250 (39.1)	269 (42.0)
Current smoke	189 (29.5)	185 (28.9)
Lower respiratory tract infection in last 4 wk	23 (3.6)	14 (2.2)
Diabetes	212 (33.2)	173 (27.0)
Myocardial infarction	231 (36.1)	219 (34.2)
Chronic kidney disease	35 (5.5)	46 (7.2)
Chronic liver disease	12 (1.9)	11 (1.7)
Previous CVA or TIA	50 (7.8)	58 (9.1)
Neurologic conditions	34 (5.3)	27 (4.2)
Cardiac surgical procedures		
Elective	386 (60.3)	375 (58.6)
Urgent	253 (39.5)	264 (41.3)
CABG	400 (62.5)	397 (62.0)
Valve	308 (48.1)	300 (46.9)
CABG plus valve	88 (13.8)	77 (12.0)
Aorta	35 (5.5)	48 (7.5)
Other	30 (4.7)	35 (5.5)
First-time surgery	616 (96.3)	610 (95.3)
Repeat sternotomy surgery	23 (3.6)	29 (4.5)
Duration of surgery, mean (SD), min	255 (76)	254 (77)
Cardiopulmonary bypass time, mean (SD), min	105 (44)	109 (64)
Aortic cross-clamp time, mean (SD), min	78 (38)	78.2 (48)
Return to theater^d^	28 (4.4)	38 (5.9)

Abbreviations: ARISCAT, Assess Respiratory Risk in Surgical Patients in Catalonia; BMI, body mass index (calculated as weight in kilograms divided by height in meters squared); CABG, coronary artery bypass grafting; COPD, chronic obstructive pulmonary disease; CVA, cerebrovascular accident; EuroSCORE, European System for Cardiac Operative Risk Evaluation; HFNOT, high-flow nasal oxygen therapy; SOT, standard oxygen therapy; TIA, transient ischemic attack.

^a^ Unless indicated otherwise, data are presented as No. (%) of patients.

^b^ Scores range from 0 to 100, with higher scores indicating greater risk of mortality after surgery.

^c^ Scores range from 0 to 123, with higher scores indicating greater risk of pulmonary complications after anesthesia.

^d^ Indicates return to theater for bleeding or revision surgery in the first 30 days after initial surgery.

**Table 2.** Key Measures of Process—ROX Index and Escalations of Respiratory Support

Measure	HFNOT group (n = 640)	SOT group (n = 640)
Postextubation ROX index, median (IQR)^a^		
2 h	16.8 (13.3-20.8)	18.0 (14.2-22.5)
6 h	17.0 (13.2-22.0)	19 (15.0-24.6)
12 h	16.7 (13.6-20.9)	18.5 (14.5-23.4)
24 h	17.0 (13.5-21.3)	18.0 (14.7-23.1)
48 h	19.2 (14.7-23.6)	19.5 (15.6-24.5)
Reason for escalation of respiratory support, No. (%) of episodes		
Tachypnoea	93/1386 (6.7)	47/1294 (3.6)
Hypoxia	841/1386 (60.7)	948/1294 (73.3)
Medical deterioration	43/1386 (3.1)	34/1294 (2.6)
Clinical decision	82/1386 (5.9)	76/1294 (5.9)
Hypercarbia	20/1386 (1.4)	21/1294 (1.6)
Atelectasis or pneumonia	14/1386 (1.0)	0/1294
Missing	293/1386 (21.1)	168/1294 (13.0)

Abbreviations: HFNOT, high-flow nasal oxygen therapy; SOT, standard oxygen therapy; ROX, respiratory rate and oxygenation.

^a^ Defined as the ratio of oxygen saturation as measured by pulse oximetry/Fio_2_ to respiratory rate: ROX2 ≤ (2h_Spo_2_/2h_Fio_2__percent)/(2h_respiratory_rate) × 1.

### Primary Outcome

**Figure 2.**
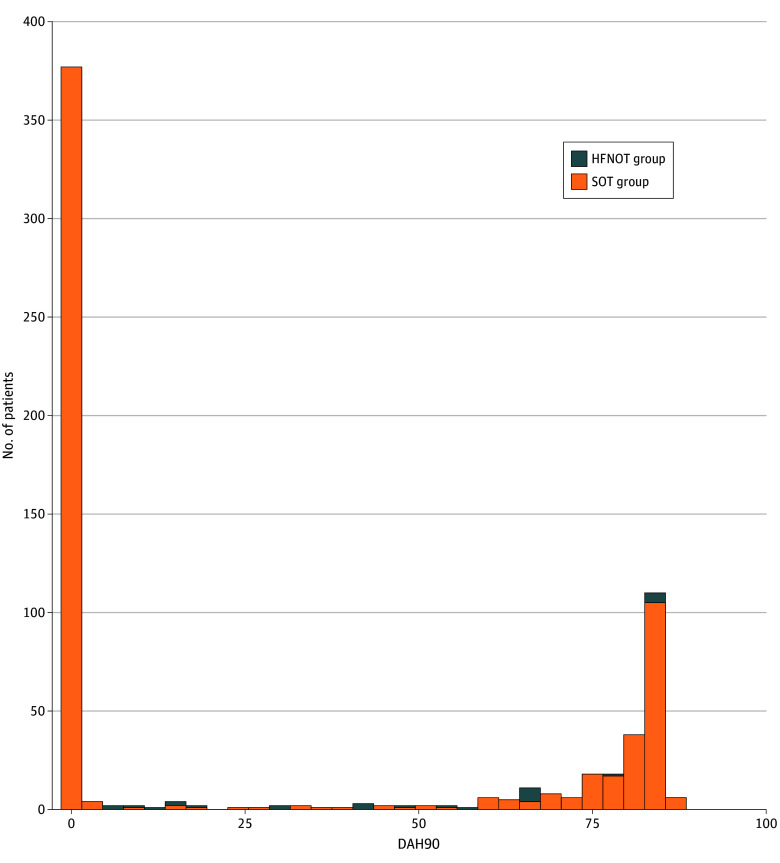
Histogram Showing Distribution of Days Alive and at Home in the First 90 Days (DAH90) HFNOT indicates high-flow nasal oxygen therapy; SOT, standard oxygen therapy.

The primary outcome was observed for 1224 patients (95.6%). The median DAH90 without additional support in the HFNOT and SOT groups was 0 (IQR, 0-79) and 0 (IQR, 0-87), respectively. The difference in the median was 0 (95% CI, 0-0; *P* = .75) (Figure 2). The findings in a number of sensitivity analyses, including the per-protocol and time on treatment analyses and days alive and out of hospital were similar and showed no treatment effect.

### Secondary Outcomes and Subgroup Analyses

The median numbers of days alive and out of hospital were similar between groups (82 [IQR, 77-84] in the HFNOT group vs 82 [IQR, 78-84] in the SOT group) (Table 3). Secondary outcomes including respiratory rate and oxygenation indices were similar between groups (Table 3 and eTables 6 and 7 in Supplement 2). Requirements for reintubation and continuous positive airway pressure were similar in both groups (Table 3). The EQ-5D-5L and the Barthel index at 90 days were similar between the 2 groups (eTables 11-16 in Supplement 2).

**Table 3.** Outcomes to 90 Days Following Cardiac Surgery by Treatment Group

Outcome	HFNOT group (n = 640)	SOT group (n = 640)
Days at home without increased support in 90 d, median (IQR)	0 (0-79)	0 (0-87)
Days alive and out of hospital in 30 d, median (IQR)	22 (18-24)	23 (18-24)
Days alive and out of hospital in 90 d, median (IQR)	82 (77-84)	82 (78-84)
Mortality, No./total No. (%)	14/640 (2.2)	11/640 (1.7)
Timing of death, median (IQR), postrandomization d	14.5 (5.0-43.8)	6.0 (1.5-18.5)
Hospital stay, median (IQR), d	7.1 (6.0-10.1)	7.1 (5.9-10.7)
ICU stay, median (IQR), d^a^	2.0 (0.9-3.9)	1.8 (0.8-3.6)
ICU readmissions, No./total No. (%)^a^	31/640 (4.8)	27/640 (4.2)
Reintubation, No./total No. (%)	21/625 (3.4)	15/630 (2.4)
CPAP, No./total No. (%)	50/625 (8.0)	59/630 (9.4)
Discharge destination, No./total No. (%)		
Home	548/603 (90.9)	563/617 (91.2)
Hospital	20/603 (3.3)	29/617 (4.7)
Residential home	1/603 (0.2)	1/617 (0.2)
Nursing home	1/603 (0.2)	0
Relative’s home	0	0
Other	21/603 (3.5)	14/617 (2.3)
Readmitted to hospital, No./total No. (%)	124/598 (20.7)	128/604 (21.2)
No. of hospital readmissions per patient, mean (SD)	0.3 (0.6)	0.3 (0.7)
Incidence of stroke, No./total No. (%)	2/640 (0.3)	8/640 (1.2)
Incidence of sepsis, No./total No. (%)	16/640 (2.5)	11/640 (1.7)
Incidence of acute kidney injury, No./total No. (%)	18/640 (2.8)	24/640 (3.8)
Incidence of myocardial infarction, No./total No. (%)	3/640 (0.5)	7/640 (1.1)
Incidence of postoperative pulmonary complications, No./total No. (%)	84/638 (13.2)	108/637 (17.0)

Abbreviations: CPAP, continuous positive airway pressure; HFNOT, high-flow nasal oxygen therapy; ICU, intensive care unit; SOT, standard oxygen therapy.

^a^ Includes postoperative recovery unit, ICU, and high-dependency unit.

There was no difference in subgroup analyses based on individual countries or individual centers (UK only), including discharge destination and resource use (eTables 17-19 in Supplement 2). Adverse events and serious adverse events were similar in both groups, and detailed prespecified safety outcomes are presented in eTables 1 and 20-22 in Supplement 2.

## Discussion

In this international randomized clinical trial including patients at increased risk of pulmonary complications after nonemergent cardiac surgery, prophylactic postextubation HFNOT and SOT resulted in similar DAH90. These findings were consistent across prespecified sensitivity and secondary analyses. The results of the between-group comparisons were consistent across all prespecified measures of effectiveness providing high-quality evidence that a clinically important benefit from prophylactic HFNOT in this population is unlikely.

The primary outcome was refined while the trial was in progress. The addition of considering support to the established DAH measure was driven by the statistical necessity of accounting for baseline abode and to provide a more accurate and granular DAH metric, supported by consumer feedback that considered patient level of function of greater importance than their place of abode. A major resultant advantage was the capability of differentiating health-related and non–health-related causes for assigning a value of 0 in the DAH calculation; for example, avoiding a 0 value for a day staying closer to the hospital purely because of geographical distance from home or going on holiday after recovery. However, measurement of this modification to the primary end point presented precision and reproducibility challenges. Data collection relied on patients accurately reporting changes in location and concurrent needs for support, introducing the potential for approximation bias. Although an adjudicated objective definition of location and support as part of DAH90 was prespecified in the second iteration of the statistical analysis plan, the location diary was designed to record changes in location and support (ie, with a start and end date for each entry), and the diary was not designed to collect data on a daily basis. While daily collection of location and support data may reduce potential approximation bias, it is acknowledged that this extra burden of data collection is likely to increase the amount of missing data, and as a result reduce the number of patients for whom DAH90 can be calculated. Two key takeaways for future studies are the necessity of recording location and support needs as distinct, separate variables to enhance data granularity and investigating whether the increased support needed at home is sustained or largely confined to a short period following home discharge.

The NOTACS results are consistent with a recent systematic review and meta-analysis of 9 small, mostly single-center trials^8^ in which HFNOT increased oxygenation as intended but did not have consistent clinical benefits. That systematic review found a decrease in treatment escalation with HFNOT that we did not observe and no difference in the rates of tracheal reintubation, mortality, or hospital or ICU stay that we also observed. NOTACS recruited a larger sample than all the previous randomized clinical trials of HFNOT in cardiac surgery combined, with the additional strengths of an international multicenter design, measurement of postdischarge patient-centered outcomes, and a comprehensive health economic analysis.

### Limitations

Several limitations of this study require consideration. First, the primary outcome was refined throughout the trial. However, the eventual DAH90 was prespecified prior to database lock. Second, the interim analysis was conducted on the original DAH90. However, there were no between-group differences for either, and the 95% CIs of the estimates suggest a clinically important difference is highly unlikely. Third, the modified primary outcome would place the same value on minor and major increased support requirements. However, there were no differences in secondary outcomes, including either activities of daily living or quality of life measures. Last, the study evaluated prophylactic HFNOT administration in a population after nonemergent cardiac surgery; whether clinically important differences occur in other populations is uncertain.

## Conclusions

In this randomized clinical trial of prophylactic postoperative HFNOT in patients at higher risk of developing respiratory complications after elective cardiac surgery, we found that HFNOT did not improve important clinical outcomes compared with SOT. These findings do not support routine implementation of prophylactic HFNOT following cardiac surgery.

## Notes

Supplement 1.
Trial Protocol

Supplement 2. **eMethods 1. **Trial Escalation of Respiratory Therapy Protocol **eMethods 2. **Calculation of Days at Home in the First 90 Days after Surgery (DAH90) **eFigure 1.** Display of Patient Locations Over 90 Days for a Subset of Patients in the NOTACS Trial **eTable 1. **Missing Data for Primary and Secondary Analysis Variables **eTable 2.** Number of Patients at Each Study Site by Treatment Arm and Study Stage **eTable 3.** Baseline Ethnicity by Treatment Arm **eTable 4. **Baseline Residence by Treatment Arm **eTable 5. **Baseline Participant and Family Resource Use Questionnaire by Treatment Arm **eFigure 2. **Adherence With Randomized Therapy, Time on Randomized and Nonrandomized Treatment Arms for Patients Randomized to Nasal High-Flow Nasal Oxygen Therapy (NHFOT) or Standard Oxygen Therapy (SOT) **eTable 6. **Median Quantile Regression Between Response Variable DAH90 and Treatment Group, Adjusted for Other Selected Baseline Variable **eTable 7. **Comparison of Postextubation ROX Indexes by Treatment Group **eTable 8. **Baseline EQ-5D-5L Questionnaire by Treatment Arm **eTable 9. **Discharge EQ-5D-5L Questionnaire by Treatment Arm **eTable 10. **Day 30 EQ-5D-5L Questionnaire by Treatment Arm **eTable 11. **Day 90 EQ-5D-5L Questionnaire by Treatment Arm **eTable 12.** Baseline Barthel Index Questionnaire by Treatment Arm **eTable 13.** Discharge Barthel Index Questionnaire by Treatment Arm **eTable 14.** Day 30 Barthel Index Questionnaire by Treatment Arm **eTable 15.** Day 90 Barthel Index Questionnaire by Treatment Arm **eTable 16. **Barthel Index Questionnaire at Baseline, Discharge, Day 30 and Day 90, Ignoring Treatment Arm **eTable 17. **Discharge Participant and Family Resource Use Questionnaire by Treatment Arm **eTable 18. **Discharge Destination by Treatment Arm **eTable 19. **Frequency and Duration (Days) of Nonhospital Stays Away From Home Following Discharge From Index Hospital Admission by Treatment Arm **eTable 20. **Incidence Rate of Serious Adverse Events of Special Interest by Treatment Arm **eTable 21.** Summary of Adverse Events (Excludes Serious Adverse Events) By Treatment Arm **eTable 22. **Summary of Serious Adverse Events by Treatment Arm

Supplement 3.
Nonauthor Collaborators

Supplement 4.
Data Sharing Statement
