# Supplementary material for: High-Flow Nasal Oxygen Therapy After Cardiac Surgery: A Randomized Clinical Trial
Source: JAMA Netw Open. 2026 Apr 8;9(4):e265447. doi: 10.1001/jamanetworkopen.2026.5447 (PMC13063085; doi:10.1001/jamanetworkopen.2026.5447)
Supplement: Supplement 1. — Trial Protocol [file jamanetwopen-e265447-s001.pdf]

NHS
Royal Papworth Hospital
NHS Foundation Trust
Royal Papworth Hospital NHS Foundation Trust
Papworth Road
Cambridge Biomedical Campus
Cambridge
CB2 0AY
Tel: 01223 638000

Short Title: **Nasal High-Flow Oxygen Therapy After Cardiac Surgery (NOTACS) Study:**

Title: **Effect of High-Flow Nasal Therapy on Patient-Centred Outcomes in Patients at High Risk of Postoperative Pulmonary Complications After Cardiac Surgery: A Multicentre Randomised Controlled Trial.**

Protocol

CHIEF INVESTIGATOR: Dr Andrew Klein (Royal Papworth Hospital)

Trial Sponsor: Royal Papworth Hospital NHS Foundation Trust
Funders:
UK: National Institute for Health Research (NIHR Health Technology Assessment).
Australia: Medical Research Future Fund, Australia
New Zealand: Green Lane Research and Educational Fund

Protocol Identification Number: P02590
Study IRAS ID: 278290

Protocol Version/Date:

Version: 5.0
Dated: **8th September 2023**

Contents

TRIAL OVERVIEW

Trial Summary

Protocol Amendments

Abbreviations

1. Trial Overview
   - 1.1 Background
   - 1.2 Research Aims
   - 1.3 Trial Design

2. Patient Recruitment Criteria
   - 2.1 Inclusion Criteria
   - 2.2 Exclusion Criteria
   - 2.3 Coronavirus Advice

3. Visit Schedule
   - 3.1 Schedule of Events
   - 3.2 Screening (Visit 1)
   - 3.3 Baseline (Visit 2)
   - 3.4 Randomisation (Visit 3)
   - 3.5 Discharge (Visit 4)
   - 3.6 Post-Discharge (Visit 5 & 6)
   - 3.7 Withdrawal From Trial

4. Data Collection Methods
   - 4.1 Baseline Data
   - 4.2 Primary Outcome Data Collection (DAH90)
   - 4.3 Exploratory Secondary Outcome Data Collection
     - 4.3.1 EQ-5D-5L Questionnaire
     - 4.3.2 BARTHEL Questionnaire
     - 4.3.3 Health Service & Resource Use
   - 4.4 Case Report Form Completion
     - 4.4.1 Source Documents
     - 4.4.2 Errors and Corrections
     - 4.4.3 Retention of Documents

5. Data Analysis
   - 5.1 Sample Size
     - 5.1.1 Initial Sample Size Calculation
     - 5.1.2 Adaptive Design
     - 5.1.3 Interim Analysis
   - 5.2 Recruitment & Retention
   - 5.3 Statistical Analysis
     - 5.3.1 Methods in analysis to handle protocol non-adherence and any statistical methods to handle missing data

5.4 Health Economics Analysis

6. Adverse & Serious Events
6.1 Recording and Reporting
6.2 Expected Adverse Events

7. Management and Governance
7.1 Sponsorship
7.2 Project Management
7.3 Trial Steering Committee (TSC)
7.4 Data Monitoring and Ethics Committee (DMEC)
7.5 Monitoring and Audit

8. Ethical Considerations

9. Data Protection and Patient Confidentiality

10. Publication policy

11. References

12. Appendices
12.1 Appendix 1: Exploratory Secondary Outcome Definitions
12.2 Appendix 2: Trial Extubation Protocol
12.3 Appendix 3: Trial Escalation of Respiratory Therapy Protocol
12.4 Appendix 4: International Management and Governance Structure
12.5 Appendix 5. Telephone Interview Escalation protocol NOTACS study
12.6 Appendix 6: Details of Interim Analysis

List of Figures

Figure 1. Patient Flow Diagram
Figure 2. Follow-up Flow Diagram
Figure 3. AE & SAE Flow diagram

List of Tables

Table 1. Schedule of Events
Table 2. Recommended sample size from Interim sample size re-estimation and course of action
Table 3. Table of Expected Adverse Events

Trial Summary

Trial Title	Effect of High-Flow Nasal Therapy on Patient-Centred Outcomes in Patients at High Risk of Postoperative Pulmonary Complications after Cardiac Surgery: A Multicentre Randomised Controlled Trial
Short Title	Nasal High-Flow Oxygen Therapy After Cardiac Surgery (NOTACS) study
Trial Sponsor	Royal Papworth Hospital NHS Foundation Trust, Papworth Road, Cambridge Biomedical Campus, Cambridge, CB2 0AY<br>Tel: 01223 638000
Trial Registration	The trial has been registered with ISRCTN<br><br>Trial ID: ISRCTN14092678<br><br>Date registered: 13/05/2020
Trial Funding	For UK: National Institute for Health Research (NIHR Health Technology Assessment). Unique Award Identifier NIHR128351<br><br>For Australia: Medical Research Future Fund, Australia (APP2006100)<br><br>For New Zealand: Green Lane Research and Educational Fund (21/23/4159)
Trial Design	An adaptive, multicentre, parallel group randomised controlled clinical trial with embedded cost-effectiveness analysis.
Trial Aims	<u>Primary Aim:</u><br><br>• To determine if prophylactic use of high-flow nasal therapy (for a minimum of 16 hours(inclusive of up to one hour off randomised therapy, if clinically required, see section 3.4) after tracheal extubation) increases days alive and at home in the first 90 days after surgery, for adult patients undergoing cardiac surgery who are at high risk of postoperative pulmonary complications.<br><br>• Health economic analysis to estimate the incremental cost-effectiveness and cost-utility of HFNT versus standard oxygen therapy at 90 days, from the view-point of the public sector, NHS and patients.

	<u>Exploratory Secondary Aims:</u><br><br>• Health economic analysis to estimate the incremental cost-effectiveness and cost-utility of HFNT versus standard oxygen therapy at 30 days.<br><br>• Statistical Analysis to determine if prophylactic use of high- flow nasal oxygen:<br>➢ Reduces mortality, pulmonary complications, intensive care re-admission rate, length of hospital and intensive care stay.<br>➢ Reduces incidence of major complications including sepsis, acute kidney injury (AKI), myocardial infarction and stroke.<br>➢ Reduces readmission to hospital rate.<br>➢ Improves oxygenation as measured by the ROX Index (as defined as Sp02/Fi02 to respiratory rate ratio).<br>➢ Improves patient-centred outcomes as measured using the EQ-5D-5L.<br>➢ Reduce patient level of assistance needed with activities of daily living as measured using BARTHEL questionnaire.<br>➢ Improves quality of survival as measured using ED-5D-5L Quality adjusted life years (QALYs)<br>➢ Reduces health service and resource use.
Original Trial Participants	850 – 1152
Trial Participants After Interim Sample Size Re-Estimation	1280
Inclusion/ Exclusion Criteria	<u>Inclusion Criteria:</u><br><br>• Aged 18 years or over.<br>• Undergoing any elective or urgent first-time or redo cardiac surgery performed on cardiopulmonary bypass<br>• Have one or more clinical risk factors for postoperative pulmonary complications (COPD, asthma, lower respiratory tract infection in last 4 weeks as defined by use of antibiotics, body mass index ≥35 kg/m^2 , current (within the last 6 weeks)

	heavy smoker (> 10 pack years)) (47, 48).<br><br><u>Exclusion Criteria</u><br><br>• Requiring home oxygen therapy.<br>• Deep hypothermic circulatory arrest planned<br>• Contraindication to HFNT, e.g. nasal septal defect.<br>• Requirement for home ventilatory support (including: HFNT, CPAP, BiPAP)<br>• Requiring emergency cardiac surgery defined as surgery required within 24 hours of the decision to operate.<br>• Patients not fluent in English.
Intervention	Prophylactic use of HFNT for a minimum of 16 hours (inclusive of up to one hour off randomised therapy, if clinically required, see section 3.4) started immediately after tracheal extubation.
Standard of Care Treatment	Non humidified oxygen given via nasal prongs (1-2l) or re breathing mask (2-10l) through board tubing.
Follow-up Visits	Discharge (questionnaires +30 days), 30 (+7 days) and 90 days (+14 days) postoperative

Protocol Amendments

Amendment Reference	Dated	Summary of Changes
Substantial Amendment 1	17th December 2020	Clarification of data collection and other logistical clarifications, clarifications of the statistical methods and analysis plans, update of safety reporting section, update to extubation protocol (Appendix 2), inclusion of COVID-19 guidance and recommendations and correction of typographical errors.
Substantial Amendment 2	19th January 2022	Submission of amended Protocol to: Revise inclusion criteria #2 to include all elective and urgent first-time and redo cardiac surgery performed using cardiopulmonary bypass; reference the involvement of Australia and New Zealand together with a new appendix (Appendix 4) to show the international management and governance structure; add Follow-up Telephone escalation Protocol (Appendix 5); ensure it is clear that incidence of AKI is an exploratory secondary outcome; re-format and clarify the schedule of activities table; clarify the timing of randomisation; add clarifications to the data analysis, statistical methods and safety sections and correction of formatting and typographical errors throughout document.
Substantial Amendment 3	15th November 2022	To extend the tolerance window for the 90-day follow-up from +7 days to +14 days which is more relative to the time period and to add a number of expected adverse events to Table 3 and clarify the management of ongoing adverse events after the 90 day follow-up.
Substantial Amendment 4	8th September 2023	Updates to the protocol include: increase to the maximum sample size following the pre-planned interim sample size re-estimation; correction of the smoking pack years calculation and information included on e-cigarettes and vaping; addition of wording to explain that participants are allowed up to an hour off randomised therapy for transfers around the hospital and/or physio mobilisation; updated guidance on timing of follow up phone calls if patients are uncontactable on the first call; updated guidance to ask site staff to contact patients to complete discharge questionnaires out to +30 days post-discharge; clarification to plan B of the Escalation of Respiratory Therapy Protocol in Appendix 3; update of Appendix 4 to clarify the International Management and Governance Structure and addition of Appendix 6 to provide details on the outcome of the interim sample size re-estimation

Abbreviations

Abbreviation	Definition
AE	Adverse Event
ARDS	Acute Respiratory Distress Syndrome

BiPAP	Bilevel Positive Airway Pressure
BMI	Body Mass Index
CABG	Coronary Artery Bypass Graft
CNS	Central Nervous System
COPD	Chronic Obstructive Pulmonary Disease
CPAP	Continuous Positive Airway Pressure
CPB	Cardiopulmonary Bypass
CRF	Case Report Forms
CRN	Clinical Research Network
CTU	Clinical Trials Unit
DAH	Days alive and at Home
DAH30	Days alive and at Home 30 days post-operative
DAH90	Days alive and at Home 90 days post-operative
DMEC	Data Monitoring & Ethics Committee
ECG	Electrocardiogram
eGFR	Estimated Glomerular Filtration Rate
Fi02	Fraction of Inspired Oxygen
GP	General Practitioner
HDU	High Dependency unit
HFNT	High-Flow Nasal Therapy
IABP	Intra-aortic Balloon Pump
ICER	Incremental cost-effectiveness
ICH	International Council for Harmonisation
ICU	Intensive Care Unit
ITT	Intention To Treat
LOS	Length of Stay
NICE	National Institute for Health and Clinical Excellence
NICOR	National Institute of Cardiovascular Outcomes Research
Physio	Physiotherapy
PI	Principle Investigator
PPE	Personal Protective Equipment
PTUC	Papworth Trials Unit Collaboration
QUALYS	Quality Adjusted Life Years
R&D	Research & Development
RCT	Randomised Controlled Trial
REC	Research Ethics Committee
RR	Respiratory Rate
sCR	Serum Creatinine
SD	Standard Deviation
SOT	Standard Oxygen Therapy
Sp02	Peripheral Capillary Oxygenation Saturation
SUSAR	Serious Unexpected Adverse Reaction
TIA	Transient Ischaemic Attack
TSC	Trial Steering Committee
VF	Ventricular Fibrillation
VT	Ventricular Tachycardia

1. Trial Overview

1.1 Background

Patients undergoing cardiac surgery are at significant risk of postoperative pulmonary complications (PPC) that may lead to prolonged intensive care unit (ICU) and hospital stay and increase mortality (1). The incidence of respiratory complications may be three to four times more common in patients

with intrinsic respiratory disease and lower airway obstruction (including asthma or chronic obstructive pulmonary disease (COPD)), or obese patients or current heavy smokers (> 10 pack years) (2). These patients often develop lower respiratory tract infections, with impaired oxygenation/ventilation and prolonged requirement for ventilatory support. They are more likely to require escalation of respiratory support and readmission to intensive care unit (ICU) during recovery from surgery (3-5).

High-flow nasal therapy (HFNT) is increasingly used as a non-invasive form of respiratory support (6). It delivers low level, flow-dependent positive airway pressure, and is much better tolerated by patients than alternatives such as continuous positive airway pressure (CPAP) or non-invasive ventilation (7). Patients can talk, eat, drink and walk whilst using HFNT. However, there is equipoise regarding its prophylactic use and effect on important patient-centred outcomes, hence the rationale for this trial. Recent systematic reviews in non-cardiac (8) and cardiothoracic (9) surgery concluded that HFNT could reduce respiratory support and pulmonary complications, and could be safely administered.

The first single-centre randomised controlled trial investigating the effect of HFNT on clinically relevant outcomes in cardiac surgical patients with pre-existing lung disease [including COPD or asthma] or a higher risk for pulmonary complications (including obesity (BMI > 35 kg.m^2), recent respiratory tract infections (in preceding four weeks) or current heavy smoking) (10) was performed at the Royal Papworth Hospital NHS Foundation Trust as a pilot for a larger randomised controlled trial (RCT). It was observed that prophylactic use of HFNT in these higher-risk cardiac surgical patients was well tolerated with treatment compliance of 75% in the treatment arm, with 12% crossover from standard oxygen to HFNT and 25% crossover from HFNT to standard oxygen. In total 99% of patients provided outcome data at 90 days. Prophylactic use of HFNT in cardiac surgical patients at higher risk for pulmonary complications demonstrated a reduced length of hospital stay by 29% (95% CI 11-44%, p=0.012) and intensive care unit (ICU) re-admission rate from 14% to 2% (p=0.026)(10). This pilot study provided evidence of feasibility and pilot data to help better design the larger NOTACS RCT.

Hospital and ICU stay are likely to form a large portion of the total cost of patient care and therefore provide an important focus for cost reduction (11). However; no studies on HFNT in cardiothoracic surgery have yet provided adequate costing. While related economic papers in the wider literature (pre-term infants, ICU patients) appear to support the potential for cost saving, significant caveats are given (12, 13). The proposed trial will therefore provide not only the first primary data on the cost and cost-effectiveness analysis of HFNT for cardiac surgery, but it may also be of interest for HFNT after other types of major operations, such as laparotomy and thoracotomy.

Burden of disease:

Figures from the National Institute for Cardiovascular Outcomes Research (NICOR) database (14) show that, over the last 7 years, an average of 36,505 patients a year underwent cardiac surgery in the UK. Around 26% of these patients would have fulfilled the trial inclusion criteria and qualified as high risk for postoperative pulmonary complications and prolonged hospital stay. This equates to approximately 9,500 patients at risk for postoperative pulmonary complications per year in the UK.

Why this research is needed now:

Enhanced recovery after cardiac surgery is an emerging and important concept in perioperative care, designed to reduce complications, hospital stay and health service and resource use (15, 16) Evidence to support the routine use of HFNT will inform the development of effective enhanced recovery care bundles. However, before the intervention is recommended for routine NHS use in cardiac surgery patients at high risk of pulmonary complications, whether it improves patient-related outcomes and is cost effective in a UK setting needs to be assessed.

Potential NHS cost savings:

Data from the pilot study showed that patients, at high risk of postoperative pulmonary complications receiving prophylactic HFNT stayed on average 2 days less in hospital and ICU re-admission was reduced from 15% to 2% when compared to similar high risk patients receiving standard oxygen therapy. If this pilot data is extrapolated to the eligible UK population there is the potential to save

19,000 hospital bed days and 1235 re-admissions to ICU each year, each with a median ICU stay of 4 days [the target set by the Getting It Right First Time (GIRFT) was 3.2 days]. Such savings in ICU and surgical ward bed days would allow either more patients to be treated within the same cardiac surgery resource in the same number of in-patient beds or alternatively allow a reduction in capacity in NHS cardiac surgery beds, thus freeing up resources to treat other patients (termed 'notional financial opportunity' in the GIRFT cardiothoracic surgery report). Using cost data from the GIRFT report (17) the new intervention could potentially achieve an NHS cost saving of £6,935,000 per year in surgical ward bed days (at a cost of £365 per day) and a further saving, from reduced readmission to critical care, of £6,224,000 (£1260 / day, median 4 days and 1235 re-admissions) per year.

1.2 Research Aims

Research Aim

To determine if prophylactic use of HFNT (for a minimum of 16 hours after tracheal extubation (inclusive of up to one hour off randomised therapy, if clinically required, see section 3.4)) is clinically- and cost-effective up to 90 days after surgery, for adult patients undergoing cardiac procedures with cardiopulmonary bypass who are at high risk of postoperative pulmonary complications.

Primary Outcomes:

- To determine if prophylactic HFNT therapy after cardiac surgery in patients at high-risk of developing pulmonary complications results in an increase in DAH90 (days alive and at home in 90 days)

- The primary objective of the health economic analysis is to estimate the incremental cost-effectiveness and cost-utility of HFNT versus standard oxygen therapy at 90 days.

Definition of primary outcome (DAH90):

'Days alive and at home' (DAH) after surgery (18, 19) is a valid and easy to measure patient-centred outcome metric. It is highly sensitive to changes in surgical risk and impact of complications and has prognostic importance. DAH accounts for major complications, prolonged hospital stay, discharge to any post-acute care nursing facility, post-discharge complications needing hospital readmission, and early death after surgery. Patients with major complications had a substantially lower DAH when compared to those without complications. DAH is considered a superior measure of quality of surgery and perioperative care over standard complication and mortality rates. It includes, and in a sense bypasses, otherwise undetected and/or unreported process of care issues and clinical outcomes. Following the approach of Myles et al (19), patients who died within 90 days of surgery were assigned a zero DAH score irrespective of whether they spent any time at home during the 90 day follow-up period. This assumption is made on the basis that the death rate in the trial population is expected to be low (around 3%, based on pilot data and registry data (10, 20), most deaths are expected to occur within the initial hospital admission (within a short time of surgery), the death rate is expected to be comparable between the two treatment arms, and it is not expected that the either treatment will impact on death rate.

Home will be defined as a person's usual abode. Home will exclude any nursing facility (rehabilitation centre or nursing home) unless this was the patient's previous residence and they return 'home' with no increase in level of care. Any hospital readmissions within 90 days of surgery are subtracted from the total. DAH90 will be calculated using mortality and hospitalisation data from the date of randomisation, which is the day of surgery (Day 0).
For example:

- If a patient dies while still in hospital, they will be assigned 0 DAH90
- If a patient is discharged from hospital on Day 6 after surgery but is subsequently readmitted for 4 days before their second hospital discharge and then returns home until 90 days post-surgery, then they will be assigned 80 DAH90.

- If a patient is discharged from hospital on Day 6 after surgery, but subsequently dies on day 89, then they will be assigned 0 DAH90. This is highly expected to be a rare outcome.

Thus, days alive and at home after surgery takes into account mortality, length of hospital stay, admission to a nursing or rehabilitation home after surgery and re-admission to hospital within 90 days of surgery. The choice of primary endpoint (DAH90) is aimed at mitigating potential sources of bias due to the unblinded nature of the trial. DAH90 depends on a number of interrelated variables measured between randomisation and 90 days post-surgery: mortality; length of hospital stay; discharge destination (previous residential status or increased care); and readmission to hospital (or to a residence with increased level of residential/nursing care).

Definition of incremental cost effectiveness

The incremental cost-effectiveness ratio (ICER) reflects the difference in costs between two interventions divided by the difference in effects (such as QALYs). The statistic is interpreted in relation to threshold values for the willingness to pay for QALYs. By presenting this statistic in association with its uncertainty and threshold values, cost-effectiveness acceptability curves can be mapped to show the probability that an intervention is cost-effective at different willingness to pay for QALY values. We are aware that treatment of these cases is not ideal for the health economic analyses. We discuss this in Section 5.1.2 and we will ensure sensitivity analysis is conducted to inform decision-making at the interim analysis on the potential impact on sample size calculations of counting each day alive and at home is included in DAH90 instead of assigning a 0.

Exploratory Secondary Outcomes:

- Health economic analysis to estimate the incremental cost-effectiveness and cost-utility of HFNT versus standard oxygen therapy at 30 days.
- Mortality
- Postoperative pulmonary complications (21)
- ICU re-admission rate
- Total length of ICU stay (days) (22)
- Total length of hospital stay (days)
- Readmission to hospital
- Incidence of stroke
- Incidence of sepsis
- Incidence of myocardial infarction
- Incidence of acute kidney injury
- Oxygenation, as measured by ROX Index (defined as Sp02/Fi02 to respiratory rate ratio) (23)
- Patient-reported outcomes (EQ- 5D-5L)
- Patient level of assistance needed with Activities of Daily Living (BARTHEL questionnaire) (24, 25)
- Quality of survival (EQ-5D-5L QALYs (26)).
- Health service and resource use

For definition of exploratory secondary outcomes see Appendix 1.

1.3 Trial Design

The trial is an adaptive, multicentre, parallel group, randomised controlled clinical trial with embedded cost-effectiveness analysis comparing the use of high-flow nasal therapy (HFNT), to standard oxygen therapy for a minimum of 16 hours (inclusive of up to one hour off randomised therapy, if clinically required, see section 3.4) after tracheal extubation, in patients at high risk of respiratory complications following cardiac surgery. Patients will be recruited over 3 years across at least 10 centres in the UK, 7 centres in Australia and 1 centre in New Zealand. Please refer to Appendix 4, for the international management and governance structure).

Figure 1. Patient Flow Diagram

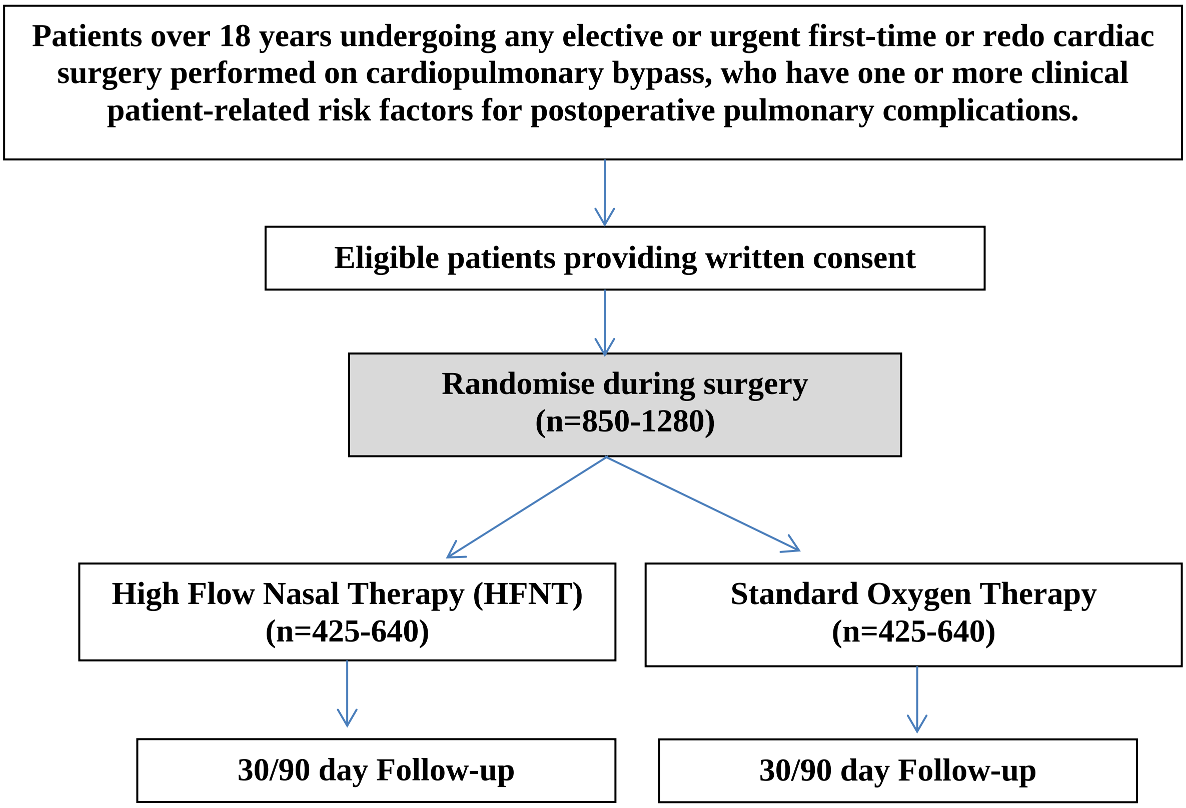

Blinding

Due to the nature of the intervention, clinical staff in ICU and on the wards cannot be blinded whilst the patient is receiving randomised therapy. However, a team of research staff at the central clinical trials unit will collect data on outcomes and these staff will be blinded. In addition, the decision to discharge patients from hospital, which affects the primary outcome, will be made by clinicians who are independent of the research team at each site, according to standard protocols (see below). The interim analysis and sample size re-estimation will be done by an independent unblinded statistician so that the statistical trial team can remain blinded until the final analysis to preserve type I error rates at a 5% value.

2 Patient Recruitment Criteria

2.1 Inclusion Criteria

1. Aged 18 years or over.
2. Undergoing any elective or urgent first-time or redo cardiac surgery performed on cardiopulmonary bypass
3. Have one or more clinical patient-related risk factor for postoperative pulmonary complications (COPD, asthma, lower respiratory tract infection in last 4 weeks as defined by use of antibiotics, body mass index ≥35 kg/m^2, current (within the last 6 weeks) heavy smokers (> 10 pack years))(27, 28).

For the purposes of the trial, the following definitions apply:

Smoking pack years = Number of packs of cigarettes smoked per day X Number of years smoked
(where 1 pack = 20 cigarettes)

N.B. If a patient does not have a >10 pack years history and/or has only been using e-cigarettes or a vape in the past 6 weeks, they should not be included in the study.

Asthma is a disease characterized by recurrent attacks of breathlessness and wheezing, and **patients will have been prescribed medication by inhalers or nebulisers** (either bronchodilators or steroids).

Chronic Obstructive Pulmonary Disease (COPD) is an umbrella term used to describe chronic lung diseases that cause limitations in lung airflow. The more familiar terms ‘chronic bronchitis’ and ‘emphysema’ are no longer used but are now included within the COPD diagnosis. The most common symptoms of COPD are breathlessness, or a ‘need for air’, excessive sputum production, and a chronic cough. **Patients suitable for the NOTACS trial will have been prescribed medication by inhalers or nebulisers** (either bronchodilators or steroids).

2.2 Exclusion Criteria

1. Requiring home oxygen therapy.
2. Deep hypothermic circulatory arrest planned.
3. Contraindication to HFNT, e.g. nasal septal defect.
4. Requirement for home respiratory support (including: CPAP, BiPAP).
5. Requiring emergency cardiac surgery defined as surgery required within 24 hours of the decision to operate.
6. Patients not fluent in English.

2.3 Coronavirus Advice

Elective cardiac surgery patients will be, by definition, COVID-19 negative. The NOTACS trial patient population is limited to elective cardiac surgery patients and in-house urgent cardiac surgery patients, both of whom undergo COVID-19 testing prior to surgery. Patients requiring emergency cardiac surgery are excluded from the protocol. The NOTACS trial recommends sites to follow their local patient pathways and procedures in regards to COVID-19 testing and personal protective equipment (PPE).

3. Visit Schedule

3.1 Schedule of Events

Visit Number	Visit 1<br>**Screening**	Visit 2<br>**Baseline**	Visit 3<br>**Randomisation**	Visit 4<br>**Discharge**	Visit 5<br>**30 Days (+7 days) Post-op**	Visit 6<br>**90 Days (+14 days) Post-op**
Time Interval of Visit	Prior to Surgical Admission (or after admission if in-house urgent)	Surgery Admission	During or After surgery & prior to extubation	Day of Discharge (+30 days for questionnaires)	30 days (+7 days) Post-op	90 days (+14 days) Post-op
**Activity**						
Inclusion/Exclusion Criteria	**X**					
Informed Consent		**X**				
Demographics		**X**				
Past Medical History		**X**				
EuroSCORE II & ARISCAT Risk Assessments		**X**				
EQ-5D-5L & BARTHEL Questionnaires		**X**		**X**	**X**	**X**
Participant & Family Resource Use Questionnaires		**X**		**X**	**X**	**X**
Adverse & Serious Adverse Events Assessed (from the point of extubation			**X---X**			
Inpatient Medication Log (to start from the point of extubation)			**X----------------------X**			
Inpatient Location Log (to start from the point of extubation)			**X----------------------X**			
Inpatient Oxygen Therapy Log (to start from the point of extubation)			**X----------------------X**			
Participant Location and Medication Diary				**X-------------------------------------X**		
Randomisation/ Initiation of HFNT or Standard Oxygen Therapy			**X**			
ROX Index			**X----------------------X**			
Record of Respiratory Support Escalation			**X----------------------X**			
Record of Post-operative Complications			**X----------------------X**			
Record of Intensive care Length of stay and Re-admissions				**X**		
Record of Hospital Discharge Destination				**X**		
Record of Hospital Length of Stay				**X**		

3.2 Screening (Visit 1)

Patients scheduled for elective or urgent first-time or redo cardiac surgery (coronary artery bypass grafting (CABG), valve surgery or both) except if deep-hypothermic circulatory arrest required, will be screened for eligibility. Eligible patients undergoing elective surgery will be identified from lists of those accepted for surgery by members of the research team. Those meeting all eligibility criteria will be given or a patient information sheet and an invitation letter and then either approached by telephone or face to face and informed about the trial prior to admission. Urgent patients requiring surgery that have been admitted to hospital and are awaiting surgery will be given a patient information sheet and patient invite letter during admission and then approached to participate within the trial prior to surgery.

3.3 Baseline (Visit 2)

Written informed consent will be obtained by a member of the trial team at the patient's baseline visit, after the patient has had ample time to read the information sheet, consider the trial and ask any questions. A member of the research team will explain to each patient the nature of the trial, its purpose, the procedures involved, the expected duration, the potential risks and benefits involved and any discomfort it may entail (*Ref. International Conference of Harmonisation of Good Clinical Practice (ICH/GCP)5 4.8.7).* The research team must also ensure that the patient is aware and consents for their personal details such as name, date of birth, address, email address, NHS number, GP name and address to be transferred to Royal Papworth Hospital NHS Foundation Trust via an encrypted database so that the central clinical trials unit staff can complete blinded follow-ups. The follow-up options should also be discussed with the patient and indicated on the consent form (i.e., if they wish to complete follow-up via an online system (OpenClinica Participate™) or by telephone) and the patient should be reminded that follow-ups may take up to 45mins of their time. Patients should also be made aware that if no online response to the follow-up has been made within 4 days of the due date then a member of the central team will telephone them.

The ultimate responsibility for obtaining written informed consent lies with the Investigator but this responsibility may be delegated to a suitably trained and experienced person. Prior to the patient's participation in the trial, the written informed consent form must be signed and personally dated by the patient and by the team member who conducted the informed consent discussion (*Ref. ICH/GCP 4.8.8*). Each box at the end of each statement on the consent form must be initialled by the patient. Each patient must be informed that participation in the trial is voluntary and that he/she may withdraw from the trial at any time and that withdrawal of consent will not affect his/her subsequent medical treatment (*Ref. ICH/GCP 4.8.10*). A copy of the informed consent document will be given to the patient for their reference (*Ref. ICH/GCP 4.8.11*). One copy will be filed in the patient's medical record and the original filed in the Site File. The patient must not have any trial specific procedures prior to giving informed consent. Once this has been completed, baseline EQ-5D-5L, BARTHEL and health service and resource use questionnaires can then be completed with the patient. Data to calculate EuroSCORE II and ARISCAT score will also to be collected.

In the event of surgery for a consented patient being cancelled and rescheduled, local sites should conduct the following:

- Surgery rescheduled to be performed within four weeks of the original date: the Baseline CRF and questionnaires do not need to be repeated however staff should check that the data collected is still correct.
- Surgery rescheduled to be performed more than four weeks from the original date: the Baseline CRF and questionnaires should be repeated.

3.4 Randomisation (Visit 3)

This is a pragmatic trial so that perioperative management (anaesthetic technique, surgical procedure, intra-operative mechanical ventilation strategy, and postoperative invasive mechanical ventilation weaning strategy) will not be affected by patients' participation in the trial and will be conducted according to usual local practice. Once surgery has finished, patients will be transferred to the post-surgery recovery unit or ICU as per standard clinical practice.

Randomisation will be performed while the patient is undergoing surgery, or postoperatively in the ICU prior to extubation. By randomising at this late stage we hope to limit the impact of cancelled or delayed surgeries and reduce the need to replace or re-randomise patients. Patients will be randomly assigned to receive either HFNT or standard oxygen therapy in a 1:1 allocation ratio using an online tool (provided by Sealed Envelope). Randomisation will be stratified by centre. Random permuted blocks within strata will be used to reduce predictability of the randomisation sequence.

After cardiac surgery, patients will be transferred sedated and with their trachea intubated to the post-surgical recovery area. This may be an Intensive Care Unit, High Dependency Unit or specific Recovery Unit as per local practice. Once patients fulfil the standard agreed protocol [minimal bleeding via chest drains; temperature > 36°C; stable cardiovascular function; neuromuscular block worn off or reversed; sedation stopped; patients responsive to command and successful trial without mechanical ventilation (defined as saturations > 93% with inspired oxygen less than or equal to 60%)] they will then be extubated according to the agreed Trial Extubation Protocol (see Appendix 2) and will receive either HFNT or standard oxygen therapy for a minimum of 16 hours according to their randomised allocation. During the 16 hours, up to a total of one hour off treatment is allowed for any required transfers around the hospital and/or physio mobilisation. Patients will be transferred to the surgical ward as per local practice and will be assessed at least every 24 hours as per local practice – if sats > 93% on air and RR < 20, then HFNT or standard oxygen will be discontinued. If sats < 93% or RR > 20, then HFNT or standard oxygen will be continued for a further 24 hours then the patient will be re-assessed every 24 hours. If a patient deteriorates during HFNT or standard oxygen therapy, then the agreed Trial Escalation of Respiratory Therapy Protocol (see Appendix 3) will be followed.

3.5 Discharge (Visit 4)

Patients will be discharged from hospital as per local guidelines. The EQ-5D-5L, BARTHEL questionnaires and health service and resource use questionnaire will be completed at discharge. A member of the research team will also provide a short explanation of how to complete the participant location and medication diary at home over the following 90 days post-operative. Patients will also be reminded of the follow-up completion method they chose at the baseline visit and the online system (OpenClinica Participate™) will be demonstrated to those participants in the UK who chose to use it. A member of the research team is able to complete the quality of life and health service and resource use questionnaires over the telephone if a patient is discharged unexpectedly, up to 30 days post discharge. The participant location and medication diary should be sent to the patient with the patient discharge letter accompanying it. Discharge data should then be collected including, ROX index (as defined in section 4.3.4) should be calculated.

Note: The discharge CRF should be completed on day of discharge regardless if this occurs after 30 day follow-up. If a patient is discharged before the research team are able to complete the Discharge questionnaires with the patient, site staff are asked to attempt to contact the patient to complete them over the phone for up to +30 days post-discharge.

3.6 Post-Discharge (Visits 5 & 6)

Primary outcome data will be collected using a paper based participant location and medication diary in which patients will be asked to document when they change location and the medications they are taking at 30 and 90 days post-surgery. All patients (including those who have chosen online follow-up), will be called by the central clinical trials unit staff at 7 days post discharge to resolve any problems that have arisen in completion of the paper participant location and medication diary. Patients who have chosen telephone follow-up are then contacted at 30 (+7 days) and 90 days (+ 14 days) post-surgery to collect outcome data and complete questionnaires. Participants that are uncontactable at 30 days and 90 days post-surgery will be called daily up to +7 days and +14 days respectively, post day of follow-up to complete telephone follow-up. This reduces to twice a week up to day 30 + 30 days and day 90 + 90 days, with a final attempt on the day 30 + 30 and day 90 + 90, respectively. If participants are uncontactable following the final attempt, their GP will be contacted to obtain the primary outcome data. In the event that a patient becomes distressed during telephone follow-up, central study team member will follow the telephone interview escalation protocol (appendix 5).

At the 30 and 90 day follow-ups, telephone patients will be asked to relay diary data to the central trials team member. Patients who have chosen online follow up will be asked to confirm they are completing the paper participant location and medication diary and to highlight if they are having any issuing in completing the diary. Once a patient has completed their diary at 90 days they will receive a letter enclosing a pre-paid envelope to return their diary to the central trials team. If the central trials team do not receive the participant location and medication diary, they may call the patient after the 90 day point to retrieve this data.

Patients will have the option to complete quality of life questionnaires and health service and resource use questionnaires either online (OpenClinica Participate™) or over the telephone with a member of the central trials team. If a patient is unable to complete the questionnaires via the telephone or online, then the central trials team should send the quality of life and health service and resource use questionnaires to the patient with the relevant accompanying letter as a last resort. An escalation process to ensure patients complete online questionnaires can be seen in Figure 2. GPs, their receptionists or other medical facilities will be contacted by the central clinical trials unit staff in case of difficulty contacting patients or to gain further information on any adverse or serious adverse events including gaining information regarding hospital admissions and use of primary care services. Patients will normally attend back to the hospital for surgical follow-up at 6-8 weeks independently of the trial as per local guidelines.

Note: In the event that a participant is still an inpatient at 30 day follow-up, site staff are asked to complete the 30 day CRF’s and questionnaires and scan a completed copy to the central clinical trials unit staff. In the event of a participant being an inpatient at 90 day follow-up, site staff should complete the discharge CRF’s and questionnaires at the 90 day time point. Site staff should ensure that the participant’s inpatient logs continue to be updated until the patient has been discharged or up to the 90 day time point, whichever is first.

Figure 2. Follow-up Flow Diagram

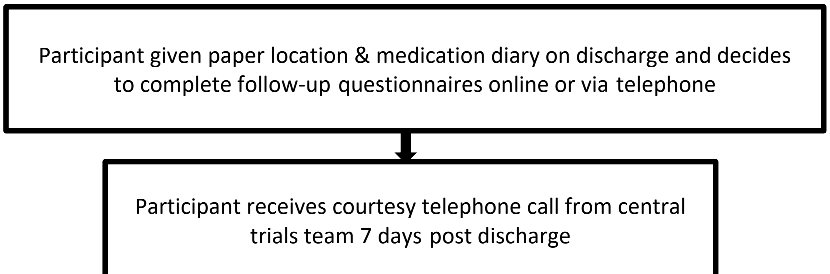

Participants will be called daily up to +14 days post day of follow-up to complete telephone follow-up. This reduces to twice a week up to day 90 + 90 days, with a final attempt on day 90 + 90. If participants are uncontactable following the final attempt, their GP will be contacted to obtain the primary outcome data.

*Note: If a patient is unable to complete quality of life and health service and resource use questionnaires over telephone or online then they will be sent out to patients with an accompanying letter.

3.7 Withdrawal From Trial

Patients are free to withdraw from the trial at any time. If a patient withdraws their consent, they will be withdrawn from the trial. However we will request that any data collected up to the point of withdrawal is retained for analysis. This will be explicitly requested on the trial consent form. A withdrawal form will need to be completed in the online database.

Any patients who withdraw consent before randomisation will be replaced. Patients who withdraw consent after randomisation will not be replaced.

3.8 Strategies to improve adherence to intervention

Routine standard of care is encouraged during all aspects of the trial. However patients are to receive at least 16hrs of randomised therapy with a total of one hour off treatment allowed for any required transfers around the hospital and/or physio mobilisation. The trial asks for the protocol to be followed for tracheal extubation at which randomised therapy is initiated, and also for escalation of care if there is any deterioration in respiratory function after tracheal extubation.

All trial staff, both clinical and non-clinical, will receive protocol and device training (if required) before being entered onto the delegation and training logs to ensure protocol adherence. Clinically, protocols will be laminated and placed by the trial team at each participant's bedside when the patient is admitted to the post-surgery care area and the randomised therapy will be set up by the bedside nurse so that it is ready for when the patient's trachea is extubated. This is to ensure all clinical members of staff are aware of the trial protocol.

4. Data Collection Methods

Papworth Trials Unit Collaboration (PTUC) Data Management team will provide data management oversight for the trial and will coordinate with the Statistical and Health Economics teams to ensure that all trial data is ready for analysis.

Data will be collected using bespoke trial case report forms and a patient diary designed in collaboration with the Trial Statistician, Trial Data Manager and Health Economist to ensure that all variables are accurately recorded. Data will be then transcribed on to a purpose-designed data management system, OpenClinica, with blinded and unblinded access.

Trial sites will have unblinded access to this system to collect demographic and surgical data to answer trial outcomes. Consent to the trial will allow patient identifiable data to be sent to the central clinical trials unit via OpenClinica, which is an encrypted database where follow ups will be completed by blinded members of central clinical trials unit staff without bias.

Following PPI feedback from our pilot trial, patients will also be given the opportunity to complete trial follow-ups online, with text and email reminders when these are due. The central clinical trials unit staff will ensure online forms have been completed using the escalation process in Figure 2 (above). Patients will be telephoned if online forms have not been completed within 4 days of the due date.

4.1 Baseline Data

Baseline data will be collected following consent. This will include basic demographic data (age, sex, residential status), past medical history as well as quality of life (EQ-5D-5L), activity of daily living (BARTHEL) and health service and resource use questionnaires. Standard of care data will be collected to calculate the EuroSCORE II, which predicts mortality at 30 days after surgery. Data will also be collected to calculate the ARISCAT score, which predicts the risk of in hospital pulmonary complications after surgery, including respiratory failure. Data relating to antibiotic, cardiac and respiratory medication use will also be collected.

4.2 Primary Outcome Data Collection (DAH90)

Primary data collection will be collected using a paper patient diary (Participant Location and Medication Diary) which patients will be asked to complete for 90 days postoperatively. Patients will be asked to record every time they change living location and the date they moved. Locations will be pre-coded and defined as: Home; Hospital; Residential Home; Nursing Care; Relative's Home; or Other. Incomplete diaries completed by patients will be completed by calling the patient's GP surgery and using hospital discharge summaries for dates of change of living location. Further information regarding death or any additional hospital admission will be collected from the discharge summary and will be cross-referenced with GP records.

4.3 Exploratory Secondary Outcomes

Patient-reported outcomes (EQ-5D-5L), patient level of assistance needed (BARTHEL Activities of Daily Living) and health service and resource use questionnaires will be used to collect exploratory secondary outcome data post-operatively completed by local site staff at discharge (+30 days) and central clinical trials unit staff at 30 (+7 days) and 90 days (+14 days) after surgery (randomisation).

4.3.1 EQ-5D-5L

This is the most frequently used, generic, preference-based instrument for measuring the health utilities of patients in economic evaluations. It is recommended for health technology assessment by the National Institute for Health and Clinical Excellence (NICE)(29). The EQ-5D-5L descriptive system comprises the following five dimensions: mobility, self-care, usual activities, pain/discomfort and anxiety/depression. Each dimension has 5 levels: no problems, slight problems, moderate problems, severe problems and unable to complete. The patient is asked to indicate his/her health state by ticking the box next to the most appropriate statement in each of the five dimensions. This decision results into a 1- digit number that expresses the level selected for that dimension. The digits for the five dimensions can be combined into a 5-digit number that describes the patient's health state (30). The recent notice (May 2019) by EuroQol group has been acknowledged and the ongoing work on valuation sets of England has been noted. Therefore, if NICE does not change their position statement, the van Hout et al cross-walk to the EQ-5D-3L alongside the potential use of other values produced in sensitivity analyses will be completed. This will ensure the trial complies with future expectation.

4.3.2 BARTHEL Activities of Daily Living

The Barthel Index covers ten domains: feeding, bathing, grooming, dressing, bowels, bladder, toilet use, transfers, mobility and stairs. Performance on these domains is rated by level of assistance required.

4.3.3 Health Service & Resource Use

Bespoke health service and resource use logs will be completed by the research team using patient records to collect inpatient stay data: e.g. surgery completed, time in theatre and ICU by hours (including returns), types and numbers of tests, procedures, medications, types and treatment for complications, post index-hospital discharge care (days in any hospital, residential care by type), A&E and OPD visits and use of primary care services (e.g.GP/nurse/physio visits, home visits). Oxygen therapy use will be logged by the research team and will include details such as method of oxygen delivery, settings ie number of litres/min and date and time method was initiated and stopped. Research staff will also log antibiotic, cardiac and respiratory medication use as well as in-hospital patient location to include length of stay in each location within hospital eg. ITU, HDU and ward setting. Bespoke patient health service and resource use questionnaires on costs borne by patients and families will include: out of pocket expenses for residential care and assisted living care, care-related expenditure on travel, equipment and prescriptions, and days of unpaid family care (specifying whether this includes days off work).

4.3.4 ROX Index

ROX Index can be used to predict HFNT outcome. Data to calculate ROX Index will be collected at 2, 6, 12, 24 and 48 hours post extubation.

ROX Index = Sp02/Fi02 to respiratory rate ratio

4.4 Case Report Form Completion

The Investigator should ensure the accuracy, completeness, legibility and timeliness of the data recorded in the case report forms (CRFs) and in all required reports to e.g. the Sponsor, Funder, R&D, REC. The Sponsor will provide participating sites with a NOTACS trial specific data entry guide to provide instructions on using the database.

4.4.1 Source Documentation

The investigator/clinical research team must maintain source documents (patient's medical record) for each patient in the trial, consisting of all demographic and medical information. A copy of the consent form and patient information sheet will also be filed in the patient's medical record. All information in the CRFs, apart from the questionnaires, must be traceable to and consistent with the source documents in the patient's hospital case notes (Ref. ICH/GCP 4.9.2).

4.4.2 Errors and Corrections

A robust audit trail within OpenClinica tracks all changes to the data and retains a history for each variable, including old and new value, date and time of the change and which user made it. Errors made on any paper CRF's should be striked through with a single line, dated and signed against.

4.4.3 Retention of Documents

All trial documentation should be stored for 15 years after the last patient has completed their last visit.

5. Data Analysis

5.1 Sample Size

Results from the pilot study (10) and information provided by collaborative hospitals were used to derive the required sample size for the NOTACS trial. The sample size calculation relied on several parameters that were provided from the pilot study and may differ between sites in the multicentre design; because of this uncertainty the NOTACS trial includes an interim sample size re-estimation, a type of adaptive design. This will provide protection against important deviations from the original sample size assumptions. The minimum target sample size (based on original assumptions) is 850 randomised participants. The adaptive design will allow for a maximum sample size increase to 1280 patients.

5.1.1 Initial Sample Size Calculation

The primary endpoint (DAH90) typically has a left-skewed bi-modal distribution with a small spike at 0 due to deaths. Following the approach of Myles et al (19), patients who die within 90 days of surgery will be assigned a zero DAH score irrespective of whether they spent any time at home during the 90

day follow-up period. This assumption is made on the basis that the death rate in the trial population is expected to be low (around 3%, based on pilot data and registry data (10,20)), most deaths are expected to occur within the initial hospital admission (within a short time of surgery), the death rate is expected to be comparable between the two treatment arms, and it is not expected that the either treatment will impact on death rate. The required sample size was obtained by simulations (100000 replicates) by first generating length of stay (LOS) using a lognormal distribution. Based on the information provided by collaborative hospitals, the parameters of the lognormal distributions in both arms were derived through a pooled weighted average. The variability was calibrated to SD =12.85 in the control arm and SD=3.20 in the treatment arm. The median LOS in the control arm was set to 8 days. We assumed a 3% death rate (based on pilot data and registry data (10, 20), and following the approach of Myles (19) we treated any death within the 90 day follow-up period as scoring 90 for LOS regardless of when the death occurred. LOS was truncated at 90 days (the maximum for our follow-up period).Finally DAH90 was computed as 90 minus LOS. The resulting data are bimodal with a spike at 0, as seen with observed data of this type.

A total sample size of n=310 has 90% power to detect an increase of 2 days in the median DAH90 using the Mann-Whitney-Wilcoxen test for the analysis. After adjustment for 12% crossover from standard oxygen to HFNT and 25% crossover from HFNT to standard oxygen as well as an extra 5% loss to follow up (equally distributed among arms), the total sample size needed to detect a 2-day increase with 90% power with an intention to treat analysis is 850 patients. Therefore, in the first instance the trial aims to recruit 850 patients.

5.1.2 Adaptive Design

The assumptions used for the original sample size calculation were based on pilot data (10) and data provided by the largest participating centres. As NOTACS is a multicentre trial, using a different primary endpoint to the pilot data, we found that the sample size calculation was very sensitive to the standard deviation, level of treatment switches and loss to follow up assumed. NOTACS has been designed as an adaptive trial with an interim sample size re-estimation planned after 300 patients complete 90 days post-randomisation follow-up.

At the interim sample size re-estimation, we will use the data accumulated so far to re-estimate a number of “nuisance” parameters including:

- standard deviation of DAH90 in the standard-oxygen therapy arm
- standard deviation of DAH90 in the HFNT arm
- treatment switch rate from standard oxygen therapy to HFNT
- treatment switch rate from HFNT to standard oxygen therapy
- drop-out rate
- death rate

Treatment efficacy will not be assessed at the interim analysis. Sensitivity analysis will also evaluate the impact of accounting for all days alive and at home (ie even if patients die before 90 days) on sample size estimation. This will facilitate discussion of the impact of this assumption on the final estimation of DAH90 and QALYs. These analyses will inform how final analyses for the effectiveness and health economics can be aligned in terms of the primary endpoint definition and used to better address the co-primary questions. Further details of these analysis and potential simulations will be provided in the SAP/HEAP.

After the interim analysis, the sample size of the trial will be updated with a maximum increase up to 1280 patients. There are several possible outcomes from the sample size re-estimation which are summarised in table 2.

Table 2. Recommended sample size from interim sample size re-estimation and course of action

Recommended sample size from interim sample size re-estimation	Course of action
≤850	Continue recruitment to 850

851-1280	Continue recruitment to the new recommended sample size
>1280	Continue recruitment to 1280

The sample size re-estimation will be done using an independent statistician to allow the trial statisticians to remain blinded, in order to preserve the type 1 error rate at 5%. This sample size adaptation may prevent an underpowered trial if moderate deviations from the assumptions made for the initial sample size calculation are observed.

5.1.3 Interim Analysis

Details of the outcome of the interim sample size re-estimation planned after 300 patients complete 90 days post-randomisation follow-up are provided in Appendix 6.

5.2 Recruitment and Retention

In addition to an internal pilot phase provided by the sample size re-estimation included in the adaptive design, an internal one month pilot phase in each trial centre to enhance the efficiency and internal validity of the main trial will be used. This will focus largely on recruitment, randomisation, intervention and follow-up assessments.

The potential timings of reaching the specific sample sizes were in estimation via the initial predictive model of patient recruitment number (32). According to the prediction, the target sample size (n = 850) will be attainable by 37 months. The sample size of 300 for the interim sample size re-estimation will be achievable around 20 months after recruitment commences. The maximum sample size of 1280 is feasible at the end of the extended recruitment period of the trial (by 57 months). Furthermore, there will be at least two points in the trial for trial statisticians to aid further discussion on recruitment performance with predictions: at ten months after recruitment commences to predict whether the initial interim is attainable and again, immediately after the interim to foresee whether the revisited target is achievable in the time. Prediction methods, such as models in (32), regressions models and so on, will be referred to use for the tasks.

The recruitment monitoring reference has the primary basis on the lower bound of the predicted recruited number. The trial team will monitor recruitment performance and prepare for necessary actions. There will be a formal assessment of recruitment at Month 15 after 168 patients have been enrolled. The Trial Steering Committee will hold a meeting to assess recruitment against monthly targets and if the recruited figure shows a significant drop below the adjusted lower bound, measures including addition of further sites will be introduced that aim at boosting recruitment.

It is expected that each centre will recruit a minimum number of patients per month over each 3 month period to get a ‘green light’ to continue in the trial. If this minimum target is not met, the site will go ‘amber’ and be given another 3 months to meet minimum recruitment, then if this is not met the site will go ‘red’ and if recruitment cannot be improved in a further 3 months the site will be removed from the trial and another site activated. The Sponsor, CTU and Local CRN Research Delivery teams will give sites labelled amber or red all available assistance to improve recruitment.

5.3 Statistical Analysis

The primary outcome is Days alive and at home within the first 90 days after surgery (DAH90). Home will be defined as a person’s usual abode or that of a close relative. Home will exclude any nursing facility (rehabilitation centre or nursing home) unless this was the patient’s previous residence and they return ‘home’ with no increase in level of care. Any hospital readmissions within 90 days of surgery are subtracted from the total. Any patients who die within 90 days of surgery will be assigned a DAH score of zero. This assumption is made on the basis that the death rate in the trial population is expected to be low (around 3%, based on pilot data and registry data (10,20)), most deaths are expected to occur within the initial hospital admission (within a short time of surgery), the death rate is expected to be comparable between the two treatment arms, and it is not expected that the either treatment will impact on death rate. DAH90 will be calculated using mortality and hospitalisation data from the date of surgery (randomisation) (Day 0).

The primary outcome of days alive and at home up to 90 days (DAH90) will be analysed at the end of the trial using the Mann-Whitney-Wilcoxon test. This will be the primary analysis. Note that the reason for using such test is because the sampling distribution of DAH90 was found to be skewed. Contrasts for the primary outcome will be used to evaluate the difference in the median DAH90 between the two treatment arms at a 5% significant level. 95% confidence intervals giving a range of plausible effects will be reported. The primary analysis will be unadjusted for baseline variables.

The primary analysis will be on the basis of ™TT. The effects of adherence, attrition, and likely sources of bias on the primary effect estimate will be evaluated using per protocol, safety and sensitivity analyses. In particular, we will perform a sensitivity analysis for the primary end-point to assess the impact of assigning a DAH score of zero to patients that die at any time within the 90 day follow-up period by relaxing this rule and replacing these zero values by the observed DAH value for these patients.

It is expected that the secondary outcome of days at home up to 30 days (DAH30) will have similar distributional characteristics to that of DAH90. Hence, the Mann-Whitney-Wilcoxon test will also be used to evaluate whether there is a statistically significant difference in the median DAH30 between the two treatment arms.

Secondary analyses will be performed to allow adjustments for baseline variables (such as EuroSCORE II or ARISCAT score) to be made.

Other sensitivity analyses will be performed in order to evaluate the robustness of the primary analysis. ‘Intention to treat’ and ‘per protocol’ analyses will be reported and the extent of bias on the estimates will be discussed at the final analysis stage.

The statistical analysis will be reported according to CONSORT extension guidelines for adaptive trials (33). In cases of missing data, the missing data mechanism will be explored, and multiple imputation may be applied as a sensitivity analysis as appropriate. However, from the pilot study a high missing data rate it is not expected

5.3.1 Methods in analysis to handle protocol non-adherence and any statistical methods to handle missing data

‘Intention to treat’ and ‘per protocol’ analyses will be reported and the extent of bias on the estimates will be discussed at the final analysis stage. In cases of missing data, the missing data mechanism will be explored, and multiple imputation may be applied as a sensitivity analysis as appropriate. However, from the pilot study a high missing data rate it is not expected.

5.4 Health Economics Analysis

The economic evaluation focuses most data collection on the initial in-patient stay, followed by costing of health and social care service use in the follow up period. This ensures the detail for expected (e.g. LOS and ICU use) and any unexpected change (e.g. treatment of complications) from the surgical stay is collected and any implications for shifting care to other hospitals/residential care or to patients is captured in broader detail.

The base case economic evaluation will adopt an NHS view point, with a public sector and patient/family viewpoints included in sensitivity analysis. Intervention costs including set up (e.g. training), initial inpatient care (e.g. length of stay in ward/theatre/ICU including all readmissions, oxygen use by type, treatment of complications, procedures, tests, medication) and follow-up care costs (e.g. readmission to hospital, use of A&E services, appointments and home visits for primary care, use of other community care services, days in residential care by type, medication) to 90 days will be compared with the usual care control. Following discussion with PPI representatives, patient cost data will focus on their largest elements; out of pocket expenses for residential care and assisted living, plus days of unpaid caring by family members. Health service and resource use data will be collected from patients and routine sources. Unit costs will be valued using national costs (34), where

available, and literature or local costs where not. Outcomes to be used in the economic analysis will include the primary outcome DHA90 and EQ5D5L quality adjusted life years (QALYs) with the 5L version selected following with the recent NICE statement (35).

Descriptive analysis will provide total and average costs and outcomes, and cost profiles by arm. Regression-based analyses of costs and outcomes will account for missingness, censoring, skewness, and correlation between costs and outcomes. The effect of baseline characteristics (e.g. EuroSCORE II for surgical risk, ARISCAT score for risk of in-hospital post-operative pulmonary complications, gender, age, baseline quality of life, residential status) and any imbalance in covariates on costs and outcomes will be evaluated. Bootstrapping will be used to reflect uncertainty in the incremental cost-effectiveness ratios and correlations between costs/effects. Results will be presented as incremental cost-effectiveness ratios, cost-effectiveness acceptability curves and net benefit statistics, in accordance with good practice guidance and recommendations by NICE (36, 37, 38). Sensitivity analyses will include viewpoint, alternative methods for dealing with missingness, and any assumptions needed for valuation. A health economic analysis plan will be submitted to the TSC in collaboration with the statistical analysis plan.

6. Adverse & Serious Events

The definition of an adverse event is: 'Any untoward medical occurrence in a patient which does not necessarily have a causal relationship with the trial intervention'. This includes 'any unfavourable and unintended sign (including an abnormal laboratory finding), symptom or disease temporally associated with the trial intervention'. This may include, for example, a common seasonal cold or an accident.

The definition of a serious adverse event (SAE) is one that fulfils at least one of the following criteria:

- Is fatal- results in death
- Is life threatening
- Requires inpatient hospitalisation or prolongation of existing hospitalisation
- Results in persistent or significant disability/incapacity

The definition of a suspected unexpected serious adverse reaction (SUSAR) is a serious adverse event that is thought to be possibly or definitely related to the device and is unexpected (i.e., not listed in the protocol as an expected occurrence).

6.1 Recording and Reporting

In cardiac surgery, post-operative complications are common. Only device-related adverse events (i.e., adverse reactions) and SAEs that are device-related and/or 'unexpected' will be reportable to the Sponsor. Details of these events should be sent to the Sponsor within 24 hours of becoming aware of the event. Elective non-cardiac surgery, or any other intervention or treatment during the follow-up period but scheduled before a patient is recruited to the trial is not classed as an unexpected SAE.

Figure 3. AE & SAE Flow Diagram

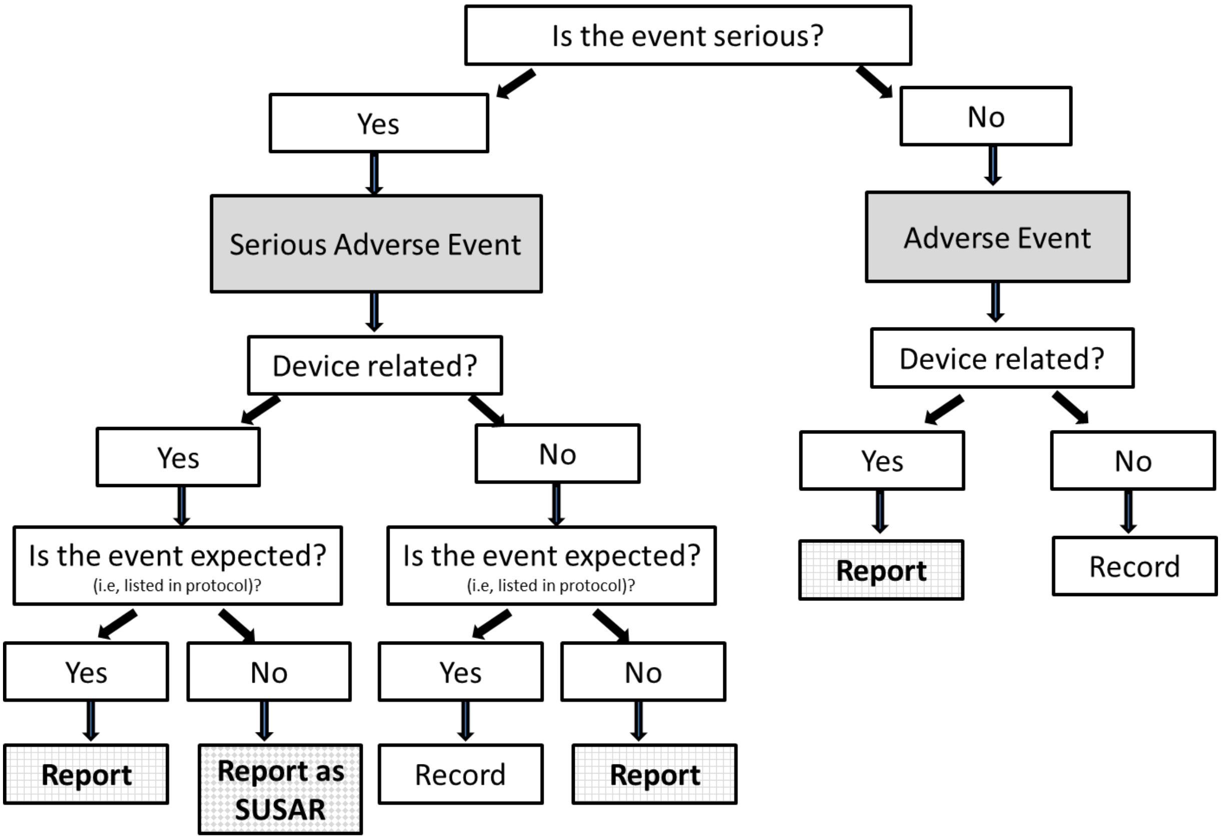

Adverse events and serious adverse events will be collected from the time of tracheal extubation to discharge. From discharge up to 90 days after surgery only SAEs will be collected.

For events collected from tracheal extubation to discharge, the local PI will conduct medical assessment including the causality of the event. The Sponsor also delegates responsibility for ‘expectedness’ assessment to the local PI. Unexpected events are those not listed in the trial protocol.

Safety follow-up from discharge up to 90 days after surgery will be conducted by the central clinical trials unit staff who are blinded. Medical assessment (including causality and expectedness assessment) of SAEs will be conducted by the CI. The central clinical trials unit staff will inform the relevant local site of the SAE via email attaching a copy of the completed SAE form. Consent from participants will also be obtained for the transfer of any additional documents gathered during the course of safety follow-up for example GP Summaries and Discharge Letters and reports from third party healthcare providers. As per GCP guidance, local sites are required to document all SAEs collected within the patient’s medical notes.

Non-reportable events will be recorded on the trial CRFs via the online database. Any events that are reportable to the Sponsor should be recorded via the online database and will be reported to the Sponsor for Sponsor Assessment automatically via the online database. SUSARs will be reported to REC, the Data Monitoring and Ethics Committee and PIs at all participating sites.

In order to retain blinding to the treatment allocation, AEs and SAEs collected from tracheal extubation to discharge will be classed as unblinded and completed within the unblinded database.

SAE’s collected from discharge to 90 days after surgery will be classed as blinded and completed within the blinded database.
Any AEs or SAEs that remain ongoing at the time of a patient’s last visit will be reviewed and a clinical decision made to determine whether the event can be closed at that time point, or whether the AE/SAE must be followed up until resolution.

All adverse events and serious adverse events will be coded using MedDRA version 23.1. Each event will be coded using the MedDRA Hierarchy with a corresponding Preferred Term, High Level Term, High Level Group Term and System Organ Class. All the expected AEs/SAEs were pre-coded where possible, and reviewed by a medical professional. The AE’s/SAE’s will be coded as an ongoing process, with the coding staff communicating with the site or clinical staff as necessary for clarification.

6.2 Expected Adverse Events

The below table of adverse events are ‘expected’ and should be recorded but not ‘reported’ unless causality is device-related:

Table 3. Table of Expected Adverse Events

	**Further Details (where applicable)**
Acidosis	
Arrhythmias	Including:<br>-Supraventricular tachycardia or atrial fibrillation requiring treatment<br>-VF/VT requiring intervention<br>-Bradycardia
Aspiration of stomach contents	
Bleeding	Requiring:<br>-Transfusion<br>-Return to theatre
Escalation of respiratory support	For example: (unplanned BIPAP/CPAP/re-intubation and invasive ventilation)
GI complications	Including:<br>-Peptic ulcer/GI bleed/perforation<br>-Pancreatic (amylase >1500iu)<br>-Other (e.g. laparotomy, obstruction)
Haemodynamic support,	Including use of:<br>-Any inotropes over what is used in routine practice<br>-Intra-aortic balloon pump (IABP)<br>-Need for invasive monitoring e.g Pulmonary artery catheter<br>-Vasodilator
Heart Failure	
Infective complications	Including:<br>-Wound infection<br>-Respiratory infection<br>-Sepsis
Low cardiac output	Requiring management with:<br>-Swan-Ganz catheter<br>-IABP<br>-Left ventricular assist device
Mediastinitis	Including:<br>- requiring reoperation
Neurological complications	Including:<br>-Delirium<br>-Stroke<br>-Transient ischaemic attack (TIA)
Pain in sternal wound/legs/arms incision	

sites	
Pericardial effusion	
Pnemothorax	
Pulmonary complications	Including:<br>-Re-intubation and ventilation<br>-Tracheostomy<br>-Initiation of mask CPAP ventilation after weaning from ventilation<br>-ARDS<br>-Pleural Effusion
Re-admission to ICU	
Renal complications	Including:<br>-New haemofiltration/dialysis<br>-Acute kidney injury
Resternotomy	
Thromboembolic complications	Including:<br>-Deep vein thrombosis<br>-Pulmonary embolus
Wound dehiscence requiring rewiring or treatment	

7. Management and Governance

7.1 Sponsorship

Royal Papworth Hospital NHS Foundation Trust has assumed the responsibility of Sponsor. The respective responsibilities of the Sponsor, Investigator and Trial Manager will be identified and delegated at the start of the trial.

7.2 Project Management

PTUC, a fully accredited UKCRC Clinical Trials Unit, will oversee the trial and provide project management oversight, trial management, data management, statistical and health economic analysis and research governance support as well as input into the overall trialdesign, statistical and health economic design. PTUC is experienced in managing multi-center surgical studies (including successful HTA-funded trials).

The Clinical Project Manager (CPM) and the Trial Manager ™ (who are based at Royal Papworth Hospital NHS Foundation Trust), will work directly with the other sites to co-ordinate all aspects of the trial and ensure that the trial is conducted according to ICH-GCP standards. The Clinical Project Manager will oversee the trial and manage the finances. The Trial Manager will co-ordinate all trial related activities across the participating sites, monitor progress against the project milestones, ensure full engagement with PPI and manage Research Governance activities at all the participating sites.

Regular teleconference project team meetings will be held with co-applicants and representatives from each site to deal proactively with any trial related issues as and when they occur.

7.3 Trial Steering Committee (TSC):

A Trial Steering Committee will be led by an Independent Chair. Per NIHR HTA guidelines, the TSC will be composed of an independent Statistician, Health Economist and Clinician, plus a patient representative and observers, including a representative of the Sponsor and a representative from the Research Network if appropriate and requested by the HTA.

The TSC will meet annually (or more frequently if necessary) to monitor and supervise the trial, to ensure it is being conducted according to the protocol and timelines, to review any relevant information from other sources (e.g. other related trials) and to consider recommendations made by the Data Monitoring and Ethics Committee (DMEC).

7.4 Data Monitoring and Ethics Committee (DMEC):

A Data Monitoring and Ethics Committee will be led by an Independent Chair who is an expert in the field. Per NIHR HTA guidelines the DMEC will be composed of an independent expert Statistician and Clinician.

Annual DMEC meetings will review progress against the agreed milestones, recruitment and safety. The independent DMEC will: (1) review the assumptions underlying the sample size calculations and determine whether additional interim analyses of trial data should be undertaken; (2) develop clear, robust safety stopping rules based on regular (at least yearly) adverse event monitoring; (3) consider results of other interim analyses and relevant information arising elsewhere; (4) consider any requests for the release of interim trial data and advise the trial steering committee on this; and (5) make recommendations to the trial steering committee about continuation of recruitment. An independent unblinded statistician will provide the interim reports for the DMEC.

7.5 Monitoring and Audit

Monitoring will be remote for each site with triggered on-site monitoring if required. More detail can be found in the Trial Monitoring Plan.

Quality Control will be performed according to Papworth Trials Unit Collaboration internal procedures. The trial may be subject to inspection and audit by Royal Papworth Hospital NHS Foundation Trust under their remit as Sponsor, the Trial Coordination Centre and other regulatory bodies to ensure compliance with Good Clinical Practice. All necessary data and documents will be made available for inspection.

8. Ethical Considerations

All trial activity will adhere to ICH Good Clinical Practice (GCP) and all applicable local trust policies. Before the start of the trial, or implementation of any amendment, approval of the trial protocol, protocol amendments, informed consent forms and other relevant documents e.g., advertisements and GP information letters if applicable, will be obtained from the Regional Ethics Committee (REC) and Health Research Authority (HRA). The Trial Manager will work with the Sponsor to assist the local sites with assessing, arranging and confirming their capacity and capability to deliver the trial, in line with the HRA approval process. All correspondence with the REC and HTA will be retained in the Trial Master File (Sponsor File/Investigator Site File).

Annual reports will be submitted to the REC in accordance with national requirements. It is the Chief Investigators responsibility to produce the annual reports as required.

There are no anticipated ethical issues with the trial design. Feedback from the patient and public involvement group has guided the trial design on the most appropriate time of approach and consent. As part of the trial design, patients will be asked to provide consent for their personal details such as name, date of birth, address, email address, NHS number, GP name and address to be transferred to Royal Papworth Hospital NHS Foundation Trust so that the central clinical trials unit staff can complete blinded follow-ups. This will be fully explained in the patient information sheet and consent form.

9. Data Protection and Patient Confidentiality

All Investigators and trial site staff must comply with the requirements of the Data Protection Act 2018 with regards to the collection, storage, processing and disclosure of personal information and will uphold the Act’s core principles.

All data used in the formulation of reports to Investigators, the Sponsor, Funder or Ethics will only contain anonymised data. The Data Management lead will ensure confidentiality of data is preserved when the data is transmitted to the Sponsor and Co-Investigators.

Patient identifiable information will be stored until the end of the trial at 3 years: the data will remain stored for 15 years as per the Trust policy. The trial data will be exported from OpenClinica and archived locally on Royal Papworth Hospital NHS Foundation Trust servers. Professor Andrew Klein will act as custodian for the data.

10. Publication Policy

All publications and scientific presentations relating to the trial will be authorised by the trial management group and submitted to the NIHR for approval at least 28 days prior to publication. Authorship will be determined according to the international committee of medical journal editors (www.icmje.org) recommendations for the conduct, reporting, editing and publication of scholarly work in medical journals. Authorship of parallel studies or sub-studies initiated outside of the trial management group will be according to the individuals involved in the project but must acknowledge the contribution of the NOTACS management group and Royal Papworth Hospital NHS Foundation Trust.

11. References

1. Filsoufi F, Rahmanian PB, G CJ, Chikwe J, Adams DH. Predictors and early and late outcomes of respiratory failure in contemporary cardiac surgery. CHEST. 2008;133:713-21.
2. Westerlind A, Nilsson F, Ricksten S. The use of continuous positive airway pressure by face mask and thoracic epidural analgesia after lung transplantation. Gothenburg Lung Transplant Group. J Cardiothorac Vasc Anesth. 1999;13(3):249-52.
3. Frizzola M, Miller T, Rodriguez M. High-flow nasal cannula: impact on oxygenation and ventilation in an acute lung injury model. Pedoatr Pulmonol. 2011;46(1):67-74.
4. Parke R, Eccleston M, McGuinness S. The effects of flowon airway pressure during nasal high-flow oxygen therapy. Respir Care. 2011;56(8):1151-5.
5. Parke R, McGuinness S, Eccleston M. Nasal high-flowtherapy delivers low level positive airway pressure. Br J Anaesth. 2009;103(6):886-90.
6. Jabbari A, Alijanpour E, Tabasi S. Clinical Usage of High-flow Oxygenation in Postcardiac Surgery Patients. Ann Card Anaesth. 2019;22(1):107-8.
7. Cuquemelle E, Lellouche F. Assessment of humidification performance: still no easy method! Respir Care. 2013;58(9):1559-61.
8. Zhonghua L, Chang W, Meng S, Xue M, Xie J, Xu J, et al. The effect of high-flow nasal oxygen therapy on postoperative pulmonary complications and hospital length of stay in postoperative patients: A systematic review and meta-analysis. Journal of Intensive care Medicine. 2018.
9. Wu X. Effect of high-flow nasal cannula oxygen therapy vs conventional oxygen on adult postcardiothoracic operation: A meta-analysis. Medicine (Baltimore). 2018;97(14).
10. Zochios V, Collier T, Blaunszun G, Butchart A, Earwaker M, Jones N, et al. The effect of high-flow nasal oxygen on hospital length of stay in cardiac surgical patients: a randomised controlled trial. . Anaesthesia. 2018;73:1478-88.
11. Luengo-Fernandez R, Leal J, Gray A, Petersen S, Rayner M. Cost of Cardiovascular Diseases in the United Kingdom. Heart. 2006;92(10):1384-9.
12. Turner E, Jenks M. Cost-effectiveness analysis of the use of high-flow oxygen through nasal cannula in intensive care
13. units in NHS England Expert Review of Pharmacoeconomics & Outcomes. 2018;18(3):331-7.
14. Fleeman N. The clinical effectiveness and cost-effectiveness of heated humidified high-flow cannula compared with usual care for pre-term infants: systematic review and economic evaluation. Health Technol Assess. 2016.
15. Programme NCA. National Aduit Cardiac Surgery Audit 2014-2017 Summary report. Available at: https://www.nicor.org.uk/wp-content/uploads/2018/11/Adult-Cardiac-Surgery-Summary-Report-2014-17.pdf2018 [Assessed on 2nd May 2019]
16. Noss C, Prusinkiewicz C, Nelson G, Patel P, Augoustides J, Gregory A. Enhanced recovery for Cardiac surgery. J Cardiothorac Vasc Anesth. 2018;32(6):2760-70.
17. Fleming I, Garratt C, Guha R, Desai J, Chaubey S, Wang Y, et al. Aggregation of marginal gains in cardiac surgery: Feasibility of a perioperative care bundle for enhanced recovery in cardiac surgical patients. J Cardiothorac Vasc Anesth. 2016;30(3):665-70.
18. Richens D. Cardiothoracic Surgery GIRFT Programme National Specialty Report. Available at: https://gettingitrightfirsttime.co.uk/wp-content/uploads/2018/04/GIRFT-Cardiothoracic-Report-1.pdf2018 [Assessed on 16th April 2019]
19. Myles PS, Shulman MA, Heritier S, Wallace S, McLlroy DR, McCluskey S, et al. Validation of Days at Home as an Outcome measure after cardiac surgery. A Prospective cohort study in Australia. BMJ Open. 2017;7(8).
20. Programme NCA. National Aduit Cardiac Surgery Audit 2014-2017 Summary report. Available at: https://www.nicor.org.uk/wp-content/uploads/2018/11/Adult-Cardiac-Surgery-Summary-Report-2014-17.pdf2018 [Assessed on 2nd May 2019]
21. Hyder J, Hirschberg R, Nguyen L. Home Discharge as a Performance Metric for Surgery. JAMA Surgery. 2015;150(2):96-7.

22. Abbott T, Fowler A, Pelosi P. A systematic review and consensus definitions for standardised end-points in peri-operative medicine: pulmonary complications. Br J Anaeth. 2018;120:1066-79.
23. Rogers CA, Pike K, Angelini GD, Reeves BC, Glauber M, Ferrarini M, et al. An open randomized controlled trial of median sternotomy versus anterolateral left thoracotomy on morbidity and health care resource use in patients having off-pump coronary artery bypass surgery: The Sternotomy Versus Thoracotomy (STET) trial. The Journal of Thoracic and Cardiovascular Surgery. 2013;146(2):306-16.
24. Devlin N, Shah K, Feng Y, Mulhern B, Van Hout B. Valuing health-related quality of life: An EQ-5D-5L value set for England. Health Economics. 2018;27(1):7-22.
25. Kingsley C, Patel S. Patient-reported outcome measures and patient-reported experience measures. BJA. 2017;17(4):137-44.
26. Schulman M, Myles P, Chan M, McIlroy D, Wallace S, Ponsford J. Measurement of Disability-free survival after surgery. Anesthesiology. 2015;122(3):524-36.
27. Society BT. British Thoracic Society and Scottish Intercollegiate Guidelines Network. British Guideline on the management of asthma. A national clinical guideline, London. Available at: https://www.brit-thoracic.org.uk/document-library/clinical-information/asthma/btssign-asthma-guideline-2014/2014 [Assessed on 17th April 2019]
28. Excellence NIfHac. Chronic obstructive pulmonary disease in over 16's; diagnosis and management NICE guideline. Available at: https://www.nice.org.uk/guidance/cg101/chapter/1-Guidance#diagnosing-copd2010 [Assessed on 31st March 2019]
29. Payakachat N, Ali MM, Tilford M. Can the EQ-5D Detect Meaningful Change? A Systematic Review. PharmacoEconomics. 2015;33(11):1137-54.
30. Dolan P. Modeling Valuations for EuroQol Health States. Medical Care. 1997;35(11):1095-108.
31. Zochios V, Collier T, Blaunszun G, Butchart A, Earwaker M, Jones N, et al. The effect of high-flow nasal oxygen on hospital length of stay in cardiac surgical patients: a randomised controlled trial. . Anaesthesia. 2018;73:1478-88.
32. Anisimov, V. (2009) Predictive modelling of recruitment and drug supply in multicenter clinical trials. Proc. of Joint Statistical Meeting. page: 1248-1259
33. Dimairo, M., Pallmann, P., Wason, J. et al. The adaptive designs CONSORT extension (ACE) statement: a checklist with explanation and elaboration guideline for reporting randomised trials that use an adaptive design. Trials 21, 528 (2020). https://doi.org/10.1186/s13063-020-04334-x
34. Unit PaSSR. Unit Costs of Health and Social Care Professionals. Available at: https://www.pssru.ac.uk/2018 [Assessed on 3rd May 2019]
35. NICE. NICE Position statement on the EQ-5D-5L. Available at: https://euroqol.org/NICE-POSITION-STATEMENT-ON-THE-EQ-5D-5L/2017 [Assessed on 11th April 2019]
36. Williams R, Rankin N, Smith T, Galler D, Seakins P. Relationship between the humidity and temperature of inspired gas and the function of the airway mucosa . . Critical Care. 1996;24.
37. Kearns B, Ara R, Wailoo A. Good practice guidelines for the use of statistical regression models in economic evaluation. Pharmaoeconomics. 2013;31:643-52.
38. Excellence NIoHaC. NICE Guide to the methods of technology appraisal 2013. Available at: http://www.nice.rg.uk/process/pmg9/resources/guide-to-the-methods-of-technology-appraisal-2013-pdf-20079758437812013 [Assessed on 20th March 2019]
39. 26 Sacco RL, Kasner SE, Broderick JP, et al. An updated definition of stroke for the 21st Century. Stroke 2013; 44:2 064-89.
Singer M, Deutschmann CS, Seymour CW, et al. The third international consensus definitions for sepsis and septic shock. JAMA. 2016 Feb 23; 315(8): 801–810.
40. Kidney Disease Improving Global Outcomes (KDIGO) Clinical Practice Guideline for Acute Kidney Injury. Kidney Int Suppl. 2012;2:1–138.
41. Thygesen K, Alpert JS, Jaffe AS, et al. Fourth universal definition of myocardial infarction (2018) European Heart Journal, Volume 40, Issue 3, 14 January 2019, Pages 237–269, https://doi.org/10.1093/eurheartj/ehy462

12. Appendices

12.1 Appendix 1: Exploratory Secondary Outcome Definitions

Definition of stroke: The term "stroke" should be broadly used to include all of the following:

- *Definition of CNS infarction:* CNS infarction is brain, spinal cord, or retinal cell death attributable to ischemia, based on1. pathological, imaging, or other objective evidence of cerebral, spinal cord, or retinal focal ischemic injury in a defined vascular distribution; or2. clinical evidence of cerebral, spinal cord, or retinal focal ischemic injury based on symptoms persisting ≥24 hours or until death, and other etiologies excluded. (Note: CNS infarction includes hemorrhagic infarctions, types I and II; see "Hemorrhagic Infarction.")
- *Definition of ischemic stroke:* An episode of neurological dysfunction caused by focal cerebral, spinal, or retinal infarction. (Note: Evidence of CNS infarction is defined above.)
- *Definition of silent CNS infarction:* Imaging or neuropathological evidence of CNS infarction, without a history of acute neurological dysfunction attributable to the lesion.
- *Definition of intracerebral hemorrhage*: A focal collection of blood within the brain parenchyma or ventricular system that is not caused by trauma.(Note: Intracerebral hemorrhage includes parenchymal hemorrhages after CNS infarction, types I and II—see "Hemorrhagic Infarction.")
- *Definition of stroke caused by intracerebral hemorrhage:* Rapidly developing clinical signs of neurological dysfunction attributable to a focal collection of blood within the brain parenchyma or ventricular system that is not caused by trauma.
- *Definition of silent cerebral hemorrhage:* A focal collection of chronic blood products within the brain parenchyma, subarachnoid space, or ventricular system on neuroimaging or neuropathological examination that is not caused by trauma and without a history of acute neurological dysfunction attributable to the lesion.
- *Definition of subarachnoid hemorrhage:* Bleeding into the subarachnoid space (the space between the arachnoid membrane and the pia mater of the brain or spinal cord).
- *Definition of stroke caused by subarachnoid hemorrhage*: Rapidly developing signs of neurological dysfunction and/or headache because of bleeding into the subarachnoid space (the space between the arachnoid membrane and the pia mater of the brain or spinal cord), which is not caused by trauma.
- *Definition of stroke caused by cerebral venous thrombosis*: Infarction or hemorrhage in the brain, spinal cord, or retina because of thrombosis of a cerebral venous structure. Symptoms or signs caused by reversible edema without infarction or hemorrhage do not qualify as stroke.
- *Definition of stroke, not otherwise specified:* An episode of acute neurological dysfunction presumed to be caused by ischemia or hemorrhage, persisting ≥24 hours or until death, but without sufficient evidence to be classified as one of the above (38).

Definition of sepsis:
Sepsis is defined as life-threatening organ dysfunction caused by a dysregulated host response to infection. Organ dysfunction can be identified as an acute change in total SOFA score ≥2 points consequent to the infection. In lay terms, sepsis is a life-threatening condition that arises when the body's response to an infection injures its own tissues and organs. Septic shock is a subset of sepsis in which underlying circulatory and cellular/metabolic abnormalities are profound enough to substantially increase mortality. Patients with septic shock can be identified with a clinical construct of sepsis with persisting hypotension requiring vasopressors to maintain MAP ≥65 mm Hg and having a serum lactate level >2 mmol/L despite adequate volume resuscitation (39).

Definition of acute kidney injury (AKI)

AKI definition and staging according to KDIGO criteria

AKI is *defined* as any of the following:

1	Increase in sCr ≥26.5 µmol/L within 48 hours; or
2	Increase in sCr ≥1.5 times baseline, which is known or presumed to have occurred within the prior 7 days; or
3	Urine volume <0.5 mL/kg/h for 6 hours.

AKI is *staged for severity* according to the following criteria

Stage 1	1.5–1.9 times baseline OR ≥26.5 µmol/L) absolute increase in sCr	Urine volume <0.5 mL/kg/h for 6–12 hours
Stage 2	sCr ≥2.0–2.9 times baseline sCr ≥3.0 times from baseline OR	Urine volume <0.5 mL/kg/h for ≥12 hours
Stage 3	Increase in sCr to ≥353.6 µmol/L OR Initiation of renal replacement therapy OR, In patients <18 years, decrease in eGFR to <35 mL/min per 1.73 m^2	Urine volume <0.3 mL/kg/h for ≥24 hours OR Anuria for ≥12 hours

sCr=serum creatinine, eGFR= estimated glomerular filtration rate (40).

Definition of myocardial infarction

Detection of a rise of cardiac Troponin values with at least one value above the 99th percentile URL and with at least one of the following:

- Symptoms of acute myocardial ischaemia;
- New ischaemic ECG changes;
- Development of pathological Q waves;
- Imaging evidence of new loss of viable myocardium or new regional wall motion abnormality in a pattern consistent with an ischaemic aetiology;
- Identification of a coronary thrombus by angiography including intracoronary imaging or by autopsy (41).

12.2 Appendix 2: Trial Extubation Protocol

(Mechanical ventilation and tracheal extubation after cardiac surgery)

Patients' lungs will typically be mechanically ventilated with FiO2 40-60%, PEEP 5-10 cm H2O, tidal volume (TV) 5-8 ml/kg ideal body weight and RR 10-20 breaths/min to achieve PaO2 > 8 kPa, PaCO2 4-6 KPa and peak pressure < 30 cms H2O. If failing to achieve these parameters, ventilator settings may be adjusted, and medical team consulted for advice.

The aim is to wean the patient from mandatory ventilation and switch to spontaneous breathing using pressure support (PS) / continuous positive airway pressure (CPAP) as soon as possible. Once the patient is awake and breathing spontaneously, test the patient's ability to breathe while receiving minimal ventilator support via a spontaneous breathing trial (SBT) using PS/CPAP, FiO2 <40%, PS 5-10cm H20 and PEEP 5- 10cm H20.

If after spontaneous breathing trial, the patient remains stable, there are no signs of respiratory distress and oxygen saturations > 93% with inspired oxygen less than or equal to 60%, the patient's trachea should be extubated.

If not ready for extubation then re-assess and repeat SBT as appropriate. If patient continually fails SBT then discuss with medical team.

To proceed to extubation patients should be:
− able to follow commands
− able to protect own airway
− have adequate strength (e.g. lift head off pillow)
− have adequate respiratory effort
− haemodynamically stable
− bleeding within expected limits (as per local protocol)
- adequately reversed (neuromuscular blockade)

After extubation, immediately apply high-flow nasal therapy or standard oxygen depending on group allocation.

High-flow nasal therapy

High-flow nasal therapy equipment and disposables should be prepared in advance and checked while patient's lungs still being mechanically ventilated.
Start at 40% inspired O2 and flow 30 l/min then up to 50 l/min over 5-10 min. Monitor saturations and RR and arterial gases after 15 min then as per local policy. If saturations < 93% then increase FiO2 as per respiratory escalation protocol.

Standard oxygen therapy

Start 30-40% inspired O2 and flow 2-6 l/min via nasal prongs or non-rebreathing mask (not humidified and not heated). Monitor saturations and RR and arterial gases after 15 min then as per local policy. If saturations < 93% then increase FiO2 as per respiratory escalation protocol.

*Ideal body weight is the weight corresponding to an ideal body mass index of 22 kg/m2

Men IBW = (height in metres)2 x 22

Women, IBW = IBW = (height in metres - 10cm)2 x 22

12.3 Appendix 3: Trial Escalation of Respiratory Therapy Protocol

All patients on oxygen therapy (HFNT or standard therapy) should have regular pulse oximetry measurements. The frequency of oximetry measurements will depend on the stability of the patient. Critically ill patients should have their oxygen saturations monitored continuously and recorded every few minutes whereas patients with mild breathlessness will need less frequent monitoring. Oxygen therapy should be increased if the saturation is < 93% and decreased if the saturation is > 95% (and eventually discontinued as the patient recovers).

Any sudden fall in oxygen saturation should lead to clinical evaluation of the patient and in most cases, measurement of blood gases. All peri-arrest and critically ill patients should be given 100% oxygen (15 l/min reservoir mask) whilst awaiting immediate medical review.

Escalation of respiratory therapy may be indicated if:
-Saturations < 93%
-RR > 20 breaths/min
-PaCO2 > 7 kPa

PLAN A

Assess patient, consider chest x-ray

Increase FiO2 in increments of 10% up to a maximum of 60%.

If patient is receiving high-flow nasal therapy, consider increasing flow up to max 60 l/min

PLAN B

Assess patient, consider chest X-ray and arterial blood gas

Consider transfer to Level 2 or Level 3 care environment (HDU or ICU)

Increase FiO2 up to a maximum of 60%
Consider CPAP (mask or nasal mask or hood), start at 5 cm H2O

Consider non-invasive ventilation (NIV) or BiPAP

PLAN C

Assess patient, consider chest X-ray and arterial blood gas

Consider invasive mechanical ventilation (requires tracheal intubation)

Clinicians can move between Plans A, B and C depending on the patient’s condition and not necessarily in that order.

12.4 Appendix 4: International Management and Governance Structure

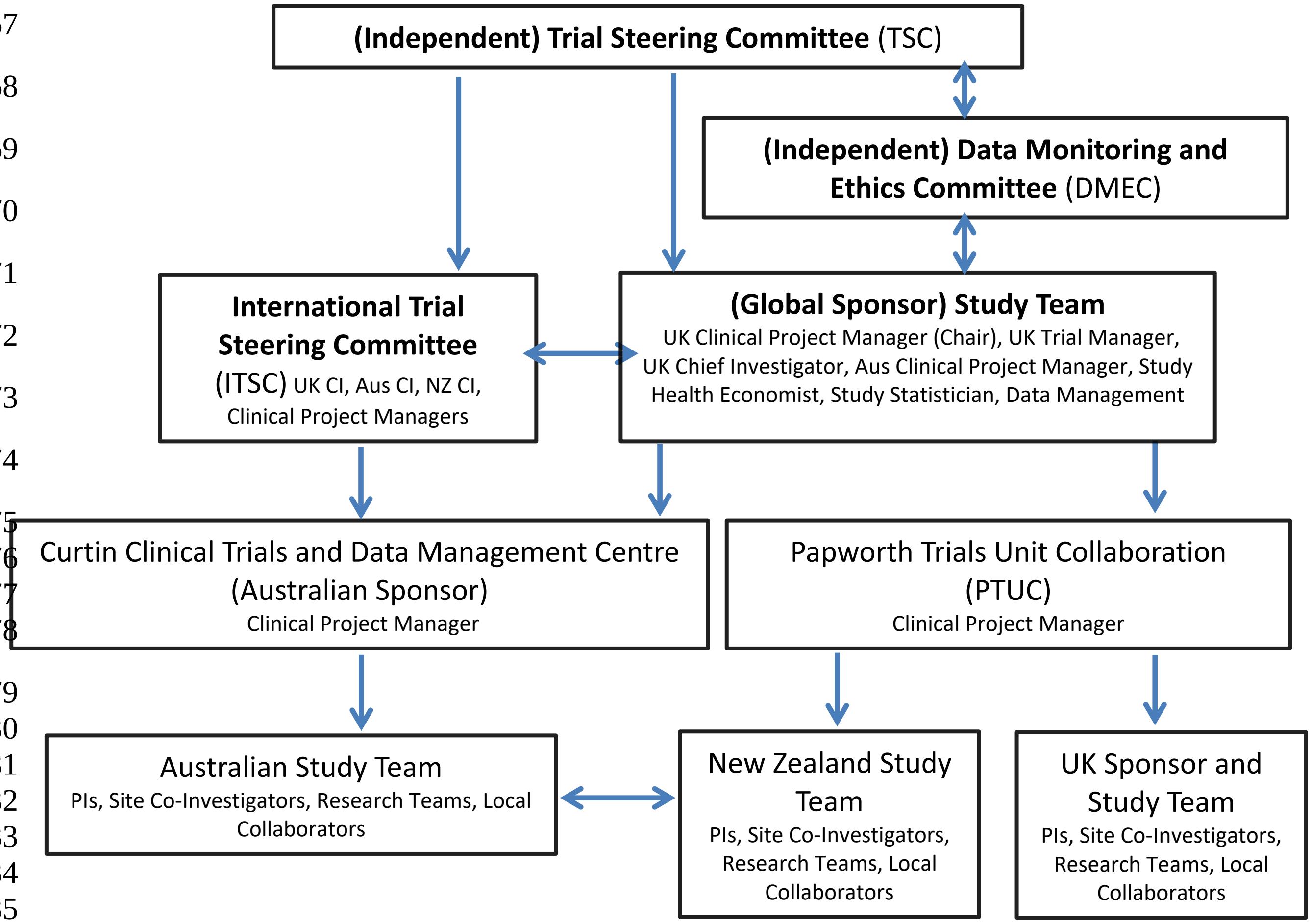

12.5 Appendix 5. Telephone Interview Escalation Protocol NOTACS study

Purpose: To clearly outline steps required if a study participant identifies as severely/extremely anxious or depressed during a follow-up interview.

Responsibility: Member of the central trials team undertaking follow-up interview in consultation with Principal Investigator

Introduction: It is important to recognise that a study participant may become distressed during a follow-up interview, or report being severely/extremely anxious or depressed. This protocol aims to manage this situation.

If the participant reports feeling severely/extremely anxious or depressed all follow-up questionnaires will be ceased and the member of the central trials team will ascertain whether the participant is alone. The interviewer will then ask if the participant would like the member of the central trials team to speak with a relative or friend; or general practitioner (GP) on the participants behalf. The member of the central trials team will also provide the participant with contact details for support services and resources, or offer to contact these services on the participant's behalf to arrange a follow-up phone call.

If the participant ends the phone call without this information exchange, the member of the central trials team will attempt further contact with the participant to ensure their welfare. If unable to make contact with them the member of the central trials team may choose to escalate this to emergency services (if there was judged to be a threat to life) or to the participants General Practitioner for follow-up as soon as possible.

If the member of the central trials team experiences any distress as a result of a participant's responses, requires advice or support the site Principal Investigator is to be contacted.

All issues raised by the patient should be documented, all interventions that have been offered or actions taken in a note to file. Trained assessors can only advise; patients may not necessarily be receptive. Please ensure that this is reported to the Chief Investigator.

Resources

Police or Ambulance	Dial 999
Mind Charity	https://www.mind.org.uk/information-support/guides-to-support-and-services/crisis-services/helplines-listening-services/
National Suicide Prevention Helpline UK	0800 689 5652
Age UK	0800 678 1602
Giveusashout Text line	Text 85258
Samaritans	166 123
Cris Text line	Text 85258
NHS Urgent Mental Health Helpline Finder (UK)	https://www.nhs.uk/service-search/mental-

	health/find-an-urgent-mental-health-helpline

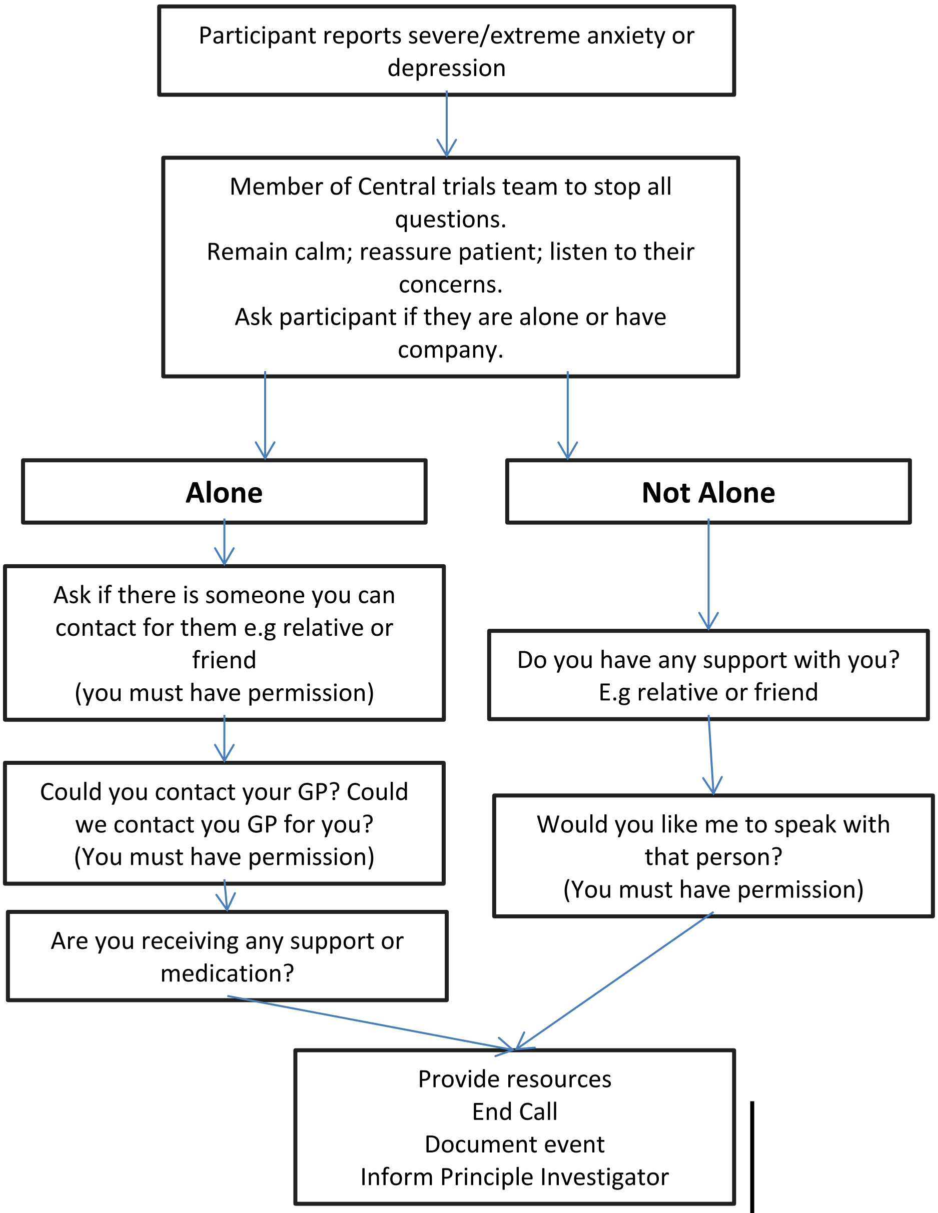

12.6 Appendix 6. Details of Interim Analysis

The interim sample size re-estimation was performed after 300 patients had completed 90 days post-randomisation follow-up. During the analyses process it was confirmed that 241 of the 300 patients included had complete 90-day follow-up data at the time of the interim analysis. The interim sample size re-estimation was performed by an independent statistician to allow the trial statisticians to remain blinded, in order to preserve the type 1 error rate at 5%. This sample size adaptation was pre-planned as part of the adaptive design, to prevent an underpowered trial if moderate deviations from the assumptions made for the initial sample size calculation were observed. Based on the results of the interim sample size re-estimation, the recommendation of the Data Monitoring and Ethics Committee (DMEC) was to increase the maximum sample size to 1280. Therefore, the final sample size has been increased from the original minimum of 850 to 1280 patients. The UK Funder (NIHR HTA) was in agreement with this maximum sample size increase and has granted an 18-month extension (Variation to Contract) to the grant. The maximum sample size of 1280 is feasible at the end of the extended recruitment period of the trial, at Month 57.
