# Supplementary material for: High-Flow Nasal Oxygen Therapy After Cardiac Surgery: A Randomized Clinical Trial
Source: JAMA Netw Open. 2026 Apr 8;9(4):e265447. doi: 10.1001/jamanetworkopen.2026.5447 (PMC13063085; doi:10.1001/jamanetworkopen.2026.5447)
Supplement: Supplement 2. — eMethods 1. Trial Escalation of Respiratory Therapy Protocol eMethods 2. Calculation of Days at Home in the First 90 Days after Surgery (DAH90) eFigure 1. Display of Patient Locations Over 90 Days for a Subset of Patients in the NOTACS Trial eTable 1. Missing Data for Primary and Secondary Analysis Variables eTable 2. Number of Patients at Each Study Site by Treatment Arm and Study Stage eTable 3. Baseline Ethnicity by Treatment Arm eTable 4. Baseline Residence by Treatment Arm eTable 5. Baseline Participant and Family Resource Use Questionnaire by Treatment Arm eFigure 2. Adherence With Randomized Therapy, Time on Randomized and Nonrandomized Treatment Arms for Patients Randomized to Nasal High-Flow Nasal Oxygen Therapy (NHFOT) or Standard Oxygen Therapy (SOT) eTable 6. Median Quantile Regression Between Response Variable DAH90 and Treatment Group, Adjusted for Other Selected Baseline Variable eTable 7. Comparison of Postextubation ROX Indexes by Treatment Group eTable 8. Baseline EQ-5D-5L Questionnaire by Treatment Arm eTable 9. Discharge EQ-5D-5L Questionnaire by Treatment Arm eTable 10. Day 30 EQ-5D-5L Questionnaire by Treatment Arm eTable 11. Day 90 EQ-5D-5L Questionnaire by Treatment Arm eTable 12. Baseline Barthel Index Questionnaire by Treatment Arm eTable 13. Discharge Barthel Index Questionnaire by Treatment Arm eTable 14. Day 30 Barthel Index Questionnaire by Treatment Arm eTable 15. Day 90 Barthel Index Questionnaire by Treatment Arm eTable 16. Barthel Index Questionnaire at Baseline, Discharge, Day 30 and Day 90, Ignoring Treatment Arm eTable 17. Discharge Participant and Family Resource Use Questionnaire by Treatment Arm eTable 18. Discharge Destination by Treatment Arm eTable 19. Frequency and Duration (Days) of Nonhospital Stays Away From Home Following Discharge From Index Hospital Admission by Treatment Arm eTable 20. Incidence Rate of Serious Adverse Events of Special Interest by Treatment Arm eTable 21. Summary of Adverse Events (Excludes Serious [file jamanetwopen-e265447-s002.pdf]

Supplementary Online Content

Litton E, Parke L, McGuinness SP, et al; the Nasal High-Flow Oxygen Therapy After Cardiac Surgery Investigators. High-flow nasal oxygen therapy after cardiac surgery: a randomized clinical trial. *JAMA Netw Open*. 2026;9(4):e265447. doi:10.1001/jamanetworkopen.2026.5447

eMethods 1. Trial Escalation of Respiratory Therapy Protocol

eMethods 2. Calculation of Days at Home in the First 90 Days after Surgery (DAH90)

eFigure 1. Display of Patient Locations Over 90 Days for a Subset of Patients in the NOTACS Trial

eTable 1. Missing Data for Primary and Secondary Analysis Variables

eTable 2. Number of Patients at Each Study Site by Treatment Arm and Study Stage

eTable 3. Baseline Ethnicity by Treatment Arm

eTable 4. Baseline Residence by Treatment Arm

eTable 5. Baseline Participant and Family Resource Use Questionnaire by Treatment Arm

eFigure 2. Adherence With Randomized Therapy, Time on Randomized and Nonrandomized Treatment Arms for Patients Randomized to Nasal High-Flow Nasal Oxygen Therapy (NHFOT) or Standard Oxygen Therapy (SOT)

eTable 6. Median Quantile Regression Between Response Variable DAH90 and Treatment Group, Adjusted for Other Selected Baseline Variables

eTable 7. Comparison of Postextubation ROX Indexes by Treatment Group

eTable 8. Baseline EQ-5D-5L Questionnaire by Treatment Arm

eTable 9. Discharge EQ-5D-5L Questionnaire by Treatment Arm

eTable 10. Day 30 EQ-5D-5L Questionnaire by Treatment Arm

eTable 11. Day 90 EQ-5D-5L Questionnaire by Treatment Arm

eTable 12. Baseline Barthel Index Questionnaire by Treatment Arm

eTable 13. Discharge Barthel Index Questionnaire by Treatment Arm

eTable 14. Day 30 Barthel Index Questionnaire by Treatment Arm

eTable 15. Day 90 Barthel Index Questionnaire by Treatment Arm

eTable 16. Barthel Index Questionnaire at Baseline, Discharge, Day 30 and Day 90, Ignoring Treatment Arm

eTable 17. Discharge Participant and Family Resource Use Questionnaire by Treatment Arm

© 2026 Litton E et al. *JAMA Network Open*.

eTable 18. Discharge Destination by Treatment Arm

eTable 19. Frequency and Duration (Days) of Nonhospital Stays Away From Home Following Discharge From Index Hospital Admission by Treatment Arm

eTable 20. Incidence Rate of Serious Adverse Events of Special Interest by Treatment Arm

eTable 21. Summary of Adverse Events (Excludes Serious Adverse Events) By Treatment Arm

eTable 22. Summary of Serious Adverse Events by Treatment Arm

This supplementary material has been provided by the authors to give readers additional information about their work.

© 2026 Litton E et al. *JAMA Network Open*.

eMethods 1. Trial Escalation of Respiratory Therapy Protocol

All patients on oxygen therapy (nasal high-flow oxygen therapy or standard therapy) should have regular pulse oximetry measurements. The frequency of oximetry measurements will depend on the stability of the patient. Critically ill patients should have their oxygen saturations monitored continuously and recorded every few minutes whereas patients with mild breathlessness will need less frequent monitoring. Oxygen therapy should be increased if the saturation is < 93% and decreased if the saturation is > 95% (and eventually discontinued as the patient recovers).

Any sudden fall in oxygen saturation should lead to clinical evaluation of the patient and in most cases, measurement of blood gases. All peri-arrest and critically ill patients should be given 100% oxygen (15 l/min reservoir mask) whilst awaiting immediate medical review.

Escalation of respiratory therapy may be indicated if:
- Oxygen saturations < 93%
- Respiratory rate (RR) > 20 breaths/min
- PaCO2 > 7 kPa

Plan A
Assess patient, consider chest x-ray
Increase FiO2 in increments of 10% up to a maximum of 60%.
If patient is receiving high-flow nasal therapy, consider increasing flow up to max 60 l/min

Plan B
Assess patient, consider chest X-ray and arterial blood gas
Consider transfer to level 2 or level 3 care environment (HDU or ICU)
Increase FiO2 in increments of 10% up to a maximum of 100%
Consider CPAP (mask or nasal mask or hood), start at 5 cm H2O
Consider non-invasive ventilation (NIV) or BiPAP

Plan C
Assess patient, consider chest X-ray and arterial blood gas
Consider invasive mechanical ventilation (requires tracheal intubation)

© 2026 Litton E et al. *JAMA Network Open*.

Clinicians can move between plans A, B and C depending on the patient's condition and not necessarily in that order.

© 2026 Litton E et al. *JAMA Network Open*.

eMethods 2. Calculation of Days at Home in the First 90 Days after Surgery (DAH90)

The NOTACS study's primary outcome was DAH90, a patient-centered endpoint defined as days alive and at home post-surgery spent in their baseline place of residence. This measure as well as the 90-day follow-up period, were chosen based on input from patients and carers, who prioritized this outcome over the length of the initial hospital stay post-surgery (NIHR HTA grant application, Reference Number: NIHR128351).

Data collection was conducted in three distinct phases of the patient's journey:

- **Baseline Data Collection:** A form was used to record the patient's living situation and support needs *before* hospital admission.
- **In-Hospital Data Collection:** A separate form was used to capture the duration of the patient's first hospital stay. It is noted that a question about support was omitted as it is assumed support is always provided in the hospital.
- **Post-Discharge Data Collection:** A participant diary was used to continuously track changes to the patient's location and their support requirements, and the start and end dates of these different location/support combinations after they left the hospital. Although the patient diary captures periods of time, we transformed this data to represent individual days; for example, a one-week period in the diary would be represented as seven separate rows in the dataset, with one row per day.

Therefore, to calculate DAH90 for each patient, a total of 182 data points were required (with a few exceptions, e.g., if the patient died within the 90 day follow-up then DAH90 is assumed to be 0, or if the support variable is missing but the patient is in hospital, support is assumed):

- Location at baseline (1 data point)
- Support at baseline (1 data point)
- Location for each day of the 90-day follow-up (90 data points)
- Support for each day of the 90-day follow-up (90 data points)

Locations were categorized into: Home, Hospital, Nursing home, Relative’s/friend’s home, Residential home, Respite care, and Other.

© 2026 Litton E et al. *JAMA Network Open*.

eFigure 1. Display of Patient Locations Over 90 Days for a Subset of Patients in the NOTACS Trial

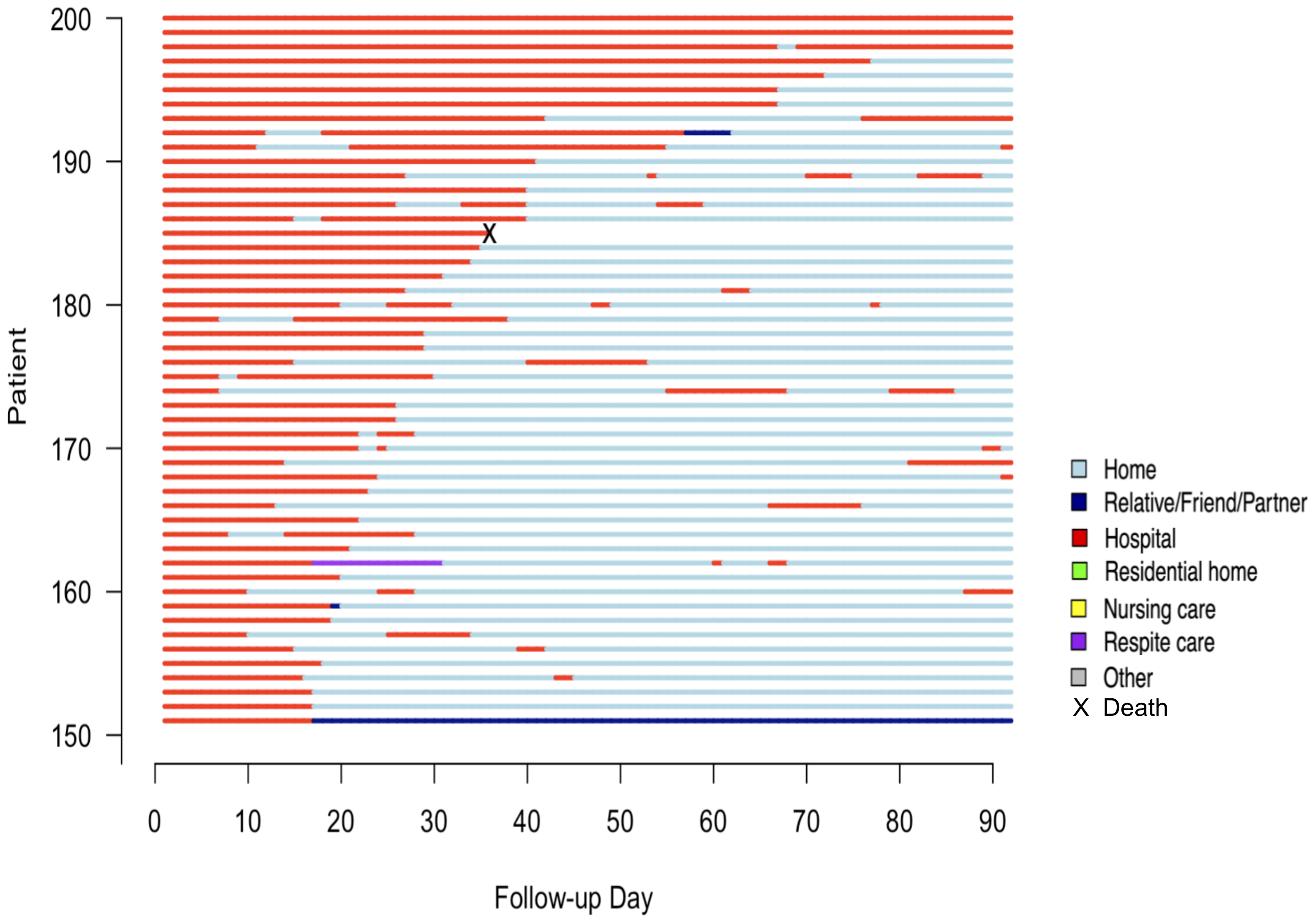

© 2026 Litton E et al. *JAMA Network Open*.

The intent in including a support element to the DAH endpoint described by Myles et al. (2017) was to add more granularity to whether patients were experiencing better or worse health during the 90 days of follow-up compared to their baseline. For instance, a patient starting at home without support who then stays with family due to a need for support is in a different situation than a patient staying with family purely for pleasure, such as a holiday. Pre-specification prior to database lock provided a clear and transparent rationale for how DAH values were to be calculated from the data collected from patients once the full dataset was received by the trial statistician.

		Escalation of care/time away from "usual abode" during follow-up period										
		Home without support	Home with support	Relative or friend' s home without support	Relative or friend' s home with support	Residential home without support	Residential home with support	Respite care with or without support△	Nursing home with or without support△	Hospital with or without support△	Other without support	Other with support
Baseline Usual abode	Home without support	N	Y	N	Y	Y	Y	Y	Y	Y	*	*
	Home with support	N	N	N	Y	Y	Y	Y	Y	Y	*	*
	Relative or friend's home without support	N	Y	N	Y	Y	Y	Y	Y	Y	*	*
	Relative or friend's home with support	N	N	N	N	Y	Y	Y	Y	Y	*	*
	Residential home without support	N	N	N	N	N	Y	Y	Y	Y	*	*
	Residential home with support	N	N	N	N	N	N	Y	Y	Y	*	*
	Respite care with or without support△	N	N	N	N	N	N	N	Y	Y	*	*
	Nursing home with or without support△	N	N	N	N	N	N	N	N	Y	*	*
	Hospital with or without support△	N	N	N	N	N	N	N	N	N	*	*
	Other without support	N	*	*	*	*	*	*	*	*	*	*
	Other with support	N	*	*	*	*	*	*	*	*	*	*

** To be decided case-by-case.*

△ There are some options that could be chosen in the eCRF which are not possible in reality (i.e., locations where support is provided by default). These have been merged (e.g., hospital with or without support)

This table, reproduced here for convenience and completeness, is taken directly from the published SAP (Dawson, S.N., Chiu, YD., Klein, A.A., Earwaker M, Villar S. S. Effect of high-flow nasal therapy on patient-centred outcomes in patients at high risk of postoperative pulmonary

© 2026 Litton E et al. *JAMA Network Open*.

complications after cardiac surgery: update to the statistical analysis plan for NOTACS, a multicentre adaptive randomised controlled trial. Trials 25, 741 (2024). https://doi.org/10.1186/s13063-024-08538-3).

For the calculation of DAH90, each patient had 90 rows of data that were either a Y or N based on the table above, reflecting whether each of those 90 days was considered as a day away from home (Y) or not (N) compared to baseline. For each patient, the calculation of DAH90 was 90 minus the number of Y responses coming from the table above (with DAH90=0 for any patient who died during the follow-up period).

© 2026 Litton E et al. *JAMA Network Open*.

eTable 1. Missing Data for Primary and Secondary Analysis Variables

Variable	Missing
DAH90, %	4.4
DAH30, %	3.3
Treatment group	0
ARISCAT score, No. (%)	9 (0.7)
EUROSCORE II, No. (%)	81 (6.3
COPD, No. (%)[1]	1054 (82.3)
Asthma, No. (%)[1]	936 (73.1)
Obesity (BMI>35kg/m2) , No. (%)[1]	761 (59.5)
Current smoker, No. (%)[1]	906 (70.8)
Lower respiratory tract infection in the last 4 weeks, No. (%)[1]	1243 (97.1)
Age at randomisation, No. (%)	4 (0.3)
First time or redo surgery, No. (%)	2 (0.2)
ROX index: 2 hours, No. (%)	52 (4.1)
ROX index: 6 hours, No. (%)	43 (3.4)
ROX index: 12 hours, No. (%)	47 (3.7)
ROX index: 24 hours, No. (%)	52 (4.1)
ROX index: 48 hours, No. (%)	74 (5.8)
Extubation timing, No. (%)	16 (1.2)
Return to theatre within 24 hours of admission to ICU, No. (%)	13 (1.0)
Length of initial ICU stay, No. (%)	46 (3.6)

[1]These risk factors were only recorded in the database when present, therefore it is not possible to separate missing data from risk factors that were not present.

© 2026 Litton E et al. *JAMA Network Open*.

eTable 2. Number of Patients at Each Study Site by Treatment Arm and Study Stage

Site	Date opened to recruitment	Treatment arm	Number randomised	Number completed 30-day follow-up	Number completed 90-day follow-up
Royal Papworth Hospital	30/09/2020	Standard oxygen therapy	99	97	96
		**High-flow nasal therapy**	100	98	97
Royal Brompton Hospital	14/07/2021	Standard oxygen therapy	28	28	28
		**High-flow nasal therapy**	29	26	26
Kings College London Hospital	03/11/2021	Standard oxygen therapy	40	40	40
		**High-flow nasal therapy**	41	41	41
Cardiff and Vale Hospital	19/08/2021	Standard oxygen therapy	29	29	28
		**High-flow nasal therapy**	29	26	25
Golden Jubilee Hospital	21/10/2021	Standard oxygen therapy	47	42	40
		**High-flow nasal therapy**	47	41	40
Guys and St Thomas' Hospital	09/02/2022	Standard oxygen therapy	53	52	50
		**High-flow nasal therapy**	53	53	52
Glenfield Hospital	06/05/2022	Standard oxygen therapy	33	31	30
		**High-flow nasal therapy**	31	29	28
Auckland City Hospital	29/11/2021	Standard oxygen therapy	77	76	76
		**High-flow nasal therapy**	78	77	77
James Cook Hospital	25/08/2022	Standard oxygen therapy	45	43	43
		**High-flow nasal therapy**	44	42	41
Queen Elizabeth Hospital	19/01/2024	Standard oxygen therapy	2	2	2
		**High-flow nasal therapy**	5	4	4
St John of God Hospital	11/03/2022	Standard oxygen therapy	16	15	15
		**High-flow nasal therapy**	17	17	17
Prince Charles Hospital	17/03/2022	Standard oxygen therapy	103	102	101
		**High-flow nasal therapy**	102	99	99
Fiona Stanley Hospital	29/03/2022	Standard oxygen therapy	41	39	39

© 2026 Litton E et al. *JAMA Network Open*.

Site	Date opened to recruitment	Treatment arm	Number randomised	Number completed 30-day follow-up	Number completed 90-day follow-up
		**High-flow nasal therapy**	40	39	39
The Townsville Hospital	30/08/2022	Standard oxygen therapy	12	12	12
		**High-flow nasal therapy**	11	11	11
Royal North Shore Hospital	16/01/2023	Standard oxygen therapy	7	6	6
		**High-flow nasal therapy**	5	5	5
The Alfred Hospital	06/12/2023	Standard oxygen therapy	7	7	7
		**High-flow nasal therapy**	7	7	7
University Hospital Geelong	03/05/2024	Standard oxygen therapy	1	1	1
		**High-flow nasal therapy**	1	1	1
Total		Standard oxygen therapy	640	622	614
		**High-flow nasal therapy**	640	616	610

© 2026 Litton E et al. *JAMA Network Open*.

eTable 3. Baseline Ethnicity by Treatment Arm

**Ethnicity**	**High-flow nasal therapy (n=640)**	**Standard oxygen therapy (n=640)**
UK participants, No.	378	375
Bangladeshi, No. (%)	1 (0.3)	0
Black African, No. (%)	3 (0.8)	0
Black Caribbean, No. (%)	2 (0.5)	1 (0.3)
Black Other, No. (%)	2 (0.5)	1 (0.3)
Chinese, No. (%)	1 (0.3)	1 (0.3)
Indian, No. (%)	3 (0.8)	4 (1.1)
Not Reported, No. (%)	6 (1.6)	3 (0.8)
Other, No. (%)	3 (0.8)	4 (1.1)
Other Asian, No. (%)	1 (0.3)	1 (0.3)
Other Mixed, No. (%)	1 (0.3)	0
Other White, No. (%)	4 (1.1)	7 (1.9)
Pakistani, No. (%)	3 (0.8)	1 (0.3)
White British, No. (%)	344 (91.0)	346 (92.3)
White Irish, No. (%)	4 (1.1)	6 (1.6)
Australian participants, No.	183	187
Aboriginal And/Or Torres Strait Islander, No. (%)	16 (8.7)	28 (15.0)
Central Asian, No. (%)	0	1 (0.5)
East Asian (China/Japan/Mongolia/Korea/Japan/Taiwan) , No. (%)	0	2 (1.1)
Maori/Polynesian/Pacific Islander/Fijian, No. (%)	5 (2.7)	4 (2.1)
North African/Middle-Eastern, No. (%)	1 (0.6)	0
South Asian (India/Pakistan/Bangladesh/Sri Lanka) , No. (%)	2 (1.1)	2 (1.1)
South East Asian (Vietnam/Laos/Cambodia/Myanmar/Thailand/Malaysia/Singapore/Indonesia/Brunei/Timor-Leste/The Philippines) , No. (%)	0	1 (0.5)
Sub-Saharan African, No. (%)	0	1 (0.5)
White/European/ Caucasian, No. (%)	159 (86.9)	148 (79.1)
New Zealand participants, No.	78	77

© 2026 Litton E et al. *JAMA Network Open*.

Ethnicity	High-flow nasal therapy (n=640)	Standard oxygen therapy (n=640)
Chinese, No. (%)	1 (1.3)	0
Cook Island Maori, No. (%)	5 (6.4)	2 (2.6)
Fijian, No. (%)	3 (3.9)	0
Indian, No. (%)	3 (3.9)	2 (2.6)
Niuean, No. (%)	1 (1.3)	0
Nz European, No. (%)	28 (35.9)	38 (49.4)
Nz Maori, No. (%)	18 (23.1)	25 (32.5)
Other European, No. (%)	10 (12.8)	2 (2.6)
Other Pacific Island, No. (%)	0	1 (1.3)
Samoan, No. (%)	5 (6.4)	4 (5.2)
Tongan, No. (%)	4 (5.1)	3 (3.9)

© 2026 Litton E et al. *JAMA Network Open*.

eTable 4. Baseline Residence by Treatment Arm

Variable	High-flow nasal therapy (n=640)	Standard oxygen therapy (n=640)
What is the patient's usual abode?, No. (%)		
Home	633 (98.9)	631 (98.9)
Relative's Home	0	0
Residential Home	0	4 (0.6)
Other	5 (0.8)	1 (0.2)
Requires support at their usual abode, No. (%)	30 (4.7)	40 (6.2)

© 2026 Litton E et al. *JAMA Network Open*.

eTable 5. Baseline Participant and Family Resource Use Questionnaire by Treatment Arm

**Variable**	**High-flow nasal therapy (n=640)**	**Standard oxygen therapy (n=640)**
Have stayed overnight in hospital in last month, No. (%)	368 (58.2)	365 (57.3)
If yes, how many nights did you stay in hospital?, No., median (IQR)	366, 10 (12)	364, 10 (12.2)
Have stayed overnight in a nursing home or care home in last month, No. (%)	0	0
How many times in the past month have you visited the Emergency Dept?, No. (%)		
0	246 (38.4)	263 (41.1)
1	109 (17.0%)	93 (14.5)
2	11 (1.7)	12 (1.9)
3	2 (0.3)	3 (0.5)
4	0	1 (0.2)
How many times in the past month have you seen a doctor in hospital outpatients?, No. (%)		
0	468 (73.1)	437 (68.3)
1	122 (19.1)	143 (22.3)
2	23 (3.6)	36 (5.6)
3	7 (1.1)	12 (1.9)
4	5 (0.8)	3 (0.5)
5	1 (0.2)	1 (0.2)
6	2 (0.3)	1 (0.2)
7	0	2 (0.3)
28	1 (0.2)	0
How many times in the past month have you seen a primary care doctor?, No. (%)		
0	423 (66.1)	423 (66.1)
1	157 (24.5)	157 (24.5)
2	29 (4.5)	34 (5.3)
3	13 (2.0)	11 (1.7)
4	4 (0.6)	4 (0.6)
5	2 (0.3)	3 (0.5)
6	1 (0.2)	1 (0.2)

© 2026 Litton E et al. *JAMA Network Open*.

Variable	High-flow nasal therapy (n=640)	Standard oxygen therapy (n=640)
12	0	1 (0.2)
How many times in the past month have you had a home visit from a health professional?, No. (%)		
0	611 (95.5)	620 (96.9)
1	12 (1.9)	7 (1.1)
2	3 (0.5)	2 (0.3)
3	1 (0.2)	1 (0.2)
4	0	1 (0.2)
5	0	1 (0.2)
6	1 (0.2)	0
9	0	2 (0.3)
10	0	1 (0.2)
12	1 (0.2)	0
14	0	1 (0.2)
How many times in the past month have you had visits from social support services?, No. (%)		
0	618 (96.6)	619 (96.7)
1	6 (0.9)	3 (0.5)
2	3 (0.5)	4 (0.6)
3	0	1 (0.2)
4	1 (0.2)	2 (0.3)
5	0	1 (0.2)
6	0	1 (0.2)
12	1 (0.2)	0
14	0	1 (0.2)
16	0	1 (0.2)
30	0	3 (0.5)
How many times in the past month have you spoken to a nurse or doctor by telephone?, No. (%)		
0	435 (68.0)	443 (69.2)
1	125 (19.5)	128 (20.0)

© 2026 Litton E et al. *JAMA Network Open*.

Variable	High-flow nasal therapy (n=640)	Standard oxygen therapy (n=640)
2	42 (6.6)	40 (6.2)
3	12 (1.9)	9 (1.4)
4	4 (0.6)	8 (1.2)
5	3 (0.5)	4 (0.6)
6	1 (0.2)	0
7	0	1 (0.2)
8	1 (0.2)	1 (0.2)
9	1 (0.2)	0
10	3 (0.5)	1 (0.2)
30	1 (0.2)	0
60	1 (0.2)	0
Mode of transport to hospital for this surgery (note: patients may use more than one), No. (%)		
Ambulance	253 (39.5)	236 (36.9)
Taxi	25 (3.9)	27 (4.2)
Public transport	26 (4.1)	30 (4.7)
Car	292 (45.6)	311 (48.6)
Other	57 (8.9)	50 (7.8)
Who accompanied patient to hospital for this surgery, No. (%)		
No-one	154 (24.4)	149 (23.4)
Spouse/partner	217 (34.3)	215 (33.8)
Other relative	112 (17.7)	118 (18.5)
Paid caregiver	13 (2.1)	17 (2.7)
Child (under 18 years)	7 (1.1)	4 (0.6)
Other	151 (23.9)	160 (25.1)
Number of adults living in patients household, No. (%)		
0	10 (1.6)	5 (0.8)
1	140 (21.9)	153 (23.9)
2	321 (50.2)	330 (51.6)
3	110 (17.2)	94 (14.7)
4	35 (5.5)	38 (5.9)

© 2026 Litton E et al. *JAMA Network Open*.

Variable	High-flow nasal therapy (n=640)	Standard oxygen therapy (n=640)
5	9 (1.4)	9 (1.4)
6	5 (0.8)	5 (0.8)
8	1 (0.2)	1 (0.2)
9	1 (0.2)	0
10	0	1 (0.2)
Number of children under 18 years living in patients household, No. (%)		
0	545 (85.2)	532 (83.1)
1	40 (6.2)	44 (6.9)
2	27 (4.2)	38 (5.9)
3	12 (1.9)	13 (2.0)
4	5 (0.8)	7 (1.1)
6	2 (0.3)	1 (0.2)
9	1 (0.2)	0
10	0	1 (0.2)
Highest level of education you completed, No. (%)		
Primary	4 (0.6)	6 (0.9)
Some Secondary	32 (5.0)	29 (4.5)
All Secondary	152 (23.8)	161 (25.2)
College	106 (16.6)	108 (16.9)
University	48 (7.5)	44 (6.9)
Post-Graduate University	24 (3.8)	24 (3.8)
Patient estimate of annual household income in the last year from all sources (before tax and including partner/spouse) for UK patients, No. (%)		
<£15,000	74 (19.5)	76 (20.2)
£15,000 – £19,999	50 (13.2)	63 (16.8)
£20,000-£29,999	65 (17.1)	64 (17.0)
£30,000-£39,999	46 (12.1)	50 (13.3)
£40,000-£59,999	56 (14.8)	52 (13.8)
£60,000+	46 (12.1)	42 (11.2)

© 2026 Litton E et al. *JAMA Network Open*.

Variable	High-flow nasal therapy (n=640)	Standard oxygen therapy (n=640)
Patient estimate of annual household income in the last year from all sources (before tax and including your partner/spouse) for Australian and New Zealand patients, No. (%)		
$499 Or Less/Wk (<$25,948/Year)	72 (27.6)	74 (28.0)
$500-$999/Wk ($26,000 - $51,948/Year)	37 (14.2)	50 (18.9)
$1,000-$1,999/Wk ($52,000-$103,948/Year)	61 (23.4)	48 (18.2)
$2,000 Or More Per Week (>$104,000/Year)	37 (14.2)	37 (14.0)
Prefer Not To Answer	54 (20.7)	55 (20.8)

© 2026 Litton E et al. *JAMA Network Open*.

eTable 6. Median Quantile Regression Between Response Variable DAH90 and Treatment Group, Adjusted for Other Selected Baseline Variables
=

**Explanatory variable**	**Effect**	**95% confidence interval**
Intercept	16.00	-1.798e+308, 1.798e+308
Treatment group (reference: standard oxygen therapy)		
HFNT	-1.83e-15	-1.832e-15, -1.832e-15
ARISCAT risk category (reference: low)		
Intermediate	-1.24e-17	-1.798e+308, 1.798e+308
High	-1.79e-15	-1.798e+308, 1.798e+308
EUROSCORE II	-6.72e-18	-6.715e-18, -6.715e-18
Gender (reference: female)		
Male	2.35e-16	2.353e-16, 2.353e-16
COPD (reference: no)		
Yes	-2.01e-15	-2.013e-15, -2.013e-15
Asthma (reference: no)		
Yes	-1.85e-15	-1.849e-15, -1.849e-15
Obesity (BMI>35) (reference: no)		
Yes	-2.10e-15	-2.098e-15, -2.098e-15
Current smoker (reference: no)		
Yes	1.51e-15	1.5137e-15, 1.5137e-15
Lower respiratory tract infection in last 4 weeks (reference: no)		
Yes	8.50e-16	8.498e-16, 8.498e-16
Age (reference: 80 years or under)		
Over 80 years	-16	-26.393, 0.0913

© 2026 Litton E et al. *JAMA Network Open*.

**Explanatory variable**	**Effect**	**95% confidence interval**
First time or redo surgery (reference: first time)		
Redo	8.99e-16	8.993e-16, 8.993e-16
Country (reference: UK)		
Australia	-16	-55.916, -0.986
New Zealand	-16	-48.923, -2.329

Due to the way COPD, asthma, current smoker and lower respiratory tract infection in the last 4 weeks were recorded in the database we cannot distinguish between 'No' answers and missing data. As this model performs a complete case analysis any missing values for COPD, asthma, current smoker and lower respiratory tract infection in the last 4 weeks will be assumed to be 'No' answers.

© 2026 Litton E et al. *JAMA Network Open*.

eTable 7. Comparison of Postextubation ROX Indexes by Treatment Group

ost- tion	High-flow nasal therapy, median	Standard oxygen therapy, median	Difference in median (95% co[illegible] interval)
	16.84	17.99	-1.19 (-[illegible]
	16.96	19.05	-1.98 (-[illegible]
	16.67	18.54	-1.73 (-[illegible]
	16.96	18.05	-1.4 ([illegible]
	19.25	19.54	-0.6 ([illegible]

eTable 8. Baseline EQ-5D-5L Questionnaire by Treatment Arm

Variable	High-flow nasal therapy (n=639)	Standard oxygen therapy (n=640)
Mobility, No. (%)		
No Problems	358 (56.0)	367 (57.3)
Slight Problems	128 (20.0)	121 (18.9)
Moderate Problems	98 (15.3)	108 (16.9)
Severe Problems	47 (7.4)	37 (5.8)
Unable To	4 (0.6)	4 (0.6)
Self-care, No. (%)		
No Problems	559 (87.5)	565 (88.3)
Slight Problems	42 (6.6)	43 (6.7)
Moderate Problems	29 (4.5)	27 (4.2)
Severe Problems	4 (0.6)	2 (0.3)
Unable To	1 (0.2)	0
Usual activities, No. (%)		
No Problems	341 (53.4)	306 (47.8)
Slight Problems	90 (14.1)	130 (20.3)
Moderate Problems	86 (13.5)	88 (13.8)
Severe Problems	36 (5.6)	33 (5.2)
Unable To	81 (12.7)	80 (12.5)
Pain/discomfort, No. (%)		
None	386 (60.4)	367 (57.3)
Slight	116 (18.1)	149 (23.3)
Moderate	100 (15.7)	92 (14.4)
Severe	31 (4.8)	27 (4.2)
Extreme	2 (0.3)	2 (0.3)
Anxiety/depression, No. (%)		
None	276 (43.2)	295 (46.1)
Slight	208 (32.5)	187 (29.2)
Moderate	105 (16.4)	98 (15.3)
Severe	25 (3.9)	31 (4.8)
Extreme	21 (3.3)	26 (4.1)
Visual analogue scale, No., median (IQR)	634, 65 (30)	637, 60 (25)

© 2026 Litton E et al. *JAMA Network Open.*

© 2026 Litton E et al. *JAMA Network Open*.

eTable 9. Discharge EQ-5D-5L Questionnaire by Treatment Arm

**Variable**	**High-flow nasal therapy (n=622)**	**Standard oxygen therapy (n=624)**
Mobility, No. (%)		
No Problems	264 (42.4)	262 (42.0)
Slight Problems	198 (31.8)	188 (30.1)
Moderate Problems	110 (17.7)	115 (18.4)
Severe Problems	20 (3.2)	34 (5.5)
Unable To	3 (0.5)	4 (0.6)
Self-care, No. (%)		
No Problems	348 (56.0)	343 (55.0)
Slight Problems	153 (24.6)	169 (27.1)
Moderate Problems	72 (11.6)	73 (11.7)
Severe Problems	15 (2.4)	12 (1.9)
Unable To	8 (1.3)	7 (1.1)
Usual activities, No. (%)		
No Problems	108 (17.4)	108 (17.3)
Slight Problems	89 (14.3)	107 (17.1)
Moderate Problems	96 (15.4)	99 (15.9)
Severe Problems	41 (6.6)	43 (6.9)
Unable To	260 (41.8)	245 (39.3)
Pain/discomfort, No. (%)		
None	124 (19.9)	132 (21.1)
Slight	256 (41.2)	247 (39.6)
Moderate	184 (29.6)	194 (31.1)
Severe	29 (4.7)	28 (4.5)
Extreme	4 (0.6)	3 (0.5)
Anxiety/depression, No. (%)		
None	349 (56.1)	365 (58.5)
Slight	149 (23.9)	139 (22.3)
Moderate	75 (12.1)	66 (10.6)
Severe	14 (2.2)	21 (3.4)
Extreme	10 (1.6)	12 (1.9)
Visual analogue scale, No., median (IQR)	593, 60 (25)	598, 60 (25)

© 2026 Litton E et al. *JAMA Network Open*.

eTable 10. Day 30 EQ-5D-5L Questionnaire by Treatment Arm

**Variable**	**High-flow nasal therapy (n=492)**	**Standard oxygen therapy (n=492)**
Mobility, No. (%)		
No Problems	269 (54.7)	279 (56.7)
Slight Problems	131 (26.6)	107 (21.8)
Moderate Problems	72 (14.6)	79 (16.1)
Severe Problems	18 (3.7)	23 (4.7)
Unable To	2 (0.4)	4 (0.8)
Self-care, No. (%)		
No Problems	357 (72.6)	353 (71.8)
Slight Problems	89 (18.1)	89 (18.1)
Moderate Problems	34 (6.9)	40 (8.1)
Severe Problems	7 (1.4)	7 (1.4)
Unable To	5 (1.0)	2 (0.4)
Usual activities, No. (%)		
No Problems	129 (26.2)	146 (29.7)
Slight Problems	173 (35.2)	129 (26.2)
Moderate Problems	99 (20.1)	112 (22.8)
Severe Problems	29 (5.9)	28 (5.7)
Unable To	62 (12.6)	76 (15.4)
Pain/discomfort, No. (%)		
None	113 (23.0)	122 (24.8)
Slight	206 (41.9)	205 (41.7)
Moderate	138 (28.1)	132 (26.8)
Severe	28 (5.7)	29 (5.9)
Extreme	6 (1.2)	3 (0.6)
Anxiety/depression, No. (%)		
None	303 (61.6)	312 (63.4)
Slight	105 (21.3)	103 (20.9)
Moderate	64 (13.0)	57 (11.6)
Severe	13 (2.6)	12 (2.4)
Extreme	6 (1.2)	7 (1.4)
Visual analogue scale, No., median (IQR)	491, 70 (30)	491, 70 (30)

© 2026 Litton E et al. *JAMA Network Open*.

eTable 11. Day 90 EQ-5D-5L Questionnaire by Treatment Arm

Variable	High-flow nasal therapy (n=513)	Standard oxygen therapy (n=511)
Mobility, No. (%)		
No Problems	314 (61.2)	316 (61.8)
Slight Problems	98 (19.1)	99 (19.4)
Moderate Problems	82 (16.0)	70 (13.7)
Severe Problems	18 (3.5)	22 (4.3)
Unable To	1 (0.2)	4 (0.8)
Self-care, No. (%)		
No Problems	427 (83.2)	433 (84.7)
Slight Problems	53 (10.3)	44 (8.6)
Moderate Problems	26 (5.1)	21 (4.1)
Severe Problems	5 (1.0)	6 (1.2)
Unable To	2 (0.4)	7 (1.4)
Usual activities, No. (%)		
No Problems	280 (54.6)	273 (53.4)
Slight Problems	132 (25.7)	132 (25.8)
Moderate Problems	64 (12.5)	64 (12.5)
Severe Problems	20 (3.9)	16 (3.1)
Unable To	17 (3.3)	26 (5.1)
Pain/discomfort, No. (%)		
None	199 (38.8)	222 (43.4)
Slight	195 (38.0)	185 (36.2)
Moderate	92 (17.9)	78 (15.3)
Severe	23 (4.5)	23 (4.5)
Extreme	4 (0.8)	3 (0.6)
Anxiety/depression, No. (%)		
None	337 (65.7)	354 (69.3)
Slight	93 (18.1)	91 (17.8)
Moderate	55 (10.7)	41 (8.0)
Severe	19 (3.7)	19 (3.7)
Extreme	9 (1.8)	5 (1.0)
Visual analogue scale, No., median (IQR)	513, 80 (27)	611, 80 (24.5)

© 2026 Litton E et al. *JAMA Network Open*.

eTable 12. Baseline Barthel Index Questionnaire by Treatment Arm

Variable	High-flow nasal therapy (n=639)	Standard oxygen therapy (n=640)
Feeding, No. (%)		
I Am Unable To Feed Myself	4 (0.6)	2 (0.3)
I Need Help Cutting, Spreading Butter Etc Or Require A Modified Diet	4 (0.6)	5 (0.8)
I Can Independently Feed Myself	624 (97.7)	628 (98.1)
Bathing, No. (%)		
I Am Dependent On Help To Bathe	21 (3.3)	21 (3.3)
I Can Independently Have A Bath Or Shower	611 (95.6)	615 (96.1)
Grooming, No. (%)		
I Need Help With Personal Care	7 (1.1)	5 (0.8)
I Am Independent To Groom My Face/Hair/Teeth/Shaving	625 (97.8)	631 (98.6)
Dressing, No. (%)		
I Am Dependent On Help To Dress	2 (0.3)	6 (0.9)
I Need Help But Can Do About Half Unaided	31 (4.8)	29 (4.5)
I Am Independent (Including Buttons, Zips, Laces, Etc) For Getting Dressed	599 (93.7)	600 (93.8)
Bowels, No. (%)		
I Am Incontinent (I Have No Bowel Control), Or Need To Be Given Enemas	3 (0.5)	3 (0.5)
I Have The Occasional Accident	23 (3.6)	18 (2.8)
I Have No Issues With My Bowel Control	604 (94.5)	615 (96.1)
Bladder, No. (%)		
I Am Incontinent (I Have No Bladder Control), Or Am Catheterized And Unable To Manage Alone	4 (0.6)	3 (0.5)
I Have The Occasional Accident	50 (7.8)	46 (7.2)
I Have No Issues With My Bladder Control	577 (90.3)	586 (91.6)
Toilet use, No. (%)		
I Am Dependent On Help To Use The Toilet	2 (0.3)	3 (0.5)
I Need Some Help, But Can Do Something Alone	7 (1.1)	8 (1.2)

© 2026 Litton E et al. *JAMA Network Open*.

**Variable**	**High-flow nasal therapy (n=639)**	**Standard oxygen therapy (n=640)**
I Am Independent (On And Off, Dressing, Wiping)	621 (97.2)	625 (97.7)
Transfers (bed to chair and back), No. (%)		
I Am Unable, No Sitting Balance	0	1 (0.2)
I Need Major Help (One Or Two People, Physical) But Can Sit	0	5 (0.8)
I Need Minor Help (Verbal Or Physical)	13 (2.0)	6 (0.9)
I Am Independent For Transfers	619 (96.9)	624 (97.5)
Mobility (on level surfaces), No. (%)		
I Am Immobile And Can Walk Less Than 50 Yards	42 (6.6)	33 (5.2)
I Am Wheelchair Independent, Including Corners, For Greater Than 50 Yards	3 (0.5)	5 (0.8)
I Can Walk With The Help Of One Person (Verbal Or Physical) For Greater Than 50 Yards	30 (4.7)	21 (3.3)
I Am Independent (But May Use An Aid, For Example Walking Stick) For Greater Than 50 Yards	549 (85.9)	565 (88.3)
Stairs, No. (%)		
I Am Unable To Use Stairs	49 (7.7)	35 (5.5)
I Need Help To Use Stairs (Verbal, Physical, Carrying Aid)	46 (7.2)	47 (7.3)
I Am Independent On Stairs	535 (83.7)	551 (86.1)

© 2026 Litton E et al. *JAMA Network Open*.

eTable 13. Discharge Barthel Index Questionnaire by Treatment Arm

Variable	High-flow nasal therapy (n=622)	Standard oxygen therapy (n=623)
Feeding, No. (%)		
I Am Unable To Feed Myself	6 (1.0)	4 (0.6)
I Need Help Cutting, Spreading Butter Etc Or Require A Modified Diet	15 (2.4)	10 (1.6)
I Can Independently Feed Myself	573 (92.1)	587 (94.2)
Bathing, No. (%)		
I Am Dependent On Help To Bathe	109 (17.5)	106 (17.0)
I Can Independently Have A Bath Or Shower	485 (78.0)	493 (79.1)
Grooming, No. (%)		
I Need Help With Personal Care	29 (4.7)	37 (5.9)
I Am Independent To Groom My Face/Hair/Teeth/Shaving	565 (90.8)	565 (90.7)
Dressing, No. (%)		
I Am Dependent On Help To Dress	19 (3.0)	19 (3.0)
I Need Help But Can Do About Half Unaided	135 (21.7)	109 (17.5)
I Am Independent (Including Buttons, Zips, Laces, Etc) For Getting Dressed	440 (70.7)	474 (76.1)
Bowels, No. (%)		
I Am Incontinent (I Have No Bowel Control), Or Need To Be Given Enemas	7 (1.1)	4 (0.6)
I Have The Occasional Accident	26 (4.2)	26 (4.2)
I Have No Issues With My Bowel Control	561 (90.2)	572 (91.8)
Bladder, No. (%)		
I Am Incontinent (I Have No Bladder Control), Or Am Catheterized And Unable To Manage Alone	7 (1.1)	6 (1.0)
I Have The Occasional Accident	30 (4.8)	41 (6.6)
I Have No Issues With My Bladder Control	557 (89.5)	555 (89.1)
Toilet use, No. (%)		
I Am Dependent On Help To Use The Toilet	11 (1.8)	13 (2.1)
I Need Some Help, But Can Do Something Alone	18 (2.9)	16 (2.6)

© 2026 Litton E et al. *JAMA Network Open*.

Variable	High-flow nasal therapy (n=622)	Standard oxygen therapy (n=623)
I Am Independent (On And Off, Dressing, Wiping)	565 (90.8)	572 (91.8)
Transfers (bed to chair and back), No. (%)		
I Am Unable, No Sitting Balance	3 (0.5)	0
I Need Major Help (One Or Two People, Physical) But Can Sit	6 (1.0)	11 (1.8)
I Need Minor Help (Verbal Or Physical)	44 (7.1)	37 (5.9)
I Am Independent For Transfers	541 (87.0)	553 (88.8)
Mobility (on level surfaces), No. (%)		
I Am Immobile And Can Walk Less Than 50 Yards	49 (7.9)	63 (10.1)
I Am Wheelchair Independent, Including Corners, For Greater Than 50 Yards	6 (1.0)	4 (0.6)
I Can Walk With The Help Of One Person (Verbal Or Physical) For Greater Than 50 Yards	38 (6.1)	43 (6.9)
I Am Independent (But May Use An Aid, For Example Walking Stick) For Greater Than 50 Yards	494 (79.4)	484 (77.7)
Stairs, No. (%)		
I Am Unable To Use Stairs	65 (10.4)	65 (10.4)
I Need Help To Use Stairs (Verbal, Physical, Carrying Aid)	87 (14.0)	96 (15.4)
I Am Independent On Stairs	410 (65.9)	416 (66.8)

© 2026 Litton E et al. *JAMA Network Open*.

eTable 14. Day 30 Barthel Index Questionnaire by Treatment Arm

Variable	High-flow nasal therapy (n=492)	Standard oxygen therapy (n=493)
Feeding, No. (%)		
I Am Unable To Feed Myself	8 (1.6)	3 (0.6)
I Need Help Cutting, Spreading Butter Etc Or Require A Modified Diet	8 (1.6)	6 (1.2)
I Can Independently Feed Myself	474 (96.3)	482 (97.8)
Bathing, No. (%)		
I Am Dependent On Help To Bathe	65 (13.2)	54 (10.9)
I Can Independently Have A Bath Or Shower	426 (86.6)	433 (87.8)
Grooming, No. (%)		
I Need Help With Personal Care	24 (4.9)	19 (3.9)
I Am Independent To Groom My Face/Hair/Teeth/Shaving	467 (94.9)	470 (95.3)
Dressing, No. (%)		
I Am Dependent On Help To Dress	12 (2.4)	12 (2.4)
I Need Help But Can Do About Half Unaided	65 (13.2)	56 (11.4)
I Am Independent (Including Buttons, Zips, Laces, Etc) For Getting Dressed	409 (83.1)	421 (85.4)
Bowels, No. (%)		
I Am Incontinent (I Have No Bowel Control), Or Need To Be Given Enemas	4 (0.8)	1 (0.2)
I Have The Occasional Accident	23 (4.7)	15 (3.0)
I Have No Issues With My Bowel Control	463 (94.1)	472 (95.7)
Bladder		
I Am Incontinent (I Have No Bladder Control), Or Am Catheterized And Unable To Manage Alone	6 (1.2)	8 (1.6)
I Have The Occasional Accident	31 (6.3)	23 (4.7)
I Have No Issues With My Bladder Control	453 (92.1)	459 (93.1)
Toilet use, No. (%)		
I Am Dependent On Help To Use The Toilet	5 (1.0)	3 (0.6)
I Need Some Help, But Can Do Something Alone	15 (3.0)	8 (1.6)

© 2026 Litton E et al. *JAMA Network Open*.

Variable	High-flow nasal therapy (n=492)	Standard oxygen therapy (n=493)
I Am Independent (On And Off, Dressing, Wiping)	469 (95.3)	478 (97.0)
Transfers (bed to chair and back), No. (%)		
I Am Unable, No Sitting Balance	1 (0.2)	1 (0.2)
I Need Major Help (One Or Two People, Physical) But Can Sit	2 (0.4)	4 (0.8)
I Need Minor Help (Verbal Or Physical)	30 (6.1)	34 (6.9)
I Am Independent For Transfers	456 (92.7)	453 (91.9)
Mobility (on level surfaces), No. (%)		
I Am Immobile And Can Walk Less Than 50 Yards	33 (6.7)	38 (7.7)
I Am Wheelchair Independent, Including Corners, For Greater Than 50 Yards	2 (0.4)	5 (1.0)
I Can Walk With The Help Of One Person (Verbal Or Physical) For Greater Than 50 Yards	40 (8.1)	46 (9.3)
I Am Independent (But May Use An Aid, For Example Walking Stick) For Greater Than 50 Yards	412 (83.7)	397 (80.5)
Stairs, No. (%)		
I Am Unable To Use Stairs	28 (5.7)	32 (6.5)
I Need Help To Use Stairs (Verbal, Physical, Carrying Aid)	41 (8.3)	40 (8.1)
I Am Independent On Stairs	400 (81.3)	407 (82.6)

© 2026 Litton E et al. *JAMA Network Open*.

eTable 15. Day 90 Barthel Index Questionnaire by Treatment Arm

Variable	High-flow nasal therapy (n=513)	Standard oxygen therapy (n=513)
Feeding, No. (%)		
I Am Unable To Feed Myself	8 (1.6)	7 (1.4)
I Need Help Cutting, Spreading Butter Etc Or Require A Modified Diet	6 (1.2)	2 (0.4)
I Can Independently Feed Myself	497 (96.9)	503 (98.0)
Bathing, No. (%)		
I Am Dependent On Help To Bathe	38 (7.4)	29 (5.7)
I Can Independently Have A Bath Or Shower	473 (92.2)	481 (93.8)
Grooming, No. (%)		
I Need Help With Personal Care	19 (3.7)	12 (2.3)
I Am Independent To Groom My Face/Hair/Teeth/Shaving	490 (95.5)	499 (97.3)
Dressing, No. (%)		
I Am Dependent On Help To Dress	5 (1.0)	4 (0.8)
I Need Help But Can Do About Half Unaided	43 (8.4)	39 (7.6)
I Am Independent (Including Buttons, Zips, Laces, Etc) For Getting Dressed	457 (89.1)	469 (91.4)
Bowels, No. (%)		
I Am Incontinent (I Have No Bowel Control), Or Need To Be Given Enemas	0	2 (0.4)
I Have The Occasional Accident	28 (5.5)	15 (2.9)
I Have No Issues With My Bowel Control	482 (94.0)	496 (96.7)
Bladder, No. (%)		
I Am Incontinent (I Have No Bladder Control), Or Am Catheterized And Unable To Manage Alone	7 (1.4)	5 (1.0)
I Have The Occasional Accident	32 (6.2)	37 (7.2)
I Have No Issues With My Bladder Control	471 (91.8)	470 (91.6)
Toilet use, No. (%)		
I Am Dependent On Help To Use The Toilet	6 (1.2)	4 (0.8)

© 2026 Litton E et al. *JAMA Network Open*.

Variable	High-flow nasal therapy (n=513)	Standard oxygen therapy (n=513)
I Need Some Help, But Can Do Something Alone	10 (1.9)	5 (1.0)
I Am Independent (On And Off, Dressing, Wiping)	493 (96.1)	500 (97.5)
Transfers (bed to chair and back), No. (%)		
I Am Unable, No Sitting Balance	0	1 (0.2)
I Need Major Help (One Or Two People, Physical) But Can Sit	6 (1.2)	3 (0.6)
I Need Minor Help (Verbal Or Physical)	20 (3.9)	21 (4.1)
I Am Independent For Transfers	482 (94.0)	486 (94.7)
Mobility (on level surfaces), No. (%)		
I Am Immobile And Can Walk Less Than 50 Yards	21 (4.1)	32 (6.2)
I Am Wheelchair Independent, Including Corners, For Greater Than 50 Yards	2 (0.4)	4 (0.8)
I Can Walk With The Help Of One Person (Verbal Or Physical) For Greater Than 50 Yards	32 (6.2)	19 (3.7)
I Am Independent (But May Use An Aid, For Example Walking Stick) For Greater Than 50 Yards	452 (88.1)	451 (87.9)
Stairs, No. (%)		
I Am Unable To Use Stairs	17 (3.3)	23 (4.5)
I Need Help To Use Stairs (Verbal, Physical, Carrying Aid)	39 (7.6)	35 (6.8)
I Am Independent On Stairs	449 (87.5)	451 (87.9)

© 2026 Litton E et al. *JAMA Network Open*.

© 2026 Litton E et al. *JAMA Network Open*.

eTable 16. Barthel Index Questionnaire at Baseline, Discharge, Day 30 and Day 90, Ignoring Treatment Arm

**Barthel index domain**	**Baseline (n=1279)**	**Discharge (n=1245)**	**Day 30 (n=985)**	**Day 90 (n=1026)**
Feeding, No. (%)				
I Am Unable To Feed Myself	6 (0.5)	10 (0.8)	11 (1.1)	15 (1.5)
I Need Help Cutting, Spreading Butter Etc Or Require A Modified Diet	9 (0.7)	25 (2.0)	14 (1.4)	8 (0.8)
I Can Independently Feed Myself	1252 (97.9)	1160 (93.2)	956 (97.1)	1000 (97.5)
Bathing, No. (%)				
I Am Dependent On Help To Bathe	42 (3.3)	215 (17.3)	119 (12.1)	67 (6.5)
I Can Independently Have A Bath Or Shower	1226 (95.9)	978 (78.5)	859 (87.2)	954 (93.0)
Grooming, No. (%)				
I Need Help With Personal Care	12 (0.9)	66 (5.3)	43 (4.4)	31 (3.0)
I Am Independent To Groom My Face/Hair/Teeth/Shaving	1256 (98.2)	1130 (90.8)	937 (95.1)	989 (96.4)
Dressing, No. (%)				
I Am Dependent On Help To Dress	8 (0.6)	38 (3.0)	24 (2.4)	9 (0.9)
I Need Help But Can Do About Half Unaided	60 (4.7)	244 (19.6)	121 (12.3)	82 (8.0)
I Am Independent (Including Buttons, Zips, Laces, Etc) For Getting Dressed	1199 (93.8)	914 (73.4)	830 (84.3)	926 (90.2)
Bowels, No. (%)				
I Am Incontinent (I Have No Bowel Control), Or Need To Be Given Enemas	6 (0.5)	11 (0.9)	5 (0.5)	2 (0.2)
I Have The Occasional Accident	41 (3.2)	52 (4.2)	38 (3.9)	43 (4.2)
I Have No Issues With My Bowel Control	1219 (95.3)	1133 (91.0)	935 (94.9)	978 (95.3)
Bladder, No. (%)				
I Am Incontinent (I Have No Bladder Control), Or Am Catheterized And Unable To Manage Alone	7 (0.6)	13 (1.0)	14 (1.4)	12 (1.2)
I Have The Occasional Accident	96 (7.5)	71 (5.7)	54 (5.5)	69 (6.7)
I Have No Issues With My Bladder Control	1163 (90.9)	1112 (89.3)	912 (92.6)	941 (91.7)
Toilet use, No. (%)				
I Am Dependent On Help To Use The Toilet	5 (0.4)	24 (1.9)	8 (0.8)	10 (1.0)
I Need Some Help, But Can Do Something Alone	15 (1.2)	34 (2.7)	23 (2.3)	15 (1.5)

© 2026 Litton E et al. *JAMA Network Open*.

Barthel index domain	Baseline (n=1279)	Discharge (n=1245)	Day 30 (n=985)	Day 90 (n=1026)
I Am Independent (On And Off, Dressing, Wiping)	1246 (97.4)	1137 (91.3)	947 (96.1)	993 (96.8)
Transfers (bed to chair and back), No. (%)				
I Am Unable, No Sitting Balance	1 (0.1)	3 (0.2)	2 (0.2)	1 (0.1)
I Need Major Help (One Or Two People, Physical) But Can Sit	5 (0.4)	17 (1.4)	6 (0.6)	9 (0.9)
I Need Minor Help (Verbal Or Physical)	19 (1.5)	81 (6.5)	64 (6.5)	41 (4.0)
I Am Independent For Transfers	1243 (97.2)	1094 (87.9)	909 (92.3)	968 (94.3)
Mobility (on level surfaces), No. (%)				
I Am Immobile And Can Walk Less Than 50 Yards	75 (5.9)	112 (9.0)	71 (7.2)	53 (5.2)
I Am Wheelchair Independent, Including Corners, For Greater Than 50 Yards	8 (0.6)	10 (0.8)	7 (0.7)	6 (0.6)
I Can Walk With The Help Of One Person (Verbal Or Physical) For Greater Than 50 Yards	51 (4.0)	81 (6.5)	86 (8.7)	51 (5.0)
I Am Independent (But May Use An Aid, For Example Walking Stick) For Greater Than 50 Yards	1114 (87.1)	978 (78.5)	809 (82.1)	903 (88.0)
Stairs, No. (%)				
I Am Unable To Use Stairs	84 (6.6)	130 (10.4%)	60 (6.1)	40 (3.9)
I Need Help To Use Stairs (Verbal, Physical, Carrying Aid)	93 (7.3)	183 (14.7%)	81 (8.2)	74 (7.2)
I Am Independent On Stairs	1086 (84.9)	826 (66.3%)	807 (81.9)	900 (87.7)

eTable 17. Discharge Participant and Family Resource Use Questionnaire by Treatment Arm

Variable	High-flow nasal therapy (n=640)	Standard oxygen therapy (n=640)
Mode of transport used to leave hospital? (note: patients may use more than one), No. (%)		
Ambulance	46 (7.2)	52 (8.1)
Taxi	28 (4.4)	40 (6.2)
Public transport	7 (1.1)	2 (0.3)
Car	475 (74.2)	471 (73.6)

© 2026 Litton E et al. *JAMA Network Open*.

Variable	High-flow nasal therapy (n=640)	Standard oxygen therapy (n=640)
Other	45 (7.0)	46 (7.2)
Who will accompany patient when they leave the hospital, No. (%)		
No-one	111 (17.3)	103 (16.1)
Spouse/partner	296 (46.2)	290 (45.3)
Other relative	266 (41.6)	235 (36.7)
Paid caregiver	46 (7.2)	36 (5.6)
Child (under 18 years)	44 (6.9)	34 (5.3)
Other	102 (15.9)	119 (18.6)
Patients who had visitors while in hospital, No. (%)	520 (81.2)	520 (81.2)
Thinking of your main visitor, how many times did they visit between surgery and discharge?, No. (%)		
0	1 (0.2)	0
1	51 (8.0)	48 (7.5)
2	56 (8.8)	42 (6.6)
3	66 (10.3)	54 (8.4)
4	45 (7.0)	57 (8.9)
5	73 (11.4)	75 (11.7)
6	51 (8.0)	56 (8.8)
7	41 (6.4)	37 (5.8)
8	26 (4.1)	32 (5.0)
9	17 (2.7)	12 (1.9)
10	16 (2.5)	28 (4.4)
11	11 (1.7)	5 (0.8)
12	12 (1.9)	10 (1.6)
13	6 (0.9)	4 (0.6)
14	7 (1.1)	15 (2.3)
15	2 (0.3)	9 (1.4)
16	4 (0.6)	4 (0.6)
17	0	4 (0.6)
18	7 (1.1)	2 (0.3)
19	1 (0.2)	4 (0.6)

© 2026 Litton E et al. *JAMA Network Open*.

Variable	High-flow nasal therapy (n=640)	Standard oxygen therapy (n=640)
20	4 (0.6)	4 (0.6)
21	5 (0.8)	3 (0.5)
22	0	1 (0.2)
23	2 (0.3)	0
24	0	3 (0.5)
25	2 (0.3)	5 (0.8)
26	0	2 (0.3)
27	1 (0.2)	0
29	1 (0.2)	0
30	1 (0.2)	1 (0.2)
32	2 (0.3)	0
38	1 (0.2)	0
44	1 (0.2)	0
61	1 (0.2)	0
74	1 (0.2%)	0
Thinking of your main visitor, how long did they tend to spend with you at each visit (hours)? No., median (IQR)	519, 2 (2)	519, 2 (3)
Thinking of your main visitor, how long on average did they take travelling to hospital (hours)? No., median (IQR)	514, 0.71 (0.67)	515, 0.75 (0.88)
Thinking of your main visitor, how did they tend to travel to visit you? (note more than one answer may be selected), No. (%)		
Taxi	22 (3.4)	15 (2.3)
Public transport	79 (12.3)	89 (13.9)
Car	396 (61.9)	403 (63.0)
Other	37 (5.8)	27 (4.2)
Thinking of your main visitor, if they had not visited you in hospital, what would they normally have been doing at the time they tended to visit you?, No. (%)		
Paid Work	245 (38.3)	244 (38.1)
Voluntary Work	3 (0.5)	7 (1.1)
Caring For Child Or Other Relative	37 (5.8)	46 (7.2)

© 2026 Litton E et al. *JAMA Network Open*.

Variable	High-flow nasal therapy (n=640)	Standard oxygen therapy (n=640)
Housework	105 (16.4)	100 (15.6)
Leisure Activities	99 (15.5)	92 (14.4)
Studying	4 (0.6)	6 (0.9)
Other	22 (3.4)	21 (3.3)
Thinking about yourself, what main activity would you have been doing if you had not been in hospital for surgery?, No. (%)		
Paid Work (Full-Time)	207 (32.3)	192 (30.0)
Paid Work (Part-Time)	46 (7.2)	38 (5.9)
Voluntary Work (Full-Time)	1 (0.2)	6 (0.9)
Voluntary Work (Part-Time)	10 (1.6)	9 (1.4)
Caring For Child Or Other Relative	18 (2.8)	29 (4.5)
Housework	77 (12.0)	81 (12.7)
Leisure Activities	202 (31.6)	207 (32.3)
Studying	4 (0.6)	0
Other	30 (4.7)	41 (6.4)
Have children under 18 years or other dependents who you had to make care arrangements for in order to be able to attend hospital for surgery, No. (%)	35 (5.5)	46 (7.2)

© 2026 Litton E et al. *JAMA Network Open*.

eTable 18. Discharge Destination by Treatment Arm

Variable	High-flow nasal therapy (n=603)	Standard oxygen therapy (n=617)
Discharge destination, No. (%)		
Home	548 (90.9)	563 (91.2%)
Hospital	20 (3.3)	29 (4.7%)
Residential Home	1 (0.2)	1 (0.2%)
Nursing Home	1 (0.2)	0 (0.0%)
Relative's Home	0 (0.0%)	0 (0.0%)
Other	21 (3.5%)	14 (2.3%)
Missing or deceased	12 (2.0%)	10 (1.6%)

© 2026 Litton E et al. *JAMA Network Open*.

eTable 19. Frequency and Duration (Days) of Nonhospital Stays Away From Home Following Discharge From Index Hospital Admission by Treatment Arm

**Variable**	**High-flow nasal therapy (n=598)**	**Standard oxygen therapy (n=604)**
Number of patients who spent any time away from home in a non-hospital setting, No. (%)	88 (14.7)	74 (12.2)
Number of patients who spent time in a relative or friend's home, No. (%)	57 (9.5)	47 (7.8)
Number of patients who spent time in a nursing home, No. (%)	3 (0.5)	0
Number of patients who spent time in a residential home, No. (%)	1 (0.2)	1 (0.2)
Number of patients who spent time in respite care, No. (%)	8 (1.3)	9 (1.5)
Number of patients who spent time in a location listed as "other", No. (%)	24 (4.0)	28 (4.6)
Number of stays away from home per patient, median (IQR)	0 (0)	0 (0)
Number of stays in a relative or friends home, median (IQR)	0 (0)	0 (0)
Number of stays in a nursing home, median (IQR)	0 (0)	0 (0)
Number of stays in a residential home, median (IQR)	0 (0)	0 (0)
Number of stays in respite care, median (IQR)	0 (0)	0 (0)
Number of stays in a location listed as "other", median (IQR)	0 (0)	0 (0)
Duration of individual stays away from home (days), No., median (IQR)	125, 9 (25)	135, 6 (19)
Duration of individual stays with relative or friend (days), No., median (IQR)	76, 17 (34.5)	77, 7 (23)
Duration of individual stays in a nursing home (days), No., median (IQR)	3, 22 (1.5)	0, NA (NA)
Duration of individual stays in a residential home (days), No., median (IQR)	1, 9 (0)	1, 21 (0)
Duration of individual stays in respite care (days), No., median (IQR)	8, 7 (2)	9, 7 (3)
Duration of individual stays in a location listed as "other" (days), No., median (IQR)	37, 3 (6)	48, 5.5 (12)
Total duration of stays away from home (days), No., median (IQR)	88, 16 (53)	74, 18 (38.25)
Total duration of time spent with a relative or friend (days), No., median (IQR)	57, 28 (71)	47, 25 (48)
Total duration of time spent in a nursing home (days), No., median (IQR)	3, 22 (1.5)	0, NA (NA)
Total duration of time spent in a residential home (days), No., median (IQR)	1, 9 (0)	1, 21 (0)
Total duration of time spent in respite care (days), No., median (IQR)	8, 7 (2)	9, 7 (3)

© 2026 Litton E et al. *JAMA Network Open*.

Variable	High-flow nasal therapy (n=598)	Standard oxygen therapy (n=604)
Total duration of time spent in a location listed as “other” (days), No., median (IQR)	23, 7 (12.5)	28, 14 (22.5)

© 2026 Litton E et al. *JAMA Network Open*.

eTable 20. Incidence Rate of Serious Adverse Events of Special Interest by Treatment Arm

SAE of special interest	High-flow nasal therapy (n=640)	Standard oxygen therapy (n=640)
No. of serious adverse events of special interest	1044	1037
Stroke, No. (%)	2 (0.2)	8 (0.8)
Sepsis, No. (%)	16 (1.5)	11 (1.1)
Myocardial infarction, No. (%)	3 (0.3)	7 (0.7)
Acute kidney injury, No. (%)	18 (1.7)	24 (2.3)
Other condition, No. (%)	336 (32.2)	336 (32.4)

A patient may be represented in this table more than once if they had multiple adverse events of special interest, or not at all if they experienced no adverse events of special interest

© 2026 Litton E et al. *JAMA Network Open*.

eTable 21. Summary of Adverse Events (Excludes Serious Adverse Events) By Treatment Arm

Variable	High-flow nasal therapy (n=640)	Standard oxygen therapy (n=640)
Outcome, No. (%)		
Recovered/Resolved	600 (79.0)	660 (77.7)
Recovered With Sequelae	4 (0.5)	3 (0.3)
Recovering/Resolving	98 (12.9)	104 (12.2)
Ongoing	57 (7.5)	82 (9.7)
Severity, No. (%)		
Mild	577 (76.0)	676 (79.6)
Moderate	178 (23.4)	166 (19.6)
Severe	4 (0.5)	7 (0.8)
Is the event related to the study device, No. (%)		
Not Related	746 (98.3)	840 (98.9)
Unlikely To Be Related	12 (1.6)	9 (1.1)
Possibly Related	1 (0.1)	0
Probably Related	0	0
Definitely Related	0	0

© 2026 Litton E et al. *JAMA Network Open*.

eTable 22. Summary of Serious Adverse Events by Treatment Arm

Variable	High-flow nasal therapy (n=640)	Standard oxygen therapy (n=640)
Outcome, No. (%)		
Recovered/Resolved	216 (86.1)	200 (81.6)
Recovered With Sequelae	3 (1.2)	4 (1.6)
Recovering/Resolving	20 (8.0)	19 (7.8)
Ongoing	12 (4.8)	22 (9.0)
Severity, No. (%)		
Mild	115 (45.8)	115 (46.9)
Moderate	110 (43.8)	95 (38.8)
Severe	26 (10.4)	35 (14.3)
Is the event related to the study device?, No. (%)		
Not Related	250 (99.6)	243 (99.2)
Unlikely To Be Related	1 (0.4)	2 (0.8)
Possibly Related	0	0
Probably Related	0	0
Definitely Related	0	0
Was the SAE expected?, No. (%)		
Yes	236 (94.0)	238 (97.1)
Seriousness, No. (%)		
Death	7 (2.8)	2 (0.8)
Life Threatening	6 (2.4)	7 (2.9)
Hospitalisation	156 (62.1)	166 (67.8)
Hospitalisation - Prolonged	73 (29.1)	73 (29.8)
Persistent Or Significant Disability Or Incapacity	2 (0.8)	1 (0.4)
Congenital Anomaly Or Birth Defect	0	0
Important Medical Event	7 (2.8)	2 (0.8)

© 2026 Litton E et al. *JAMA Network Open*.

eFigure 2. Adherence With Randomized Therapy, Time on Randomized and Nonrandomized Treatment Arms for Patients Randomized to Nasal High-Flow Nasal Oxygen Therapy (NHFOT) or Standard Oxygen Therapy (SOT)

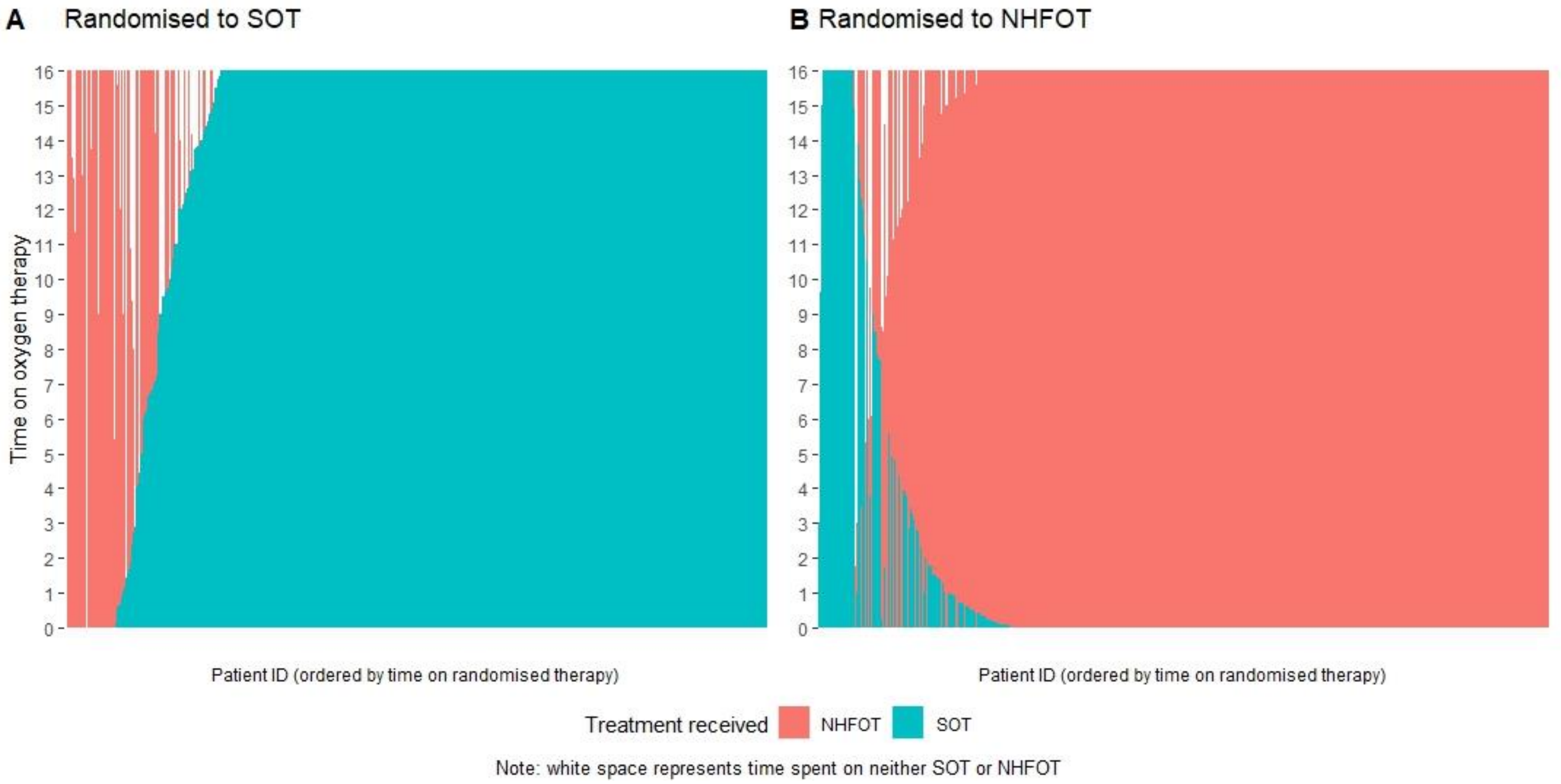

© 2026 Litton E et al. *JAMA Network Open*.
