# Supplementary material for: High-Flow Nasal Oxygen Therapy After Cardiac Surgery: A Randomized Clinical Trial
Source: JAMA Netw Open. 2026 Apr 8;9(4):e265447. doi: 10.1001/jamanetworkopen.2026.5447 (PMC13063085; doi:10.1001/jamanetworkopen.2026.5447)
Supplement: Supplement 3. — Nonauthor Collaborators [file jamanetwopen-e265447-s003.pdf]

Supplemental Online Content: Nonauthor Collaborators
*First name, last name, and suffix (if applicable) are required and will appear in PubMed.

*Group Name(s): The Nasal High-Flow Oxygen Therapy After Cardiac Surgery (NOTACS) Investigators							
***First Name and Middle Initial(s)**	***Last Name**	***Suffix (eg, Jr, III)**	Academic Degrees	Institution	Location (city, state/province, country)	Role or Contribution, eg, chair, principal investigator	Group (if more than 1 Group listed in the byline) and/or Subgroup (eg, Steering Committee)
Leena	Chaudhari			Glenfield Hospital, University Hospitals of Leicester NHS Trust	Leicester, Leicestershire, UK	Co-Investigator	
Hakeem Yusuff	Yusuff			Glenfield Hospital, University Hospitals of Leicester NHS Trust	Leicester, Leicestershire, UK	Co-Investigator	
Mahfuji	Ahmed			Glenfield Hospital, University Hospitals of Leicester NHS Trust	Leicester, Leicestershire, UK	Research Nurse	
Faszai	Chikwanha			Glenfield Hospital, University Hospitals of Leicester NHS Trust	Leicester, Leicestershire, UK	Research Nurse	
Navneet	Ghuhan			Glenfield Hospital, University Hospitals of Leicester NHS Trust	Leicester, Leicestershire, UK	Research Nurse	
Beverly	Hargadon			Glenfield Hospital, University Hospitals of Leicester NHS Trust	Leicester, Leicestershire, UK	Research Nurse	
Alexander	Parsons			Glenfield Hospital, University Hospitals of Leicester NHS Trust	Leicester, Leicestershire, UK	Research Nurse	
Malvi	Raval			Glenfield Hospital, University Hospitals of Leicester NHS Trust	Leicester, Leicestershire, UK	Research Nurse	
Hardeep	Aujla			Glenfield Hospital, University Hospitals of Leicester NHS Trust	Leicester, Leicestershire, UK	R&D Manager (contracts)	
Louise	Hanson			Glenfield Hospital, University Hospitals of Leicester NHS Trust	Leicester, Leicestershire, UK	R&D Manager (contracts)	
Phillipa	Sum			Glenfield Hospital, University Hospitals of Leicester NHS Trust	Leicester, Leicestershire, UK	R&D Manager (contracts)	
Jonathan	Brand			James Cook Hospital, South Tees Hopsitals NHS Foundation Trust	Middlesbrough, North Yorkshire, UK	Co-Investigator	
Anthony	Donnelly			James Cook Hospital, South Tees Hopsitals NHS Foundation Trust	Middlesbrough, North Yorkshire, UK	Research Coordinator	
Rachel	Harrison			James Cook Hospital, South Tees Hopsitals NHS Foundation Trust	Middlesbrough, North Yorkshire, UK	Research Administrator	
Rachel	Kipling			James Cook Hospital, South Tees Hopsitals NHS Foundation Trust	Middlesbrough, North Yorkshire, UK	Research Nurse	
Carmen	Neave			James Cook Hospital, South Tees Hopsitals NHS Foundation Trust	Middlesbrough, North Yorkshire, UK	Research Nurse	
Holly	Patterson			James Cook Hospital, South Tees Hopsitals NHS Foundation Trust	Middlesbrough, North Yorkshire, UK	Research Nurse	
Sue	Metcalfe			James Cook Hospital, South Tees Hopsitals NHS Foundation Trust	Middlesbrough, North Yorkshire, UK	R&D Manager (contracts)	

Supplemental Online Content: Nonauthor Collaborators
*First name, last name, and suffix (if applicable) are required and will appear in PubMed.

***First Name and Middle Initial(s)**	***Last Name**	***Suffix (eg, Jr, III)**	Academic Degrees	Institution	Location (city, state/province, country)	Role or Contribution, eg, chair, principal investigator	Group (if more than 1 Group listed in the byline) and/or Subgroup (eg, Steering Committee)
Rachel	Taylor			James Cook Hospital, South Tees Hopsitals NHS Foundation Trust	Middlesbrough, North Yorkshire, UK	R&D Manager (contracts)	
Laura	Dymore-Brown			King's College Hospital, King's College Hospital NHS Foundation Trust	London, UK	Co-Investigator	
Daveena	Meeks			King's College Hospital, King's College Hospital NHS Foundation Trust	London, UK	Associate Principal Investigator	
Anna	Broderick			King's College Hospital, King's College Hospital NHS Foundation Trust	London, UK	Research Nurse	
Emma	Clarey			King's College Hospital, King's College Hospital NHS Foundation Trust	London, UK	Research Nurse	
Fatemah	Karami			King's College Hospital, King's College Hospital NHS Foundation Trust	London, UK	Research Nurse	
Harriet	Noble			King's College Hospital, King's College Hospital NHS Foundation Trust	London, UK	Research Nurse	
Kevin	O'Reilly			King's College Hospital, King's College Hospital NHS Foundation Trust	London, UK	Research Nurse	
Sian	Saha			King's College Hospital, King's College Hospital NHS Foundation Trust	London, UK	Research Nurse	
Caitlin	Spooner			King's College Hospital, King's College Hospital NHS Foundation Trust	London, UK	Research Nurse	
Kirsty	Hedditch			King's College Hospital, King's College Hospital NHS Foundation Trust	London, UK	R&D Manager (contracts)	
Jasmine	Palmer			King's College Hospital, King's College Hospital NHS Foundation Trust	London, UK	R&D Manager (contracts)	
Phillip	Howells			Queen Elizabeth Hospital, University Hospitals Birmingham	Birmingham, West Midlands, UK	Co-Principal Investigator	
Rebecca	Boot			Queen Elizabeth Hospital, University Hospitals Birmingham	Birmingham, West Midlands, UK	Associate Principal Investigator	
Sophia	Beddows			Queen Elizabeth Hospital, University Hospitals Birmingham	Birmingham, West Midlands, UK	Research Nurse	
Amy	Clark			Queen Elizabeth Hospital, University Hospitals Birmingham	Birmingham, West Midlands, UK	Research Nurse	
Caroline	Dooley			Queen Elizabeth Hospital, University Hospitals Birmingham	Birmingham, West Midlands, UK	Research Nurse	
Karen	Ellis			Queen Elizabeth Hospital, University Hospitals Birmingham	Birmingham, West Midlands, UK	Research Nurse	

*First name, last name, and suffix (if applicable) are required and will appear in PubMed.

***First Name and Middle Initial(s)**	***Last Name**	***Suffix (eg, Jr, III)**	Academic Degrees	Institution	Location (city, state/province, country)	Role or Contribution, eg, chair, principal investigator	Group (if more than 1 Group listed in the byline) and/or Subgroup (eg, Steering Committee)
Lisa	Moody			Queen Elizabeth Hospital, University Hospitals Birmingham	Birmingham, West Midlands, UK	Research Administrator	
Christopher	Sheridan			Queen Elizabeth Hospital, University Hospitals Birmingham	Birmingham, West Midlands, UK	Research Nurse	
Elaine	Spruce			Queen Elizabeth Hospital, University Hospitals Birmingham	Birmingham, West Midlands, UK	Research Nurse	
Sumayyah	Ul-Rahman			Queen Elizabeth Hospital, University Hospitals Birmingham	Birmingham, West Midlands, UK	Data Manager	
Hazel	Smith			Queen Elizabeth Hospital, University Hospitals Birmingham	Birmingham, West Midlands, UK	R&D Manager (contracts)	
Zohreh	Farzad			Royal Brompton Hospital, Guy's and St Thomas' NHS Foundation Trust	London, UK	Research Nurse	
Kevin	Kirby			Royal Brompton Hospital, Guy's and St Thomas' NHS Foundation Trust	London, UK	Research Nurse	
Elizabeth	Mittoo			Royal Brompton Hospital, Guy's and St Thomas' NHS Foundation Trust	London, UK	Research Nurse	
Annashyl	West			Royal Brompton Hospital, Guy's and St Thomas' NHS Foundation Trust	London, UK	Research Nurse	
Orla	Worn			Royal Brompton Hospital, Guy's and St Thomas' NHS Foundation Trust	London, UK	Research Nurse	
Susana	Pina			Royal Brompton Hospital, Guy's and St Thomas' NHS Foundation Trust	London, UK	R&D Manager (contracts)	
Karen	Fitzjohn			Royal Papworth Hospital, Royal Papworth Hospital NHS Foundation Trust	Cambridge, Cambridgeshire, UK	Research Coordinator	
Elizabeth	Hodge			Royal Papworth Hospital, Royal Papworth Hospital NHS Foundation Trust	Cambridge, Cambridgeshire, UK	Research Coordinator	
Helen	Holcombe			Royal Papworth Hospital, Royal Papworth Hospital NHS Foundation Trust	Cambridge, Cambridgeshire, UK	Research Assisstant	
Jennifer	Mackie			Royal Papworth Hospital, Royal Papworth Hospital NHS Foundation Trust	Cambridge, Cambridgeshire, UK	Research Coordinator	
Georgia	Moule			Royal Papworth Hospital, Royal Papworth Hospital NHS Foundation Trust	Cambridge, Cambridgeshire, UK	Research Coordinator	
Jamie	Pack			Royal Papworth Hospital, Royal Papworth Hospital NHS Foundation Trust	Cambridge, Cambridgeshire, UK	Research Coordinator	
Carmen	Pearn			Royal Papworth Hospital, Royal Papworth Hospital NHS Foundation Trust	Cambridge, Cambridgeshire, UK	Research Coordinator	

Supplemental Online Content: Nonauthor Collaborators
*First name, last name, and suffix (if applicable) are required and will appear in PubMed.

***First Name and Middle Initial(s)**	***Last Name**	***Suffix (eg, Jr, III)**	Academic Degrees	Institution	Location (city, state/province, country)	Role or Contribution, eg, chair, principal investigator	Group (if more than 1 Group listed in the byline) and/or Subgroup (eg, Steering Committee)
Michelle	Austin			Royal Papworth Hospital, Royal Papworth Hospital NHS Foundation Trust	Cambridge, Cambridgeshire, UK	Data Manager	
Sarah	Dennis			Royal Papworth Hospital, Royal Papworth Hospital NHS Foundation Trust	Cambridge, Cambridgeshire, UK	Data Assisstant	
Katie	Hodge			Royal Papworth Hospital, Royal Papworth Hospital NHS Foundation Trust	Cambridge, Cambridgeshire, UK	Data Assisstant	
Khross	Malgapo			Royal Papworth Hospital, Royal Papworth Hospital NHS Foundation Trust	Cambridge, Cambridgeshire, UK	Data Assisstant	
Gayathri	Namasivayah			Royal Papworth Hospital, Royal Papworth Hospital NHS Foundation Trust	Cambridge, Cambridgeshire, UK	Data Assisstant	
Rosemary	Thorton			Royal Papworth Hospital, Royal Papworth Hospital NHS Foundation Trust	Cambridge, Cambridgeshire, UK	Accountant	
Georgina	Arnold			Royal Papworth Hospital, Royal Papworth Hospital NHS Foundation Trust	Cambridge, Cambridgeshire, UK	Trial Administrator	
Rachel	Kingstrom			Royal Papworth Hospital, Royal Papworth Hospital NHS Foundation Trust	Cambridge, Cambridgeshire, UK	Trial Administrator	
Lucie	Garner			Royal Papworth Hospital, Royal Papworth Hospital NHS Foundation Trust	Cambridge, Cambridgeshire, UK	R&D Manager (contracts)	
Tariq	Ali			St Thomas' Hospital, Guy's and St Thomas' NHS Foundation Trust	London, UK	Co-Investigator	
Maeve	Henry			St Thomas' Hospital, Guy's and St Thomas' NHS Foundation Trust	London, UK	Co-Investigator	
Martin	John			St Thomas' Hospital, Guy's and St Thomas' NHS Foundation Trust	London, UK	Co-Investigator	
Kariem	El-Boghdadly			St Thomas' Hospital, Guy's and St Thomas' NHS Foundation Trust	London, UK	Co-Investigator	
Maame	Aduse-Poku			St Thomas' Hospital, Guy's and St Thomas' NHS Foundation Trust	London, UK	Research Nurse	
Gary	Colville			St Thomas' Hospital, Guy's and St Thomas' NHS Foundation Trust	London, UK	Research Nurse	
Gillian	Radcliffe			St Thomas' Hospital, Guy's and St Thomas' NHS Foundation Trust	London, UK	R&D Manager (contracts)	
Xioayu	Zhang			King's College London	London, UK	Health Economist	
Elisabeth	Fell			King's College London	London, UK	Contract Liaison	
Rachel	Fraser			Golden Jubilee University National Hospital, NHS Golden Jubilee	Glasgow, Scotland, UK	Co-Investigator	

*First name, last name, and suffix (if applicable) are required and will appear in PubMed.

*First Name and Middle Initial(s)	*Last Name	*Suffix (eg, Jr, III)	Academic Degrees	Institution	Location (city, state/province, country)	Role or Contribution, eg, chair, principal investigator	Group (if more than 1 Group listed in the byline) and/or Subgroup (eg, Steering Committee)
Cara	Hughes			Golden Jubilee University National Hospital, NHS Golden Jubilee	Glasgow, Scotland, UK	Co-Investigator	
Philip	McCall			Golden Jubilee University National Hospital, NHS Golden Jubilee	Glasgow, Scotland, UK	Co-Investigator	
Christine	Aitken			Golden Jubilee University National Hospital, NHS Golden Jubilee	Glasgow, Scotland, UK	Research Nurse	
Jocelyn	Barr			Golden Jubilee University National Hospital, NHS Golden Jubilee	Glasgow, Scotland, UK	Research Coordinator	
Elizabeth	Boyd			Golden Jubilee University National Hospital, NHS Golden Jubilee	Glasgow, Scotland, UK	Research Nurse	
Julie	Buckley			Golden Jubilee University National Hospital, NHS Golden Jubilee	Glasgow, Scotland, UK	Research Nurse	
Patricia	Griffen			Golden Jubilee University National Hospital, NHS Golden Jubilee	Glasgow, Scotland, UK	Research Nurse	
Charlene	Hamilton			Golden Jubilee University National Hospital, NHS Golden Jubilee	Glasgow, Scotland, UK	Research Nurse	
Ruth	McLaren			Golden Jubilee University National Hospital, NHS Golden Jubilee	Glasgow, Scotland, UK	Research Nurse	
Lesley	Truesdale			Golden Jubilee University National Hospital, NHS Golden Jubilee	Glasgow, Scotland, UK	Research Nurse	
Kathryn	Valdeavella			Golden Jubilee University National Hospital, NHS Golden Jubilee	Glasgow, Scotland, UK	Research Nurse	
Joanna	Ford			Golden Jubilee University National Hospital, NHS Golden Jubilee	Glasgow, Scotland, UK	Research Physiotherapist	
Catherine	Sinclair			Golden Jubilee University National Hospital, NHS Golden Jubilee	Glasgow, Scotland, UK	Head of Research	
Michelle	Keeley			Golden Jubilee University National Hospital, NHS Golden Jubilee	Glasgow, Scotland, UK	Accountant	
Roisin	Houston			Golden Jubilee University National Hospital, NHS Golden Jubilee	Glasgow, Scotland, UK	R&D Manager (contracts)	
Frederick	Cripps			University Hospital of Wales, Cardiff and Vale University Local Health Board	Cardiff, Wales, UK	Research Fellow	
Tristan	Hawkins			University Hospital of Wales, Cardiff and Vale University Local Health Board	Cardiff, Wales, UK	Research Fellow	
Joelle	Healan			University Hospital of Wales, Cardiff and Vale University Local Health Board	Cardiff, Wales, UK	Research Nurse	

Supplemental Online Content: Nonauthor Collaborators
*First name, last name, and suffix (if applicable) are required and will appear in PubMed.

***First Name and Middle Initial(s)**	***Last Name**	***Suffix (eg, Jr, III)**	Academic Degrees	Institution	Location (city, state/province, country)	Role or Contribution, eg, chair, principal investigator	Group (if more than 1 Group listed in the byline) and/or Subgroup (eg, Steering Committee)
John	Kirby			University Hospital of Wales, Cardiff and Vale University Local Health Board	Cardiff, Wales, UK	Research Fellow	
Kerry	Paradowski			University Hospital of Wales, Cardiff and Vale University Local Health Board	Cardiff, Wales, UK	Research Coordinator	
Nirav	Patel			University Hospital of Wales, Cardiff and Vale University Local Health Board	Cardiff, Wales, UK	Research Fellow	
Yashangi	Wagaarachige			University Hospital of Wales, Cardiff and Vale University Local Health Board	Cardiff, Wales, UK	Research Fellow	
Arwen	Hutchings			University Hospital of Wales, Cardiff and Vale University Local Health Board	Cardiff, Wales, UK	R&D Manager (contracts)	
Helen	Paine			University Hospital of Wales, Cardiff and Vale University Local Health Board	Cardiff, Wales, UK	R&D Manager (contracts)	
Mitel	Patel			University Hospital of Wales, Cardiff and Vale University Local Health Board	Cardiff, Wales, UK	R&D Manager (contracts)	
Kathryn	Thomas			University Hospital of Wales, Cardiff and Vale University Local Health Board	Cardiff, Wales, UK	R&D Manager (contracts)	
Frances	Bass			Royal North Shore Hospital, Northern Sydney Local Health District	Sydney, New South Wales, Australia	Research Nurse	
Anne	O'Connor			Royal North Shore Hospital, Northern Sydney Local Health District	Sydney, New South Wales, Australia	Research Nurse	
Elizabeth	Yarad			Royal North Shore Hospital, Northern Sydney Local Health District	Sydney, New South Wales, Australia	Research Nurse	
Iris	Baker-Pearson			The Townsville Hospital, Townsville Hospital and Health Service	Townsville, Queensland, Australia	Research Nurse	
Melanie	White			The Townsville Hospital, Townsville Hospital and Health Service	Townsville, Queensland, Australia	Research Nurse	
Mettassa	Zaro			The Townsville Hospital, Townsville Hospital and Health Service	Townsville, Queensland, Australia	Research Coordinator	
Laura	Adams			The Alfred Hospital, Alfred Health	Melbourne, Victoria, Australia	Research Coordinator	
Thi	Bao Chau			The Alfred Hospital, Alfred Health	Melbourne, Victoria, Australia	Research Nurse	
Grace	Beaton			The Alfred Hospital, Alfred Health	Melbourne, Victoria, Australia	Research Coordinator	
Jasmin	Board			The Alfred Hospital, Alfred Health	Melbourne, Victoria, Australia	Research Nurse	
Emma	Martin			The Alfred Hospital, Alfred Health	Melbourne, Victoria, Australia	Research Coordinator	
Aimee	Neylan			The Alfred Hospital, Alfred Health	Melbourne, Victoria, Australia	Research Manager	
Sarah	Robertshaw			The Alfred Hospital, Alfred Health	Melbourne, Victoria, Australia	Research Coordinator	
Mayumi	Ueoka			The Alfred Hospital, Alfred Health	Melbourne, Victoria, Australia	Research Assisstant	
Sophia	Wallace			The Alfred Hospital, Alfred Health	Melbourne, Victoria, Australia	Research Nurse	

Supplemental Online Content: Nonauthor Collaborators
*First name, last name, and suffix (if applicable) are required and will appear in PubMed.

*First Name and Middle Initial(s)	*Last Name	*Suffix (eg, Jr, III)	Academic Degrees	Institution	Location (city, state/province, country)	Role or Contribution, eg, chair, principal investigator	Group (if more than 1 Group listed in the byline) and/or Subgroup (eg, Steering Committee)
Meredith	Young			The Alfred Hospital, Alfred Health	Melbourne, Victoria, Australia	Research Coordinator	
Simone	Fitzgerald			University Hospital Geelong, Barwon Health	Geelong, Victoria, Australia	Research Coordinator	
Stacey	Hawker			University Hospital Geelong, Barwon Health	Geelong, Victoria, Australia	Research Coordinator	
Joanne	Boone			Curtin School of Population Health, Curtin University	Perth, Western Australia, Australia	Research Coordinator	
Alison	Hodge			Curtin School of Population Health, Curtin University	Perth, Western Australia, Australia	Research Coordinator	
Karen	French			Curtin School of Population Health, Curtin University	Perth, Western Australia, Australia	Study Monitor	
Christine	Robins			Curtin School of Population Health, Curtin University	Perth, Western Australia, Australia	Study Monitor	
Nicholas	Anthony			Fiona Stanley Hospital, South Metropolitan Health Service	Perth, Western Australia, Australia	Research Nurse	
Colin	Garlett			Fiona Stanley Hospital, South Metropolitan Health Service	Perth, Western Australia, Australia	Research Coordinator	
Alea	McLean			Fiona Stanley Hospital, South Metropolitan Health Service	Perth, Western Australia, Australia	Research Coordinator	
Janet	Ferrier			St John of God Hospital Subiaco, St John of God Healthcare	Perth, Western Australia, Australia	Research Nurse	
Farisha	Ali			Auckland City Hospital, Te Whatu Ora - Health New Zealand	Auckland, Auckland, New Zealand	Research Coordinator	
Katie	Brooks			Auckland City Hospital, Te Whatu Ora - Health New Zealand	Auckland, Auckland, New Zealand	Research Nurse	
Magdalena	Butler			Auckland City Hospital, Te Whatu Ora - Health New Zealand	Auckland, Auckland, New Zealand	Research Nurse	
Keri-Anne	Cowdrey			Auckland City Hospital, Te Whatu Ora - Health New Zealand	Auckland, Auckland, New Zealand	Research Nurse	
Eileen	Gilder			Auckland City Hospital, Te Whatu Ora - Health New Zealand	Auckland, Auckland, New Zealand	Research Nurse	
Alix	Gray			Auckland City Hospital, Te Whatu Ora - Health New Zealand	Auckland, Auckland, New Zealand	Research Nurse	
Su-Zahn	Koorts			Auckland City Hospital, Te Whatu Ora - Health New Zealand	Auckland, Auckland, New Zealand	Research Coordinator	
Brittany	Mason			Auckland City Hospital, Te Whatu Ora - Health New Zealand	Auckland, Auckland, New Zealand	Research Nurse	
Ellie	McMahon			Auckland City Hospital, Te Whatu Ora - Health New Zealand	Auckland, Auckland, New Zealand	Research Nurse	

Supplemental Online Content: Nonauthor Collaborators
*First name, last name, and suffix (if applicable) are required and will appear in PubMed.

***First Name and Middle Initial(s)**	***Last Name**	***Suffix (eg, Jr, III)**	Academic Degrees	Institution	Location (city, state/province, country)	Role or Contribution, eg, chair, principal investigator	Group (if more than 1 Group listed in the byline) and/or Subgroup (eg, Steering Committee)
Karina	O'Connor			Auckland City Hospital, Te Whatu Ora - Health New Zealand	Auckland, Auckland, New Zealand	Research Coordinator	
Melissa	Robertson			Auckland City Hospital, Te Whatu Ora - Health New Zealand	Auckland, Auckland, New Zealand	Research Coordinator	
Laura	Vui			Auckland City Hospital, Te Whatu Ora - Health New Zealand	Auckland, Auckland, New Zealand	Research Nurse	
Laura	Weiss			Auckland City Hospital, Te Whatu Ora - Health New Zealand	Auckland, Auckland, New Zealand	Research Nurse	
