# Supplementary material for: High-Flow Nasal Oxygen Therapy After Cardiac Surgery: A Randomized Clinical Trial
Source: JAMA Netw Open. 2026 Apr 8;9(4):e265447. doi: 10.1001/jamanetworkopen.2026.5447 (PMC13063085; doi:10.1001/jamanetworkopen.2026.5447)
Supplement: Supplement 4. — Data Sharing Statement [file jamanetwopen-e265447-s004.pdf]

Data Sharing Statement

Litton. High-Flow Nasal Oxygen Therapy After Cardiac Surgery. *JAMA Netw Open*. Published April 08, 2026. doi:10.1001/jamanetworkopen.2026.5447

Data
Additional Information: Trial registration number: ISRCTN14092678, https://doi.org/10.1186/ISRCTN14092678
Data available: Yes
Data types: Deidentified participant data
How to access data: contact Andrew.klein@nhs.net
When available: With publication

Supporting Documents
Document types: None

Additional Information
Who can access the data: researchers whose proposed use of the data has been approved
Types of analyses: for a specified purpose
Mechanisms of data availability: after ethical approval
